# A review of the potential of lichen substances as antifungal agents: the effects of extracts and lichen secondary metabolites on *Fusarium* fungi

**DOI:** 10.1007/s00203-022-03104-4

**Published:** 2022-07-26

**Authors:** Łukasz Furmanek, Paweł Czarnota, Mark R. D. Seaward

**Affiliations:** 1 Department of Ecology and Environmental Protection, University of Rzeszów, ul. Zelwerowicza 4, 35-601 Rzeszow, Poland; 2 School of Archaeological and Forensic Sciences, University of Bradford, Bradford, BD7 1DP UK

**Keywords:** Antibiotics, Antifungal potential, Allelopathy, *Fusarium*, Lichen substances, Inhibition

## Abstract

The present meta-analysis provides literature data on the effect of lichen extracts and single secondary metabolites used against *Fusarium* spp. moulds. Lichen extracts were obtained from 51 corticolous, 17 terricolous and 18 saxicolous lichen species and 37 secondary compounds were tested against eight fungal species, i.e., *Fusarium acuminatum*, *F. avenaceum*, *F. culmorum*, *F. fujikuroi*, *F. oxysporum*, *F. roseum*, *F. solani* and *F. udum*. The researchers used several test methods, mostly to determine MIC and IZ. Extracts were obtained using several solvents, mainly organic ones with use of the Soxhlet apparatus. The most frequently tested species was *F. oxysporum*, against which lichen substances from *Alectoria sarmentosa*, *Cladonia mitis*, *C. rangiferina*, *Flavoparmelia caperata*, *Hypotrachyna cirrhata*, *Leucodermia leucomelos*, *Parmotrema austrosinense*, *P. reticulatum*, *Physcia aipolia*, *Pseudevernia furfuracea*, *Roccella montagnei* and *Umbilicaria nylanderiana* and secondary metabolites such as 2-hydroxy-4-methoxy-3,6-dimethylbenzoic acid, atranorin, lecanoric and (+)-usnic acids showed the highest antifungal potential. These agencies could compete with the potential of fungicides, such as flucytosine and fluconazole. Other species have been poorly investigated. Statistical analysis of literature data showed that the fungistatic potential of lichen extracts is significantly different from individual secondary metabolites. Similarly, the potential of secondary metabolites often differs significantly from that of non-lichen substances. This meta-analysis indicates the potential of lichen substances as future anti-fusarial agents.

**Supplementary Information:**

The online version contains supplementary material available at 10.1007/s00203-022-03104-4.

## Introduction

Lichens are an ecosystem complex (Hawksworth and Grube 2020) that relies upon a symbiosis resulting from the co-relation of a heterotrophic fungus and an autotrophic photosynthetic component (Elkhateeb et al. 2020; Jüriado et al. 2019). The fungus most often represents the Ascomycota division (Wang et al. 2014), less frequently the Basidiomycota one (Lücking et al. 2017), while the photobiont belongs to the Chlorophyta or Cyanobacteria division (Bates et al. 2011). This complex, in the cortical layer, can be enriched by basidiomycete fungi (Spribille et al. 2016; Tuovinen et al. 2019), bacteria (Bates et al. 2011; Grube et al. 2009, 2015) or other fungi involved in this relationship (Agrawal et al. 2020; Kellogg and Raja 2017; Yazıcı et al. 2020). Endolichenic fungi may be important for the qualitative substance profile of the lichen symbiosis (Agrawal et al. 2020; Kellogg and Raja 2017), as might the presence of lichenicolous fungi altering the antioxidant potential of the extract obtained (Khadhri et al. 2019). Due to the dominant role of the fungus (Muggia and Grube 2018), the lichen relationship is classified from the perspective of the Kingdom Fungi.

The establishment of this relationship results not only in the formation of the diverse morphology of the lichen thallus, but also in the metabolism of such a symbiosis (Furmanek 2019; Furmanek et al. 2019). Primary metabolism pathways produce basic substances constituting the structure of bionts, such as proteins, pigments, and vitamins (Shukla et al. 2018) or polysaccharides (Cordeiro et al. 2003) that bind the lichen’s structure (Spribille et al. 2020). Their bioproduction occurs in both lichen components, with no direct way to identify the source of their origin (Ranković and Kosanić 2015). Many of which can be dissolved using hot water (Elix and Stocker-Wörgötter 2008; Surayot et al. 2019). The extraction of polysaccharides in different yields depends upon the lichen species (Carbonero et al. 2001; Cordeiro et al. 2003), but with similar chemical structures of sugars, probably independent of their habitat (Cordeiro et al. 2003). Uniquely, isolichenin requires only a low-temperature extraction process (Surayot et al. 2019). Polysaccharide biosynthesis may result from both the presence of a particular mycobiont (Cordeiro et al. 2004; Ruthes et al. 2008) or from the complicity of a photobiont (isolichenan: Cordeiro et al. 2004; Ruthes et al. 2008).

The aposymbiotic form of both components leads to qualitatively different polysaccharides (Cordeiro et al. 2005, 2007). Their solubility is mainly determined by the specific bond ratios (1 → 3) to (1 →  4)-α-d-glucan with a ratio range of 1:1 to 1:4 (Cordeiro et al. 2003). These factors affect the sugar content, which can account for up to c. 57% of the extracted compounds in the mixture of lichen substances (Akbulut and Yildiz 2010; Boustie et al. 2011; Furmanek et al. 2022).

Modest experiments showed that polysaccharides possess anticancer, antiviral (Olafsdottir and Ingólfsdottir 2001 and literature cited therein) or immunostimulatory (Surayot et al. 2019; Zhang et al. 2016) or anti-coagulant properties (see broader: Ullah et al. 2019 and literature cited therein), although lichenin did not significantly alter the mycelial growth dynamics of plant root-sphere fungi (Ruotsalainen et al. 2009).

The photobiont, in exchange for its protection by the mycobiont, provides useful carbohydrates which are converted by the mycobiont, specific for the particular symbiosis, to secondary metabolites (Furmanek 2019; Staśkiewicz and Szakiel 2020); these are deposited in the form of crystals on the outer part of mycobiont or inside the fungal cell wall (Grimm et al. 2021) and are thought to be rather insoluble in water (Furmanek et al. 2022; Staśkiewicz and Szakiel 2020), such as usnic acid (Jin et al. 2013), especially at a pH less than 7 (Honegger 2009), although an increase in temperature facilitates the passage of usnic acid into the extract (Jin et al. 2013), as do polar functional groups attached to secondary compounds (Furmanek et al. 2022; Rundel 1978). The solubility of lichen phenols in water has been increasingly reported (Zagoskina et al. 2013; Zavarzina et al. 2019). Lichen compounds are most efficiently extracted with organic solvents, although deeper analysis indicates more complex relationships; see chemical profiles, for example, in Jin et al. (2013), Millot et al. (2017), Tekiela et al. (2021) and Voicu et al. (2019). The role of secondary metabolites is primarily a protective function of the lichen thallus against stress factors (Legouin et al. 2017; Veres et al. 2020); these may increase its dry weight (Farkas et al. 2020; Proko’ev et al. 2018), by more than 50% in the case of a single secondary compound such as physodic acid included in a substances mixture (Latkowska et al. 2019). The use of an organic solvent and changes in its pH can determine the chemical structure of the secondary metabolites, as in the case of atranorin (Vos et al. 2018).

The most prominent secondary metabolism pathway of the symbiosis is the acetate-polymalonate pathway, which involves the biosynthesis of most of the secondary metabolites classified into various biochemical classes, including those analysed in this review. These include, for example, monocyclic aromatic derivatives, orcinol depsides, β-orcinol depsides, orcinol depsides, β-orcinol depsides, usnic acid derivatives or aliphatic acids biosynthesized via the tricarboxylic acid cycle. The biosynthesis of structurally branched secondary compounds occurs in the mevalonic acid pathway, which includes metabolites belonging to the class of terpenoids or sterols. The shikimic acid pathway is characterized by the biosynthesis of mainly pulvinic acid derived compounds (Furmanek et al. 2022; Goga et al. 2018; Staśkiewicz and Szakiel 2020). Elix (2014) distinguishes c. 45 biochemical classes of lichen secondary metabolites consisting of more than 850 compounds.

Laboratory in vitro studies on the potential of lichen secondary metabolites report their broad biological properties such as anticancer (Cardile et al. 2017; Tripathi et al. 2022 and literature cited therein), antioxidant (Hawrył et al. 2020; Maulidiyah et al. 2021), antimicrobial (Shiromi et al. 2021; Sargsyan et al. 2021), pro-health (Zhao et al. 2021 and literature cited therein) or anti-fusarial potentials described in this meta-analysis.

Species in the *Fusarium* genus (Ascomycota) are ecological plant pathogens and saprotrophs (Dignani and Anaissie 2004; Karim et al. 2016). Divergent estimates state that there are more than 300 (Dongzhen et al. 2020) to as many as 1,500 species in this genus (Arie 2019), which are identified by their shape, cell number, and mode of division. The genus is widespread (Arie 2019), forming a bright and flocculent mycelium in different shades of colour of small conidiophores (Chróst 2016). Macroconidia of *Fusarium* spp. species are curved and multicellular (Dignani and Anaissie 2004; Pitt and Hocking 2009). Their spores spread over considerable distances, and the genetic similarity of analogues of environmental and clinical isolates (Batista et al. 2020) increases their infectivity potential of living organisms, although their susceptibility to currently used antibiotics is partly dependent on the source of their isolation (environment or patient) (Pujol et al. 1997).

Of particular interest is the ability of *Fusarium* spp. pathogens to infect plants, such as *F. avenaceum*, *F. fujikuroi*, *F. oxysporum* and *F. solani* described in this review; these damage crop plants, e.g., tomato wilt (Isaac et 2018), yam wilt (Dongzhen et al. 2020), banana wilt (Ujat et al. 2021), wheat seed (Köycü 2018) and ear rot in maize (Oldenburg et al. 2017) or cause tree infections (*Pinus sylvestris*, *Alnus glutinosa* and *Larix decidua*) (Tekiela et al. 2021). Contamination of grains is the reason for their loss of quality (Nelson et al. 1994) or food products (Ji et al. 2019; Oldenburg et al. 2017).

Increased resistance of fungal complexes of *F. fujikuroi*, *F. oxysporum* and *F. solani* species may cause human disorders (see: Batista et al. 2020; Dignani and Anaissie 2004; Herkert et al. 2019; Nelson et al. 1994; Song et al. 2021), most commonly onychomycosis, skin infections and keratitis (see: Batista et al. 2020 and literature cited therein; Herkert et al. 2019); they also contribute to systemic conditions, including disorders of the nervous system (see: Dignani and Anaissie 2004), the heart (endocarditis: Esnakula et al. 2013), and tissues, such as the skin, lungs and sinuses (Dignani and Anaissie 2004). Their presence may be the cause of the so-called sick-building syndrome (Li and Yang 2004; Chróst 2016; Nag 2019), and together with the mycotoxins produced by *Fusarium* (Dignani and Anaissie 2004), can negatively affect the quality of food products and the health of humans (Köycü 2018; Nelson et al. 1994).

The most dangerous *Fusarium* species from a phytopharmacological point of view are *F. culmorum*, *F. graminearum* (Köycü 2018), *F. fujikuroi* (Nelson et al. 1994) and *F. oxysporum* (Arie 2019), while the most pathogenic from a medical point of view, such as *F. solani*, can take advantage of a weakened barrier of the human immune system (Mansoory et al. 2003; Pujol et al. 1997).

In view of the increasing need for new antibiotic substances and in the context of declining possibilities against *Fusarium* pathogens, (Batista et al. 2020; Esnakula et al. 2013; Guarro et al. 1999; Wolny-Koładka 2016), this review underlines the concept of green chemistry, with the potential use of natural lichen-derived substances presenting. This is especially true when considering the economic implications (Batista et al. 2020; Dongzhen et al. 2020), food security (Arie 2019; Köycü 2018), and even risks to swine (see: Nelson et al. 1994) and domestic animals (Witaszak et al. 2019).

Given the above and based on the results obtained, as well as the conclusions drawn, the aim of this paper is to present new perspectives on the control of excessive growth of the genus *Fusarium* species in residential and public buildings, and the search for and potential use of lichen substances as future plant protection agents (fungicides) and as food preservatives. For medical and veterinary practitioners, their potential as agents of therapeutic value in the treatment of human disease and as a replacement for currently used antibiotics should be of particular interest. Despite this relatively under-researched area, the results obtained from this meta-analysis demonstrate their future potential.

## Materials and methods

### General issues

This literature review is complementary to previous studies on the effects of extracted complexes of lichen substances and their secondary metabolites on dermatophytes (Furmanek et al. 2019 and literature cited therein) and genus *Aspergillus* spp. (Furmanek et al. 2022 and literature cited therein). The overall design of this review is mainly based on the latter article, with the main difference being the statistical analysis of the literature data (meta-analysis).

The collected literature data on the effects of extracted substances concern 51 corticolous species, 17 terricolous species, and 18 saxicolous lichen species (Tab. S1). The 37 isolated secondary metabolites of lichens belonging to 13 biochemical classes have been tested (Tab. S2). Lichen extracts and their secondary metabolites were investigated against eight *Fusarium* species, namely *F. acuminatum* Ellis & Everh., *F. avenaceum* (Fr.) Sacc., *F. culmorum* (Wm.G. Sm.) Sacc, *F. fujikuroi* Nirenberg [syn. *F. moniliforme* J. Sheld., *F. subglutinans* (Wollenw. & Reinking) P.E. Nelson, Toussoun & Marasas, *F. verticillioides* (Sacc.) Nirenberg], *F. oxysporum* Schltdl., *F. roseum* Link, *F. solani* (Mart.) Sacc. and *F. udum* E.J. Butler. Data reported in the literature for *Fusarium tabacinum* (J.F.H. Beyma) W. Gams have not been included here, since this species is now recognized as *Plectosphaerella cucumerina* (Lindf.) W. Gams. Species names are given according to *Index Fungorum* (status on: 11.11.2021).

The lichen species were divided into ecological groups according to the substrate; if more than one group was identified, then the dominant one was selected. Such a division was intended to facilitate data compilation and interpretation as well as comparative analysis and meta-analysis of the antifungal potential of lichen extracts growing on different substrates (epiphytes, epigeics and epilithics) (Tab. S1). Similarly, the division of lichen secondary metabolites was based on of their belonging to a specific biochemical class (after: Elix 2014) for more practical compilation and analysis of the collected data (Tab. S2).

Due to the different methodologies employed by researchers to extract substances from lichens, only the main extracting solvent is given in the tables presented in this review; however, if necessary, this issue was described in more detail in the discussion for the most effective methodological combinations. The data collected indicate the use of 11 solvents to obtain lichen extracts, the most frequently used being methanol and acetone. Chloroform, ethanol, water and ethyl acetate were used less frequently, and diethyl ether, hexane, 2-propanol, petroleum ether and dichloromethane were used least of all. For data on the anti-fusarial potential of secondary metabolites, only the major extracting solvents are mentioned.

Test results were most often reported as Minimal Inhibitory Concentration (MIC) and Inhibition Zone (IZ). In a few cases, the extract concentration was reported as Minimal Fungicidal Concentration (MFC). Occasionally, results were given as a percentage measure of mycelial growth dynamics (RG%), percentage inhibition of mycelial growth (IR%), percentage inhibition of spore germination (SGI), and dose resulting in inhibition of mycelial growth dynamics at 50% efficiency level (ED_50_).

To determine the MIC values, test methods such as disk diffusion method (DDM) (see: Sarıözlü et al. 2016; Türk et al. 2006), Broth tube dilution method (BTDM) (see: Ranković and Mišić 2007; Babiah et al. 2014a), Broth microdilution method (BMM) (see: Aslan et al. 2006; Kosanić and Ranković 2011a), Broth microdilution method with 2,3,5-triphenyltetrazolium chloride (BMMwTTC) (see: Shivanna and Garampalli 2015), and Microdilution method with resazurin (MMwR) (see: Sarıözlü et al. 2016; Cankılıç et al. 2017) have been used. BTDMs were used to determine MFC (see: Ranković and Mišić 2007). DDM was applied for IZ (see: Aslan et al. 2006; Sarıözlü et al. 2016) and Agar well diffusion method (AWDM) (see: Ranković and Mišić 2007; Shivanna and Garampalli 2015). Other methods for measuring mycelial growth included poisoned food technique (PFT) (see: Halama and van Haluwin 2004; Karabulut and Ozturk 2015; Tekiela et al. 2021) and Spore germination inhibition (SGI) (see: Shahi et al. 2003). The most frequently used methods were DDM and BTDM, while BMMwTTC, MMwR, AWDM, BMM and PFT were used less frequently; SGI was only used in a few cases. If the researchers did not directly state the name of the research method used (i.e., PFT; Tekiela et al. 2021) or there were some discrepancies in the names [i.e., Kirby and Bauer disk diffusion method—as DDM (i.e., Babiah et al. 2014a) or Microbroth dilution method—as MMwR (i.e., Sarıözlü et al. 2016)], they were classified according to the method employed. Professional descriptions of BTDM, BMM and DDM are given by the Clinical and Laboratory Standards Institute (CLSI 2015a, b; 2017).

The concentration unit for the test extracts, secondary metabolites and antifungal non-lichen drugs was given as mg ml^−1^; in some cases, these were converted to standardize the presented results. It was not always possible to give the concentration or dose of the substances used; this information is mentioned in the text, where necessary.

Determination of the inhibitory potential level of the tested extracted lichen compounds as extracts and individual secondary metabolites was conducted by comparing them to the antifungal potential of reference substances against *Fusarium* spp. The greatest attention in the results and discussion sections was paid to the description of combinations that could compete with the effectiveness of the antibiotics.

Several tables are provided as supplementary material on the journal’s official website.

### Statistics

Statistical analysis was performed for positive Minimal Inhibitory Concentrations and Inhibition Zone results if they met the minimum number (*N* = 5) of data collected for extracts or secondary metabolites of lichen and non-lichen antifungal references in the variants. This abundance was considered to be the minimum for statistical analysis and for drawing any conclusions regarding the potential of the analysed substances. Therefore, statistical analysis was carried out using data concerning extracts of all the three lichen ecological groups against *Fusarium oxysporum* and from corticolous lichen species against *F. solani*, and secondary metabolites tested against *F. fujikuroi* as well as *F. oxysporum*.

An analogous criterion was applied to non-lichen antifungal agents, although in some cases, cited MIC values were given collectively for independently performed experiments. MIC results of reference substances with a majority sign (>) were not included in the statistical analysis. Where a range of MIC values is given in a table, the smallest and largest values from the given range were performed in the statistical analysis.

Statistical analysis did not take into account methodological differences in the cited experiments and the use of various strains of a given *Fusarium* species, as this would have prevented analysis and conclusions.

The statistics of normal distribution were checked by the *W* Shapiro–Wilk test and the *λ* Kolmogorov–Smirnov test with Lilliefors correction (for *N* < 30) and by the *X*^2^ test and the *λ* Kolmogorov–Smirnov test (for *N* ≥ 30). Because the assumption of normality of data distribution was not met, tests to check homogeneity of variance were not performed. Statistical significance was checked by using non-parametric tests for two groups: *U* Mann–Whitney and *λ* Kolmogorov–Smirnov. A significance level of *α* = 0.05 was used for all tests used. A meta-analysis of the collected literature data was performed for all possible combinations to meet the above criteria.

## Results

### Analyses of extracted-lichen compounds of various lichen thalli

#### Potential of crude extracts against *Fusarium acuminatum* (references: Tab. S3)

The tested mixtures of lichen substances were extracted from six corticolous species (*Evernia divaricata*, *E. prunastri*, *Parmelia saxatilis*, *Platismatia glauca*, *Ramalina pollinaria* and *R. polymorpha*), two terricolous species (*Cladonia foliacea* and *C. rangiformis*) and three saxicolous species (*Dermatocarpon miniatum*, *Xanthoparmelia pulla* and *Umbilicaria nylanderiana*). The only effective potential of lichen substances was found for the methanol extract from *E. divaricata* (MIC: 62.5 × 10^–3^ mg ml^−1^), confirmed by a high IZ (30 mm). The other extracts were found to be ineffective according to the IZ values.

#### Potential of crude extracts against *Fusarium avenaceum* (references: Tab. S4)

Tekiela et al. (2021) tested six combinations using corticolous and terricolous lichen species. The highest level of relative inhibition of mycelial growth dynamics was measured for acetone-extracted substances from *Cladonia mitis* (6.6% relative mycelial growth compared to mycelial dimensions in the control sample) after 180 h of the experiment. A slightly weaker result (c. 13–16%) was obtained with acetone extract from *C. rangiferina* and ethanol extract from mixed *C. mitis* + *C. rangiferina* lichen species.

#### Potential of crude extracts against *Fusarium culmorum* (references: Tab. S5)

Lichen substances extracted from three corticolous (*Evernia prunastri*, *Parmelia sulcata*, *Pseudevernia furfuracea*) and two terricolous (*Cetratria aculeata* and *Cladonia foliacea*) species were tested. According to the non-standard MIC value, the acetone extract of *P. furfuracea* achieved the highest fungistatic potential (320–597 µg/disk) (Türk et al. 2006). A mixture of ethanol-extracted substances from *Evernia prunastri* was fully effective (100% inhibition) (Karabulut and Ozturk 2015). Extracts from the terricolous species showed no activity (MIC, IZ).

#### Potential of crude extracts against ***Fusarium fujikuroi*** (references: Table 1)

**Table 1:** Effect of crude extracts from corticolous, terricolous and saxicolous lichens on *Fusarium fujikuroi*

Ecological group	Lichen species	Extracting solvent	Results			Measurement method	New literature
			MIC [mg ml^−1^]	MFC [mg ml^−1^]	IZ [mm] (dose)		
Corticolous lichens	*Bryoria capillaris*	Acetone	156.2 × 10^–3^	^b^	11 (conc. 0.833 mg/disk)	DDM	[1]
		Chloroform	^b^	^b^	^a^		
		Methanol	312.5 × 10^–3^	^b^	15 (conc. 0.833 mg/disk)		
	*Parmotrema andinum*	Acetone	^b^	^b^	8.33 (15 µl) (conc.200 mg 2 ml^−1^)	DDM	[2]
		2-Propanol	^b^	^b^	20.66 (15 µl) (conc. 200 mg 2 ml^−1^)		
	*Parmotrema tinctorum*	2-Propanol	^b^	^b^	6 (15 µl) (conc. 100 mg ml^−1^)	DDM	[3]
	*Pseudevernia furfuracea*	Acetone (test sample 1 and 2)	597 µg/disk (conc. 5.6 mg ml^−1^)	^b^	^b^	DDM	[4]
			640 µg/disk (conc. 6 mg ml^−1^)	^b^	^b^		
		Chloroform (test sample 1 and 2)	787 µg/disk (conc. 7.4 mg ml^−1^)	^b^	^b^	DDM	[4]
			1813 µg/disk (conc. 8.5 mg ml^−1^)	^b^	^b^		
		Ethanol (test sample 1 and 2)	1460 µg/disk (conc. 6.8 mg ml^−1^)	^b^	^b^	DDM	[4]
			400 µg/disk (conc. 15 mg ml^−1^)	^b^	^b^		
	*Ramalina farinacea*	Acetone	^b^	^b^	^a^	DDM	[5]
	*Ramalina nervulosa*	Acetone	^b^	^b^	^a^		
	*Ramalina roesleri*	Acetone	^b^	^b^	^a^		
	*Usnea florida*	Acetone	^b^	^b^	^a^	DDM	[6]
		Chloroform	^b^	^b^	^a^		
		Methanol	^b^	^b^	^a^		
Terricolous lichens	*Cetraria aculeata*	Acetone	^b^	^b^	^a^	DDM	[7]
		Diethyl ether	^b^	^b^	^a^		
		Ethanol	^b^	^b^	^a^		
	*Cladonia foliacea*	Acetone	^a^	^b^	^b^	DDM	[8]
		Chloroform	^a^	^b^	^b^		
		Diethyl ether	^a^	^b^	^b^		
		Ethanol	^a^	^b^	^b^		
		Petroleum Ether	^a^	^b^	^b^		
	*Gyalolechia subbracteata*	Methanol	^b^	^b^	30.33 (15 µl) (conc. 1000 mg ml^−1^)	DDM	[9]
	*Catapyrenium squamulosum*	Methanol	^b^	^b^	5 (uncertain: 15 µl) (conc. 1000 mg ml^−1^)	DDM	[10]
	*Psora decipiens*	Methanol	^b^	^b^	^a^	DDM	[9]
Saxicolous lichens	*Blennothallia crispa*	Methanol	^b^	^b^	28 (uncertain: 15 µl) (conc. 1000 mg ml^−1^)	DDM	[10]
	*Protoparmeliopsis muralis*	Methanol	^b^	^b^	^a^	DDM	[9]
	*Pyrenodesmia variabilis*		^b^	^b^	27.33 (15 µl) (conc. 1000 mg ml^−1^)		

Method of measurement abbreviations: *DDM* disk diffusion method (MIC, IZ). For MIC, MFC and IZ abbreviations: see Table 3. Literature abbreviations: [1] Sarıözlü et al. (2016); [2] Anjali et al. (2015a); [3] Anjali et al. (2015b); [4] Türk et al. (2006); [5] Gazo et al. (2019); [6] Cankılıç et al. (2017); [7] Türk et al. (2003); [8] Yılmaz et al. (2004); [9] Valadbeigi et al. (2014); [10] Valadbeigi and Shaddel (2015)

^a^No effect

^b^Not investigated

Mixtures of substances from 16 lichen species (eight corticolous, five terricolous and three saxicolous) were tested. According to the MIC, the acetone extract from *Bryoria capillaris* proved to be the most effective, with the analogous methanol extract being half that strength (Sarıözlü et al. 2016). An alternative approach for MIC values indicates that an ethanol-extracted mixture of lichen substances from *Pseudevernia furfuracea* is a potentially effective fungistatic agent (Türk et al. 2006). The largest zone of inhibition was measured for methanol extracts from epigeic species of *Gyalolechia subbracteata* (Valadbeigi et al. 2014) and the saxicolous *Blennothallia crispa* (Valadbeigi and Shaddel 2015) and *Pyrenodesmia variabilis* (Valadbeigi et al. 2014).

#### Potential of crude extracts from corticolous lichens against *Fusarium oxysporum* (references: Tab. S6)

Mixtures of lichen substances extracted from 48 corticolous species have been tested. A MIC value below 1 ml mg^−1^ was achieved by one of a methanol extract from *Flavoparmelia caperata* (0.097 mg ml^−1^), although its inhibitory potential according to IZ does not appear to be significant (IZ: 13.3 mm) (Shivanna and Garampalli 2014). Methanol extract from *Parmotrema austrosinense* (MIC: 0.39 mg ml^−1^; IZ: 21.3 mm), methanol and ethyl acetate extracts from *Physcia aipolia* (MIC: 0.39 mg ml^−1^; IZ: 12.3–12.6 mm) and *Roccella montagnei* (MIC: 0.39 mg ml^−1^; IZ: 11.3–13 mm) (Shivanna and Garampalli 2014) also reached this level.

It is interesting to note that the mixture of substances present in the ethanol and ethyl acetate extracts from *Alectoria sarmentosa* had a low inhibitory potential against *F. oxysporum* (MIC 50 mg ml^−1^ and 100 mg ml^−1^, respectively), but the combinations become valuable in light of the fungicidal potential, MFC, respectively: 100 mg ml^−1^ and 150 mg ml^−1^ (Ranković and Mišić 2007). The largest diameter of the mycelial growth inhibition zone was measured for ethanol from *Roccella montagnei* (IZ: 32 mm) (Devashree et al. 2019) and acetone extracts from *Parmotrema perlatum* (IZ: 30 mm) (Thippeswamy et al. 2013).

Acetone extracts from *Pseudevernia furfuracea* also appear to be noteworthy, being effective for a MIC concentration at 597–640 µg/disk (Türk et al. 2006). The water-extracted substances mixture from *Hypotrachyna cirrhata* (Shahi et al. 2003) and *Leucodermia leucomelos* (Shahi et al. 2001) was found to have fungicidal efficacy according to the SGI method (concentration: 80 µl ml^−1^). The percentage inhibition rates of mycelial growth dynamics (IR%) reached maximum of 68% inhibition of mycelium growth exhibited by the potential of substances included in acetone extract from *Parmotrema reticulatum* (Babiah et al. 2014b). Based on the ED_50_ values of substances extracted from *P. reticulatum*, ethyl acetate (ED_50_: 43.7 µg ml^−1^) (Goel et al. 2011a) was the best solvent in terms of inhibitory effects.

#### Potential of crude extracts from terricolous lichens against *Fusarium oxysporum* (references: Tab. S7)

The inhibitory potential was investigated for extracts from 13 terricolous lichen species. The strongest potential was obtained for the methanol extract from *Cetraria islandica* (MIC: 2.5 mg ml^−1^) (Grujičić et al. 2014). The use of ethanol and ethyl acetate to extract substances from *Cladonia rangiferina* may determine fungistatic and fungicidal effects at the same concentrations of 100 mg ml^−1^ and 150 mg ml^−1^, respectively; however, the IZ value may not support this (IZ:13–14 mm) (Ranković and Mišić 2007).

Mycelial growth dynamics over time (PFT) shows the fungistatic potential of acetone extract from *C. rangiferina* and *C. mitis*, which reduced mycelial growth dynamics to more than one-fifth of the mycelial diameter compared to the control sample after 180 h of the experiment. The use of ethanol extract from *C. mitis*, acetone and ethanol extracts from mixed *C. mitis* + *C. rangiferina* and ethanol extract from *C. rangiferina* attenuated the inhibition potential to c. 40–50% of relative mycelial growth (Tekiela et al. 2021).

Lichen substances extracted with hexane from *Stereocaulon himalayense* inhibited mycelial growth at a concentration of ED_50_: 66.54 µg ml^−1^ (Goel et al. 2011a).

#### Potential of crude extracts from saxicolous lichens against *Fusarium oxysporum* (references: Tab. S8)

Extracts were obtained from 15 species of saxicolous lichens. In the light of MIC values, the methanol extract from *Umbilicaria nylanderiana* (62.5 × 10^–3^ mg ml^−1^) exhibited outstanding potential, which was also confirmed by the highest IZ value (33 mm) (Gulluce et al. 2006). The substances extracted with methanol from *U. polyphylla* (MIC: 1.56 mg ml^−1^; IZ: 22 mm) (Ranković et al. 2009) also seem to be among the more potent ones.

#### Potential of crude extracts from lichens against *Fusarium roseum* (references: Tab. S9)

The extracts obtained from five corticolous lichen species have been tested. The determination of the fungistatic potential of the extracts has so far been based only on the zone of growth inhibition. The highest IZ value, reaching over 20 mm in diameter, was obtained with substances extracted with methanol from *Parmotrema thomsonii* (Tiwari et al. 2011a).

#### Potential of crude extracts from lichens against ***Fusarium solani*** (references: Table 2)

**Table 2:** Effect of crude extracts from corticolous, terricolous and saxicolous lichens on *Fusarium solani*

Ecological group	Lichen species	Extracting solvent	Results				Measurement method	Literature
			MIC [mg ml^−1^]	MFC [mg ml^−1^]	IZ [mm] (dose)	Others		
Corticolous lichens	*Alectoria sarmentosa*	Ethanol	20	20	25 (20 µl/well)	^b^	BTDM; AWDM	[1]
		Ethyl acetate	50	50	20 (20 µl/well)	^b^		
		Water	50	50	8 (20 µl/well)	^b^		
	*Bryoria capillaris*	Acetone	156.2 × 10^–3^	^b^	13 (conc. 0.833 mg/disk)	^b^	DDM	[2]
		Chloroform	^b^	^b^	^a^	^b^		
		Methanol	312.5 × 10^–3^	^b^	15 (conc. 0.833 mg/disk)	^b^		
	*Bulbothrix setschwanensis*	Acetone	^b^	^b^	c. 11 (5 ml) (conc. 50 mg ml^−1^)	^b^	DDM	[3]
		Chloroform	^b^	^b^	^a^	^b^		
		Methanol	^b^	^b^	c. 8 (5 ml) (conc. 50 mg ml^−1^)	^b^		
	*Evernia divaricata*	Methanol	^b^	^b^	^a^	^b^	DDM	[4]
	*Evernia prunastri*	Methanol	^b^	^b^	^a^	^b^	DDM	[4]
		Acetone	^b^	^b^	^b^	RG%: 61.6	PFT	[5]
		Ethanol	^b^	^b^	^b^	IR%: 73.33 (375 µl) (extract conc.: 10%)	PFT	[6]
	*Hypotrachyna nepalensis*	Acetone	^b^	^b^	^a^	^b^	DDM	[3]
		Chloroform	^b^	^b^	^a^	^b^		
		Methanol	^b^	^b^	c. 16 (5 ml) (conc. 50 mg ml^−1^)	^b^		
	*Flavoparmelia caperata*	Acetone	12.5	^b^	10.3 (50 µl)	^b^	BTDM; DDM	[7]
		Chloroform	12.5	^b^	7 (50 µl)	^b^	BTDM; DDM	[7]
		Ethyl acetate	^b^	^b^	^a^	^b^	AWDM; BMMwTTC	[8]
		Methanol	12.5	^b^	7.6 (50 µl)	^b^	BTDM; DDM	[7]
			1.562	^b^	12.6 (100 µl) (conc. 30 mg ml^−1^)	^b^	AWDM; BMMwTTC	[8]
	*Heterodermia diademata*	Acetone	^b^	^b^	c. 10 (5 ml) (conc. 50 mg ml^−1^)	^b^	DDM	[3]
		Chloroform	^b^	^b^	^a^	^b^		
		Methanol	^b^	^b^	c. 20 (5 ml) (conc. 50 mg ml^−1^)	^b^		
	*Hypogymnia physodes*	Acetone	^b^	^b^	^b^	RG%: 32.3	PFT	[5]
	*Parmelia saxatilis*	Methanol	^b^	^b^	^a^	^b^	DDM	[9]
	*Parmelia sulcata*	Ethanol	^b^	^b^	^b^	IR%: 33.82 (375 µl) (extract conc.: 10%)	PFT	[6]
	*Parmotrema andinum*	Acetone	^b^	^b^	7.33 (15 µl) (conc. 200 mg 2 ml^−1^)	^b^	DDM	[10]
		2-Propanol	^b^	^b^	19 (15 µl) (conc. 200 mg 2 ml^−1^)	^b^		
	*Parmotrema austrosinense*	Ethyl acetate	6.25	^b^	12.3 (100 µl) (conc. 30 mg ml^−1^)	^b^	AWDM; BMMwTTC	[8]
		Methanol	^b^	^b^	^a^	^b^		
	*Parmotrema grayanum*	Ethyl acetate	6.25	^b^	15.3 (100 µl) (conc. 30 mg ml^−1^)	^b^	AWDM; BMMwTTC	[8]
		Methanol	^b^	^b^	^a^	^b^		
	*Parmotrema reticulatum*	Acetone	25	^b^	17 (50 µl)	IR%: 89	BTDM; DDM	[11]
		Chloroform	25	^b^	7.6 (50 µl)	IR%: 50	BTDM; DDM	[11]
		Ethyl acetate	6.25	^b^	17 (100 µl) (conc. 30 mg ml^−1^)	^b^	AWDM; BMMwTTC	[8]
		Methanol	^b^	^b^	^a^	^b^	AWDM; BMMwTTC	[8]
			25	^b^	11 (50 µl)	IR%: 72	BTDM; DDM	[11]
	*Parmotrema tinctorum*	Acetone	^b^	^b^	10.3 (5 ml)	^b^	DDM	[12]
		Chloroform	^b^	^b^	^a^	^b^	DDM	[12]
		Ethyl acetate	1.562	^b^	18.6 (100 µl) (conc. 30 mg ml^−1^)	^b^	AWDM; BMMwTTC	[8]
		Methanol	^b^	^b^	^a^	^b^	AWDM; BMMwTTC	[8]
			^b^	^b^	17.6 (5 ml)	^b^	DDM	[12]
	*Parmotrema thomsonii*	Acetone	^b^	^b^	c. 9 (5 ml) (conc. 50 mg ml^−1^)	^b^	DDM	[3]
		Chloroform	^b^	^b^	c. 18 (5 ml) (conc. 50 mg ml^−1^)	^b^		
		Methanol	^b^	^b^	c. 12 (5 ml) (conc. 50 mg ml^−1^)	^b^		
	*Physcia aipolia*	Ethyl acetate	6.25	^b^	14 (100 µl) (conc. 30 mg ml^−1^)	^b^	AWDM; BMMwTTC	[8]
		Methanol	^b^	^b^	^a^	^b^		
	*Platismatia glauca*	Methanol	^b^	^b^	^a^	^b^	DDM	[9]
	*Ramalina farinacea*	Acetone	^b^	^b^	^a^	^b^	DDM	[13]
	*Ramalina nervulosa*	Acetone	^b^	^b^	^a^	^b^	DDM	[13]
	*Ramalina pollinaria*	Methanol	^b^	^b^	^a^	^b^	DDM	[9]
	*Ramalina polymorpha*	Methanol	^b^	^b^	^a^	^b^	DDM	[9]
	*Ramalica roesleri*	Acetone	^b^	^b^	^a^	^b^	DDM	[13]
	*Roccella montagnei*	Ethyl acetate	6.25	^b^	13.3 (100 µl) (conc. 30 mg ml^−1^)	^b^	AWDM; BMMwTTC	[8]
		Methanol	6.25	^b^	11.6 (100 µl) (conc. 30 mg ml^−1^)	^b^		
	*Teloschistes flavicans*	Ethyl acetate	12.25	^b^	18.6 (100 µl) (conc. 30 mg ml^−1^)	^b^	AWDM; BMMwTTC	[8]
		Methanol	^b^	^b^	^a^	^b^		
	*Pseudevernia furfuracea*	Ethanol	^b^	^b^	^b^	IR%: 21.25 (375 µl) (extract conc.: 10%)	PFT	[6]
		Acetone	597 µg/disk (conc. 5.6 mg ml^−1^)	^b^	^b^	^b^	DDM	[14]
			640 µg/disk (conc. 6 mg ml^−1^)	^b^	^b^	^b^		
		Chloroform	907 µg/disk (conc. 8.5 mg ml^−1^)	^b^	^b^	^b^	DDM	[14]
			1574 µg/disk (conc. 7.4 mg ml^−1^)	^b^	^b^	^b^		
		Ethanol	800 µg/disk (conc. 15 mg ml^−1^)	^b^	^b^	^b^	DDM	[14]
			1460 µg/disk (conc. 6.8 mg ml^−1^)	^b^	^b^	^b^		
	*Usnea florida*	Acetone	^b^	^b^	^a^	^b^	DDM	[15]
		Chloroform	^b^	^b^	^a^	^b^		
		Methanol	^b^	^b^	^a^	^b^		
Terricolous lichens	*Cetraria aculeata*	Acetone	^b^	^b^	^a^	^b^	DDM	[16]
		Diethyl ether	^b^	^b^	^a^	^b^		
		Ethanol	^b^	^b^	^a^	^b^		
	*Cladonia foliacea*	Methanol	^b^	^b^	^a^	^b^	DDM	[4]
	*Cladonia portentosa*	Acetone	^b^	^b^	^b^	RG%: 72.4	PFT	[5]
	*Cladonia rangiferina*	Ethanol	100	100	16 (20 µl/well)	^b^	BTDM; AWDM	[1]
		Ethyl acetate	150	150	12 (20 µl/well)	^b^		
		Water	150	150	^a^	^b^		
	*Cladonia rangiformis*	Chloroform	^b^	^b^	^a^	^b^	DDM	[17]
		Methanol	^b^	^b^	^a^	^b^		
		Water	^b^	^b^	^a^	^b^		
Saxicolous lichens	*Dermatocarpon miniatum*	Methanol	^b^	^b^	^a^	^b^	DDM	[4]
	*Xanthoparmelia pulla*	Methanol	^b^	^b^	^a^	^b^	DDM	[4]
	*Umbilicaria nylanderiana*	Methanol	^b^	^b^	^a^	^b^	DDM	[9]

Method of measurement abbreviations: *BMMwTTC* Broth microdilution method with 2, 3, 5-triphenyltetrazolium chloride (MIC), *BTDM* Broth tube dilution method (MIC), *DDM* disk diffusion method (IZ, MIC, IR%), *AWDM* Agar well diffusion method (IZ), *PFT* poisoned food technique (IR%; RG%), *IR%* percentage fungus inhibition rate, *RG%* percentage relative growth of mycelium compared to the control sample. For MIC, MFC and IZ abbreviations: see Table 3. Literature abbreviations: [1] Ranković and Mišić (2007); [2] Sarıözlü et al. (2016); [3] Tiwari et al. (2011a); [4] Aslan et al. (2006); [5] Halama and van Haluwin (2004); [6] Karabulut and Ozturk (2015); [7] Babiah et al. (2014a); [8] Shivanna and Garampalli (2015); [9] Gulluce et al. (2006); [10] Anjali et al. (2015a); [11] Babiah et al. (2014b); [12] Tiwari et al. (2011b); [13] Gazo et al. (2019); [14] Türk et al. (2006); [15] Cankılıç et al. (2017); [16] Türk et al. (2003); [17] Yücel et al. (2007)

^a^No effect

^b^Not investigated

The tested extracts were obtained from 28 corticolous, five terricolous and three saxicolous species of lichens. Among the substances extracted from these species, the best MIC value was obtained for the acetone extracts (156.2 × 10^–3^ mg ml^−1^) and the second-best for the methanol extracts (312.5 × 10^–3^ mg ml^−1^) from *Bryoria capillaris*, although the IZ value does not emphasize this potential (IZ: 13–15 mm) (Sarıözlü et al. 2016). Values for the methanol extract from *Flavoparmelia caperata* (MIC: 1.562 mg ml^−1^; IZ: 12.6 mm) and extract based on ethyl acetate from *Parmotrema tinctorum* (MIC: 1.562 mg ml^−1^; IZ: 18.6 mm) (Shivanna and Garampalli 2015) are noteworthy.

Substances extracted with ethanol, ethyl acetate and water from *Alectoria sarmentosa* inhibited the growth of the pathogen at the relatively weakest MIC value, a value which ultimately provided a fungicidal potential (Minimal Fungicidal Concentration: 20 mg ml^−1^—ethanol extract; 50 mg ml^−1^—ethyl acetate and water extracts). The highest effectiveness of the ethanol extract is also confirmed by the IZ value (25 mm), relative to the other combinations (Ranković and Mišić 2007). The acetone extract from *Pseudevernia furfuracea* should be focused on, with a MIC value of 597–640 µg/disk (Türk et al. 2006).

In the case of the percentage inhibition of the *Fusarium solani* mycelium, mixtures of substances extracted with acetone, chloroform and methanol from *Parmotrema reticulatum* (Babiah et al. 2014b) appear to have potential, as does ethanol from *Evernia prunastri* (Karabulut and Ozturk 2015) and acetone extracts from *Hypogymnia physodes* (Halama and van Haluwin 2004).

Among the rarely tested terricolous species, substances extracted from *Cladonia rangiferina* appear to have antifungal potential according to MIC and MFC (Ranković and Mišić 2007).

#### Potential of crude extracts from lichens against *Fusarium udum* (references: Tab. S10)

Only three extracts from the corticolous *Parmotrema reticulatum* were used against *F. udum*. The ED_50_ value indicates that the mixture of substances extracted with ethyl acetate (43.7 µg ml^−1^) and methanol (59.2 µg ml^−1^) has the highest inhibitory potential, and the efficacy of the hexane extract was about four times weaker (Goel et al. 2011b).

### Inhibitory potential of individual lichen secondary compounds against five *Fusarium* species.

#### Potential of lichen secondary metabolites against *F. culmorum* (references: Tab. S11)

Chloroatranorin (MIC: 7.5 mg ml^−1^) and olivetoric acid (MIC: 10 mg ml^−1^) tested against *F. culmorum* showed a weak inhibitory potential (Türk et al. 2006).

#### Potential of lichen secondary metabolites against ***Fusarium fujikuroi*** (references: Table 3)

**Table 3:** Effect of lichen secondary metabolites on *Fusarium fujikuroi*

Biochemical class	Secondary metabolites	Extracting solvents	Results		Measurement method	Literature
			MIC [mg ml^−1^]	IZ [mm]		
Benzyl esters	Barbatolic acid	Methanol	400 × 10^–3^	^b^	MMwR	[1]
Orcinol depsides	Olivetoric acid	Acetone	10 [= 500 µg 50 µl^−1^]	^b^	DDM	[2]
Monocyclic aromatic derivatives	Orsellinic acid	Ethanol–water–hydrogen chloride	15.1 × 10^–3^	^b^	DDM	[3]
Orcinol depsides	Lecanoric acid	Ethanol–water–hydrogen chloride	14.8 × 10^–3^	^b^		[3]
β-Orcinol depsides	Chloroatranorin	Acetone	7.5 [= 300 µg 40 µl^−1^]	^b^	DDM	[2]
	Diffractaic acid	Ethanol–water–hydrogen chloride	16.3 × 10^–3^	^b^	DDM	[3]
	Thamnolic acid	Methanol	^a^	^b^	MMwR	[4]
β-Orcinol depsidones	Norstictic acid	Ethanol–water–hydrogen chloride	16.1 × 10^–3^	^b^	DDM	[3]
	Protocetraric acid		12.6 × 10^–3^	^b^		[3]
Usnic acid derivatives	Usnic acid	Ethanol–water–hydrogen chloride	18.6 × 10^–3^	^b^	DDM	[3]

Method of measurement abbreviations: *DDM* disk diffusion method (MIC), *MMwR* microdilution method with resazurin (MIC). For MIC and IZ abbreviations: see Table 3. Literature abbreviations: [1] Sarıözlü et al. (2016); [2] Türk et al. (2006); [3] Hanuš et al. (2007); [4] Cankılıç et al. (2017)

^a^No effect

^b^Not investigated

Ten lichen secondary metabolites have been tested against *F. fujikuroi* to determine MIC values. Orsellinic, lecanoric, diffractaic, norstictic, protocetraric and usnic acids demonstrated MIC potential below a concentration of 20 × 10^–3^ mg ml^−1^ (Hanuš et al. 2007).

#### Potential of lichen secondary metabolites against *Fusarium oxysporum* (references: Tab. S12)

The antifungal potential of 20 lichen secondary metabolites have been investigated. The MIC value shows that the compound with the highest inhibitory potential is 2-hydroxy-4-methoxy-3,6-dimethylbenzoic acid (MIC: 4 × 10^–3^ mg ml^−1^) supported by the relatively highest IZ value: 26 mm. In addition, the potential of atranorin (MIC: 16 × 10^–3^ mg ml^−1^; IZ: 19 mm) and isomer ( +)-usnic acid (MIC: 16 × 10^–3^ mg ml^−1^; IZ: 13 mm) (Aravind et al. 2014) are notable.

#### Potential of lichen secondary metabolites against *Fusarium solani* (references: Tab. S13)

The antifungal potential of 11 lichen secondary metabolites has been tested. The most fungistatic effect was achieved by barbatolic acid (MIC: 200 × 10^–3^ mg ml^−1^) (Sarıözlü et al. 2016). However, a concentration of 200 × 10^–3^ mg ml^−1^ resulted in 90% of inhibition of mycelial growth dynamics in the experiment with methyl β-orcinol carboxylate (Thadhani et al. 2012).

#### Potential of lichen secondary metabolites against *Fusarium udum* (references: Tab. S14)

The potential of 13 lichen secondary metabolites has been investigated against *F. udum*. According to the percentage inhibition of mycelium growth, the most effective were protolichesterinic acid (IG%: 90.33; ED_50_: 55.68 µg ml^−1^) and atranorin (IG%: 90; ED_50_: 50.32 µg ml^−1^). Isousnic acid (IG%: 81; ED_50_: 70.86 µg ml^−1^) and evernyl (methyl β-orcinol carboxylate) (IG%: 80.46; ED_50_: 82.31 µg ml^−1^) (Goel et al. 2011b) were also highly effective. A comparison of the ED_50_ concentration of sekikaic acid (32.85 µg ml^−1^) (Goel and Singh 2015) shows that it is the most effective, even above that of protolichesteric acid and atranorin.

### Statistics

The MIC value of the extracted substances mixtures from corticolous lichens against *F. oxysporum* (Tab. S16) was statistically significantly lower ($\overline{x }$ = 12.47 mg ml^−1^) compared to the MIC value of terricolous (group no. 1; $\overline{x }$ = 31.96 mg ml^−1^) and saxicolous (group no. 2; $\overline{x }$ = 15.88 mg ml^−1^) species. The confrontation of MIC values of the tested representatives of terricolous and saxicolous lichens turned out to be statistically insignificant (group no. 3).

There were no significant statistical differences between the IZ values of extracts from corticolous ($\overline{x }$ = 14.95 mm) and terricolous ($\overline{x }$ = 12.4 mm) (group no. 1) and saxicolous ($\overline{x }$ = 13.89 mm) (group no. 2) lichen species and between terricolous and saxicolous lichen species (group no. 3) against *F. oxysporum* (see details: Tab. S17).

Against *F. oxysporum* (see: Tab. S18), in all the combinations compared with each other, the MIC value of lichen secondary metabolites ($\overline{x }$ = 1.88 mg ml^−1^) turned out to be statistically significantly lower than the MIC of the extracts from the corticolous (group no. 1), terricolous (group no. 2) and saxicolous (group no. 3) lichen species.

All MIC values of the three non-lichen antifungal substances against *F. oxysporum*, including amphotericin B, ketoconazole, terbinafine, (see: Tab. S19) were found to be statistically significantly lower compared to the extracts from corticolous lichen species (group no. 1), terricolous (group no. 2) and saxicolous (group no. 3) (*vs*. amphotericin B—$\overline{x }$ = 0.017 mg ml^−1^, ketoconazole—$\overline{x }$ = 0.16 mg ml^−1^ and terbinafine—$\overline{x }$ = 0.0115 mg ml^−1^).

The zone of mycelial growth inhibition induced by ketoconazole ($\overline{x }$ = 25.29 mm) was found to be statistically significantly broader in diameter than the IZ of extracts from corticolous (group no. 1), terricolous (group no. 2) and saxicolous (group no. 3) lichen species against *F. oxysporum* (see: Tab. S20).

Against *F. solani* (see: Tab. S21), all four compared non-lichen reference substances (amphotericin B—$\overline{x }$ = 0.01 mg ml^−1^; flucytosine—$\overline{x }$ = 0.41 mg ml^−1^; itraconazole—$\overline{x }$ = 0.037 mg ml^−1^ and voriconazole—$\overline{x }$ = 0.012 mg ml^−1^) were found to have statistically significantly lower MIC values compared to corticolous lichen species ($\overline{x }$ = 14.29 mg ml^−1^).

Against *F. fujikuroi* (see: Tab. S22), the MIC values of amphotericin B ($\overline{x }$ = 0.003 mg ml^−1^), isavuconazole ($\overline{x }$ = 0.005 mg ml^−1^), natamycin ($\overline{x }$ = 0.004 mg ml^−1^), posaconazole ($\overline{x }$ = 0.00065 mg ml^−1^) and voriconazole ($\overline{x }$ = 0.0037 mg ml^−1^) was statistically significantly lower when confronted with MIC values obtained by individual lichen secondary metabolites ($\overline{x }$ = 2 mg ml^−1^). There were no significantly statistical differences between the lichen compounds and fluconazole ($\overline{x }$ = 0.09 mg ml^−1^) and itraconazole ($\overline{x }$ = 0.027 mg^−^ml^−1^).

The MIC values of amphotericin B ($\overline{x }$ = 0.017 mg ml^−1^), ketoconazole ($\overline{x }$ = 0.016 mg ml^−1^), terbinafine ($\overline{x }$ = 0.011 mg ml^−1^) were statistically significantly lower compared to the MIC values of the secondary metabolites against *F. oxysporum* ($\overline{x }$ = 1.88 mg ml^−1^) (see: Tab. S23).

## Discussion

### Assays of crude extracts from various lichen species

The literature data on the effects of the mixture of extracted substances from lichen species indicate their potential to inhibit the mycelial growth of at least eight species of the genus *Fusarium* species (*F. acuminatum*, *F. avenaceum*, *F. culmorum*, *F. fujikuroi*, *F. oxysporum*, *F. roseum*, *F. solani* and *F. udum*) studied. Taking into account the currently known number of representatives of this genus, as well as the number of lichen species investigated by the researchers, the present meta-analysis may constitute a preliminary discernment for in-depth studies on the undertaken subject. Nevertheless, the obtained results of the analysis show several interesting issues.

As the MIC value is an overarching assessment of the antifungal potential of the tested substances relative to the IZ (Furmanek et al. 2019, 2022), the issues described below arise mainly from an analysis of the lowest (methodologically beneficial) inhibitory concentration. Literature data of antifungal non-lichen reference substances show that their antifungal potential is exhibited mostly by MIC: < 1 mg ml^−1^ (Tab. S15A–B). For this reason, attention has been focused on the most competitive and promising combinations of lichen extracts showing concentrations below this MIC value.

#### Fusarium acuminatum

The efficacy of a methanol extract from corticolous *Evernia divaricata* (MIC: 62.5 × 10^–3^ mg ml^−1^) (Aslan et al. 2006) is potentially an extract against *F. acuminatum* at the level of amphotericin B (references: Tab. S15A; cf. Tab. S3 and S15A). Aslan et al. (2006) obtained the extract using a Soxhlet apparatus (10 g of powdered lichen thalli/250 ml of methanol/72 h of extraction process without exceeding the methanol boiling point) (cf. proportions: Table 4); the extract obtained was subjected to filtration and it was concentrated and stored as a dry extract. For MIC determination, the dried mixture of lichen substances was redissolved in 10% dimethylsulfoxide (DMSO), while for IZ, it was redissolved in methanol and filtered for sterilization.

**Table 4:** Proportions of lichen thalli weight and volume of solvent used to prepare the most effective extracts (MIC: < 1 mg ml^−1^; SGI: 100%; ED_50_: < 50 µg ml^−1^) from the various lichen species used against different *Fusarium* species

Lichen species (ecological group)	Dry mass of lichen species (g)	State of lichen species	Solvent volume (ml)	Extracting solvents	Duration of extraction	Extraction method	References
*Alectoria sarmentosa* (corticolous)	40	Powdered	Uncertain: 300	Ethanol, ethyl acetate, water	4 h	Soxhlet apparatus	Ranković and Mišić (2007)
*Cladonia mitis* (terricolous)	0.3	Slightly crushed	100	Acetone	24 h	Left at a room temperature	Tekiela et al. (2021)
*Cladonia rangiferina* (terricolous)	0.3	Slightly crushed	100	Acetone	24 h	Left at a room temperature	Tekiela et al. (2021)
*Cladonia mitis* + *C. rangiferina* (terricolous)	0.3	Slightly crushed	100	Ethanol	24 h	Left at a room temperature	Tekiela et al. (2021)
*Evernia divaricata* (corticolous)	10	Powdered	250	Methanol	72 h	Soxhlet apparatus	Aslan et al. (2006)
*Evernia prunastri* (corticolous)	No data	Powdered	No data	96% ethanol	8 h (at 25 °C)	Soxhlet apparatus	Karabulut and Ozturk (2015)
*Flavoparmelia caperata* (corticolous)	1	Powdered	10	Methanol	24 h	Roratory shaking	Shivanna and Garampalli (2014)
*Hypotrachyna cirrhata* (corticolous)	10	Macerated	20	Water	No data	Maceration	Shahi et al. (2003)
*Leucodermia leucomelos* (corticolous)	10	Macerated	20	Water	No data	Maceration	Shahi et al. (2001)
*Parmotrema austrosinense* (corticolous)	1	Powdered	10	Methanol	24 h	Roratory shaking	Shivanna and Garampalli (2014)
*Parmotrema reticulatum* (corticolous)	45–50	Powdered	500	Ethyl acetate	6 h	Soxhlet apparatus	Goel et al. (2011a, b)
*Physcia aipolia* (corticolous)	1	Powdered	10	Ethyl acetate, methanol	24 h	Roratory shaking	Shivanna and Garampalli (2014)
*Pseudevernia furfuracea* (corticolous)	10	Powdered	100	Acetone	Sonicated—1 h; left overnight	Sonication and left overnight	Türk et al. (2006)
*Roccella montagnei* (corticolous)	1	Powdered	10	Ethyl acetate, methanol	24 h	Roratory shaking	Shivanna and Garampalli (2014)
*Umbilicaria nylanderiana* (saxicolous)	10	Powdered	250	Methanol	72 h	Soxhlet apparatus	Gulluce et al. (2006)

#### Fusarium avenaceum

The antifungal potential of acetone-extracted substances from *Cladonia mitis* and *C. rangiferina* against *F. avenaceum* was tested by Tekiela et al. (2021). They were obtained by 24 h extraction of slightly crushed lichen thallus (0.3 g of dry thalli) in 100 ml of solvent in a plastic container under seal—implicitly, extraction conducted at room temperature (cf. proportions: Table 4). The results show that a 1 ml dose of the extract (although with an unknown total concentration of extracted lichen substances) is a highly effective fungistatic agent, especially in the prospective use of *C. mitis*. A detailed look at the results of mycelial growth dynamics shows that almost half of the biological replicates (14 of 30 ones) in the samples with *C. rangiferina* extract had fungicidal potential or at least provided a strong allelopathic barrier, completely inhibiting *F. avenaceum* mycelial growth what is not obvious from Tekiela et al.’s (2021) experiment. The use of substances from *C. mitis* increased this potential to 70% (21 of 30 biological replicates). Equally effective ethanol mixed extract from *C. mitis* + *C. rangiferina* was found to have an analogous fungicidal potential (14 of 30 biological replicates) as the acetone extract from *C. rangiferina* alone. Because the researchers adopted the PFT method, the results obtained remain incomparable to other ones used to determine the anti-fusarial potential of reference non-lichen substances.

#### Fusarium culmorum

A completely growth-inhibiting extract from *Evernia prunastri* was prepared using ethanol (Table 4) and drying the extract, from which a solution of a particular concentration was obtained for further experiments by redissolving in ethanol (an ambiguous issue based on the description of the extract preparation) (Karabulut and Ozturk 2015). An extract from *Pseudevernia furfuracea* was obtained in acetone (cf. Table 4) using ultrasound (sonification), overnight extraction, and its filtration and evaporation of the solvent (Türk et al. 2006). The dried extract was then re-dissolved in the same solvent to a certain concentration (Yılmaz et al. 2004; Türk et al. 2006), but there is no possibility to equate the results to the potential of non-lichen substances because of the unit is expressed in a different way.

#### Fusarium oxysporum

Among the corticolous lichens, the fungicidal potential of acetone and ethyl acetate extracts from *Alectoria sarmentosa* was achieved by extracting an uncertain proportion of lichen thalli and solvent (see Table 4) using a Soxhlet apparatus and dried extract (Ranković and Mišić 2007). The MIC potential of the reference substances (Tab. S15A) is better than the extract from *A. sarmentosa*, but the MFC potential may prove to be effective because of lacking MFC potential of the reference substances against *F. oxysporum*. The achieved IZ value (15 and 18 mm) indicates that this species may be a source of antibiotic substances partly competing with bavistin and ketoconazole (cf. Tab. S6 and Tab. S15A).

The methanol extract from *Flavoparmelia caperata* (MIC: 0.097 mg ml^−1^) was prepared by Shivanna and Garampalli (2014) by shaking (cf. proportions: Table 4) a thallus in a solvent, filtration, its evaporation and re-dissolving the dry extract. Based on the MIC, this extract indicates an even stronger fungistatic potential than flucytosine and fluconazole. Additionally, it appears to be slightly weaker than ketoconazole and miconazole, but amphotericin B, bavistin, itraconazole, natamycin, posaconazole, ravuconazole, terbinafine and voriconazole are more effective (cf. Tab. S6 and Tab. S15A). It should be noted that the efficacy of the different *F. caperata* extracts were within a rather wide range of MIC values, which may be due not only to different methodological assumptions of the experiments (see details and references: Tab. S6), including the extraction process (Zizovic et al. 2012; Parrot et al. 2015), but different concentrations of lichen substances in the lichen thalli depending on biotic (Badridze et al. 2019; Farkas et al. 2020; Norouzi et al. 2020; Prokopiev et al. 2017) and abiotic (Armaleo et al. 2008; Cansaran Duman et al. 2008; Nybakken and Julkunen-Tiitto 2006; Smeds and Kytöviita 2010) factors. However, the described example shows that more in-depth research is needed to determine more precisely the most optimal method for extracting a mixture of substances from this corticolous species to achieve a relatively high antifungal potential.

In an experiment by Shivanna and Garampalli (2014), methanol-extracted substances from *Parmotrema austrosinense* (MIC: 0.39 mg ml^−1^; IZ: 21.3 mm) and ethyl acetate and methanol extracts from *Physcia aipolia* (MIC: 0.39 mg ml^−1^; IZ: 12.3–12.6 mm) and *Roccella montagnei* (MIC: 0.39 mg ml^−1^; IZ: 11.3–13 mm) were tested. Based on the available data, all these extracts appear to be more effective than flucytosine but weaker than the others (amphotericin B, bavistin, fluconazole, itraconazole, ketoconazole, miconazole, natamycin, posaconazole, ravuconazole, terbinafine and voriconazole) (cf. Tab. S15A). From an IZ point of view, they could also compete with bavistin and ketoconazole, but due to their weaker MIC potential, conclusions are unwarranted. Nonetheless, for lichen extracts with relatively high IZ values (acetone, ethanol, ethyl acetate and methanol extracts from *Cetrelia braunsiana*, acetone and methanol extracts from *Parmotrema perlatum* and ethanol extract from *R. montagnei*), due to the lack of MIC data, further research on the potential of these extracts is needed (see IZ details and references: Tab. S6).

The effectiveness of acetone-extracted substances from *Pseudevernia furfuracea* (Tab. S6) is repeated (Türk et al. 2006), as in their use against *Fusarium culmorum* (cf. Tab. S4 and S6).

The fungicidal potential against *F. oxysporum* is possessed by water-extracted substances from *Hypotrachyna cirrhata* (Shahi et al. 2003) and *Leucodermia leucomelos* (Shahi et al. 2001), which were obtained by maceration of the lichen thallus in water (cf. proportions: Table 4) and filtration of the obtained extract. The demonstrated fungicidal effects (100% according to SGI) emphasize the necessity of further research on substances from these two lichen species.

An ethyl acetate extract from *Parmotrema reticulatum* is of interest, with the highest dose of ED_50_ (43.7 µg ml^−1^). The weakest effect was obtained by a mixture of substances extracted with water, and although re-dissolved in acetone (Goel et al. 2011a) shows the possibility of its use as a safe antifungal agent. An analogous conclusion was drawn from the use of substances extracted from the epigeic *Stereocaulon himalayense* (Goel et al. 2011a). Among epigeic species, the fungicidal potential (MFC) of the ethanol and ethyl acetate extracts from *Cladonia rangiferina* (Ranković and Mišić 2007) and, similarly to that against *F. avenaceum*, the acetone extract of the same lichen species and *C. mitis* in the study of Tekiela et al. (2021) are comparable (Tab. S7). A methanol extract from epilithic *Umbilicaria nylanderiana* (MIC: 62.5 × 10^–3^ mg ml^−^1; IZ: 33 mm) (Gulluce et al. 2006) is the only one of the tested lichen species of this ecological group (Tab. S8) that can compete with flucytosine and fluconazole; a weaker advantage is maintained over miconazole, while this value partly overlaps with the MIC potential exhibited by amphotericin B and ketoconazole. The potential of bavistin, itraconazole, natamycin, posaconazole, ravuconazole, terbinafine and voriconazole is still more effective (cf. Tab. S8 and Tab. S15A). This extract was obtained by extraction of the lichen thallus (cf. proportions: Table 4) in a Soxhlet apparatus at a temperature not exceeding the boiling point of methanol, followed by filtration and evaporation of the solvent. For the determination of MIC, the dry extract obtained was re-dissolved in 10% DMSO, whereas for IZ, the dry extract was re-dissolved in methanol to a particular concentration and sterilized by filtration.

Interestingly, theoretically, all combinations of extracts against *F. oxysporum* that showed a positive result of the inhibition zone (IZ) could have antifungal potential at the ketoconazole level range. To a lesser extent, the IZ value ≤ 11 mm exhibited by different combinations of lichen extracts may indicate an alternative to the systemic fungicide bavistin (cf. Tab. S6, S7, S8 and Tab. S15A).

The present analysis provides preliminary evidence in the literature for statistical differences in the potential of extracts based on lichen species from different ecological groups (Tab. S16). Extrapolation of these preliminary results requires further experiments due to limited representatives of tested terricolous and saxicolous lichens. Obtained results show, however, that MIC values for extracts from corticolous lichens had significantly stronger fungistatic potential against *F. oxysporum* in a comparison to both other groups, while IZ values does not show any significant relationship between the three ecological groups (Tab. S17). However, the results of lichen extracted substances against *F. oxysporum* described above indirectly show the necessity to increase the contribution of, at least more easily available, epigeic macro-lichen species as new sources of lichen substances.

#### Fusarium roseum

Lichen extracts from five corticolous species have been tested against *F. roseum* to investigate their IZ potential. This fungal species remains untested against reference substances, with one exception, the lack of fungistatic effectivity (IZ) of ketoconazole. On this basis, it can only be concluded that all extracts tested so far, which showed a positive effect, especially the methanol extract from *Parmotrema thomsonii* (IZ: c. 25 mm), are theoretically fungistatic agents at the potential level of this antibiotic substance so that further research in this direction is required (see Tab. S9; cf. Tab. S9 and S15B).

#### Fusarium udum

Only the fungistatic potential of three extracts from corticolous *Parmotrema reticulatum* was tested against *F. udum*, for which the extract of ethyl acetate (ED_50_: 43.7 µg ml^−1^) shows a higher potential (Goel et al. 2011b) (extraction process: Goel et al. 2011a). The only comparison to the reference substance potential is possible for hexaconazole (ED_50_: 22.01 µg ml^−1^) (Goel et al. 2011a), showing a weaker but potentially replaceable antibiotic by a mixture of substances from this lichen species (cf. Tab. S10 and Tab. S15B).

### Inhibitory potential of crude extracts from lichen species – implications

The greatest interest has been focused on natural antifungal agents against *F. oxysporum*.

This review mentions 14 lichen species from which the obtained mixtures of lichen substances seem to have the highest inhibitory potential (Table 4), which may replace in future some of the antibiotic reference substances. This category includes mainly corticolous species (*Alectoria sarmentosa*, *Evernia divaricata*, *E. prunastri*, *Flavoparmelia caperata*, *Hypotrachyna cirrhata*, *Leucodermia leucomelos*, *Parmotrema austrosinense*, *P. reticulatum*, *Physcia aipolia*, *Pseudevernia furfuracea* and *Ramalina montagnei*), two terricolous species (*Cladonia mitis* and *C. rangiferina*) and one saxicolous species (*Umbilicaria nylanderiana*). Phenomena shown by the most frequently tested corticolous species seem to be linked to their easier accessibility to terricolous and saxicolous ones. However, the significantly higher MIC potential of lichen extracts from corticolous species against *F. oxysporum* (Tab. S16) indicates that this may also be due to the qualitative-quantitative relationship of extracted lichen substances (Furmanek et al. 2022) present in the dissolved form in the extract or in the form of dry extract. However, the review carried out herewith does not answer the question of whether these differences could be due to evolutionary and co-evolutionary relationships as interactions between dissolved lichen substances and a pathogen/saprotroph such as the species *F. oxysporum* under natural environmental conditions.

The potential of the extract in the light of the sum of interactions of lichen substances demonstrates the wide range of MIC values exhibited by various combinations of lichen extracts against *Fusarium* species (Tables 1 and 2; Tab. S3–S10). The obtained lichen extract may consist of different proportions of primary substances, such as polysaccharides (e.g., Cordeiro et al. 2003, 2011; Badridze et al. 2019; Freysdottir et al. 2008) or carotenes (e.g., Czeczuga and Lallemant 1993; Czeczuga and Christensen 1994), as well as from secondary metabolites of lichens (Farkas et al. 2020; Prokopiev et al. 2017; Prokop'ev et al. 2018; Latkowska et al. 2019), including total phenolic compounds (Badridze et al. 2019; Zagoskina et al. 2013; Zavarzina et al. 2019). In addition, such mixtures seem to be complemented by low concentrations of lichen mycotoxins (Burkin and Kononenko 2014, 2015a, 2015b); their potential depends upon their mutual concentration (Ráduly et al. 2020) as for the mycotoxins that are present in lichen thalli (aflatoxin B1, sterigmatocystin or ochratoxin A) (Burkin and Kononenko 2015b). Most mycotoxins are highly soluble in organic solvents (Bennett and Klich 2003). Interestingly, it is conceivable that in virtually every case, the potential of the extract may be affected by the presence of usnic acid (Cansaran Duman et al. 2008; Burkin et al. 2013). Determination of the anti-fusarial potential based on the interactions occurring in the extract remains in the realm of future experiments.

Regardless of the interaction between the extracted substances, a comparative listing of the elements comprising the extraction procedure of the most effective lichen extracts (Table 4) shows that their extraction proved not to be completely methodologically dependent, which may indirectly indicate the influence of the interaction between the substances. The preparation of all the extracts described in Table 4 emphasises the aim of new antifungal agents, limiting the ecological point of view (although a small exception by the work of Tekiela et al. (2021), due to the use of lichen thalli in unpowdered form and the method of measuring mycelial growth—PFT; see Tab. S4). The data show that a small weight of lichen thallus can be sufficient to obtain effective natural antifungal agents. The volume of the solvent does not have any particular relationship to the weight of lichen thalli, although it does have an influence on the extraction process through diffusion and the experimental methodology used, such as the dosage of substances present in the extract. Mostly organic solvents (acetone, ethanol, ethyl acetate and methanol) have been used to obtain the most effective mixtures of lichen substances, although the crystallization of the substances and their re-dissolution in DMSO appear to influence their fungistatic potential, assuming the interactions between them (Furmanek et al. 2022). Most interesting is the use of water and the process of maceration of the lichen thallus of *Hypotrachyna cirrhata* and *Leucodermia leucomelos* at room temperature, indicating the underestimation of water as an effective solvent. Moreover, if one combines, as shown above, the antifungal effects with the extraction of usnic acid in the maceration process, even using ethanol, the extracted volume of this metabolite is rather relatively low (Zizovic et al. 2012), which indicates that the represented fungicidal potential may be determined by the efficiency of the water extract as in the experiments of Shahi et al. (2001, 2003). The duration of the extraction process may not prove to be directly related to the potential of the extracts, at least for those extractions using organic solvents and room temperature.

A unique experimental methodology was adopted by Tekiela et al. (2021) by extracting lichen substances from thalli of mixed lichen species, demonstrating that the mixing of two epigeic species, *Cladonia mitis* and *C. rangiferina*, may provide a good concept for their use against the pathogens of *Fusarium* spp. fungi. The methodology adopted for this experiment illustrates the changes in mycelial growth dynamics over time, which is lacking in other experiments using the PFT method (Goel et al. 2011a, b; Goel and Singh 2015; Karabulut and Ozturk 2015; Thadhani et al. 2012; Vinayaka et al. 2014). Repeating these combinations using water may prove to be promising in the phytopharmacological field.

Comparison of the MIC potential of the most effective lichen extracts indicates possible substitution of flucytosine and, to a lesser extent, amphotericin B, bavistin, miconazole, fluconazole and ketoconazole against *F. acuminatum* and *F. oxysporum*. Against the most commonly tested *F. oxysporum*, most reference substances showed a stronger potential compared to the obtained lichen extracts (Table 6).

### Assays of lichen secondary metabolites against *Fusarium* species

#### Fusarium culmorum

Although the two secondary compounds tested (chloroatranorin and olivetoric acid) exerted a much weaker inhibitory effect compared to the MIC potential of amphotericin B, caspofungin, posaconazole, tebuconazole and voriconazole, due to data scarcity indicating lack of efficacy of fluconazole, itraconazole and ketoconazole, these metabolites could theoretically prove to be competitive against them (Tab. S11; cf. Tab. S11 and S15A).

#### Fusarium fujikuroi

The MIC potential of the secondary metabolites (orsellinic, lecanoric, diffractaic, norstictic, protocetraric and usnic acids) of < 20 × 10^–3^ mg ml^−1^ can compete with flucytosine (including barbatolic acid; MIC: 400 × 10^–3^ mg ml^−1^) and fluconazole; a smaller advantage is maintained over the potential of clotrimazole, itraconazole, ketoconazole, miconazole, while there is little overlap with the potential shown by amphotericin B, isavuconazole, posaconazole, ravuconazole, terbinafine and voriconazole. The efficacy of difenoconazole (agricultural fungicide), micafungin, natamycin, propiconazole and tebuconazole is better than the inhibitory potential of lichen compounds investigated (Table 3; cf. Tab. 3 and S15A).

#### Fusarium oxysporum

The most effective of the tested secondary metabolites, 2-hydroxy-4-methoxy-3,6-dimethylbenzoic acid (MIC: 4 × 10^–3^ mg ml^−1^; IZ: 26 mm), clearly prevails over the MIC potential of flucytosine, fluconazole and miconazole. Its potential is also more effective than bavistin (both in terms of MIC and IZ) and is identical to the MIC of natamycin. The compilation shows that this compound can compete strongly with ketoconazole, also from the point of the IZ view, partly competing with amphotericin B, itraconazole, posaconazole, ravuconazole, terbinafine and voriconazole. Consequently, 2-hydroxy-4-methoxy-3,6-dimethylbenzoic acid has the potential to replace all described non-lichen substances against *F. oxysporum* (cf. Tab. S12 and Tab. S15A).

Atranorin (MIC: 16 × 10^–3^ mg ml^−1^; IZ: 19 mm) and ( +)-usnic acid (MIC: 16 × 10^–3^ mg ml^−1^; IZ: 13 mm) possess a slightly weaker potential. Both secondary metabolites are competitive with all the reference substances, in terms of 2-hydroxy-4-methoxy-3,6-dimethylbenzoic acid potential, except natamycin (based on a conclusion derived from only one literature source) (cf. Tab. S12 and Tab. S15A). Against flucytosine and fluconazole, lecanoric acid (MIC: 0.125 mg ml^−1^) appears to have a higher potential, whereas compared to flucytosine all secondary compounds reaching MIC values of 0.25 mg ml^−1^ (2′-O-methylanziaic, physodic, fumarprotocetraric, gyrophoric and usnic acids) and 0.5 mg ml^−1^ (methyl evernate, atranorin and protocetraric acid) appear to be more effective (cf. Tab. S12 and Tab. S15A).

#### Fusarium solani

Based on literature data, barbatolic acid (200 × 10^–3^ mg ml^−1^) has the highest MIC against *F. solani*, showing higher efficacy than flucytosine and slightly weaker efficacy than fluconazole. The other antifungal substances (amphotericin B, bavistin, caspofungin, isavuconazole, itraconazole, ketoconazole, miconazole, natamycin, posaconazole, propiconazole, rifampin, terbinafine, tebuconazole and voriconazole) have significantly greater efficacy (cf. Tab. S13 and Tab. S15B). It is not possible to compare the potential of methyl orsellinate, methyl β-orcinol carboxylate and lecanoric acid in relation to the fungistatic efficacy of reference substances, but due to the fact that these lichen secondary compounds show a percentage of high inhibitory potential, further studies using them are necessary.

#### Fusarium udum

The potential of hexaconazole (ED_50_: 22.01 µg ml^−1^) has proved to be higher than all secondary metabolites tested against *F. udum*. However, the potential of protolichesterinic and sekikaic acids and atranorin, methyl haematommate and isousnic acid is only slightly weaker (cf. Tab. S14 and Tab. S15B).

### Inhibitory potential of secondary metabolites from lichen species – implications

The a priori assumed antagonistic interactions between substances extracted from the thalli of various lichen species against *Fusarium* species should be confirmed considering the higher antifungal efficacy of lichen secondary metabolites. A preliminary assessment is provided by comparing the abundance of the most effective lichen extracts and secondary compounds (cf. Tables 5 and 6). The higher fungistatic potential of the secondary lichen metabolites ($\overline{x }$ = 1.88 mg ml^−1^), although limited to *F. oxysporum*, is confirmed by the MIC value as being statistically significantly lower compared to extracts from the epiphytic ($\overline{x }$ = 12.47 mg ml^−1^), epigeic ($\overline{x }$ = 31.96 mg ml^−1^) and epilithic ($\overline{x }$ = 15.88 mg ml^−1^) lichen species (Tab. S18). Falsification of these preliminary meta-analysis results will require further experimentation and standardization of research methodologies.

**Table 5:** Fungistatic potential of the lichen extracts with the highest level of efficacy against the tested *Fusarium* species in comparison with the MIC and, if stated otherwise, IZ potential of the non-lichen substances

*Fusarium* species	Lichen extracts		Reference substances		
	Lichen species	Extracting solvent	Lower potential compared to extract	Overlapping potential compared to extract	Higher potential compared to extract
*Fusarium acuminatum*	*Evernia divaricata*	Methanol	^a^	Amphotericin B	^a^
*Fusarium oxysporum*	*Alectoria sarmentosa*	Acetone, ethyl acetate	^a^	Ketoconazole (IZ), bavistin (IZ)	Amphotericin B, bavistin, flucytosine, fluconazole, itraconazole, ketoconazole, miconazole, natamycin, posaconazole, ravuconazole, terbinafine, voriconazole
	*Flavoparmelia caperata*	Methanol	Flucytosine, fluconazole	^a^	Amphotericin B, bavistin, itraconazole, ketoconazole, miconazole, natamycin, posaconazole, ravuconazole, terbinafine, voriconazole
	*Parmotrema austrosinense*	Methanol	Flucytosine	Bavistin, ketoconazole	Amphotericin B, fluconazole, itraconazole, miconazole, natamycin, posaconazole, ravuconazole, terbinafine, voriconazole
	*Physcia aipolia*	Ethyl acetate, methanol			
	*Roccella montagnei*	Ethyl acetate, methanol			
	*Umbilicaria nylanderiana*	Methanol	Flucytosine, miconazole	Amphotericin B, ketoconazole	Bavistin, itraconazole, natamycin, posaconazole, ravuconazole, terbinafine, voriconazole

See details and references in Tab. S3, S6 and S8

^a^No data

**Table 6:** Fungistatic potential of secondary metabolites of lichens against the tested *Fusarium* species in confrontation with the MIC and, if indicated otherwise, the IZ potentials of non-lichen substances

*Fusarium* species	Lichen secondary metabolites	Reference substances		
		Lower potential compared to metabolite	Overlapping potential compared to metabolite	Higher potential compared to metabolite
*Fusarium culmorum*	Chloroatranorin	Fluconazole Itraconazole Ketoconazole	^a^	Amphotericin B Caspofungin Posaconazole Tebuconazole Voriconazole
	Olivetoric acid			
*Fusarium fujikuroi*	Orsellinic acid Lecanoric acid Diffractaic acid Norstictic acid Protocetraric acid Usnic acid	Flucytosine Fluconazole Clotrimazole Itraconazole Ketoconazole Miconazole	Amphotericin B Isavuconazole Posaconazole Ravuconazole Terbinafine Voriconazole	Difenoconazole Micafungin Natamycin Propiconazole Tebuconazole
	Barbatolic acid	Flucytosine	^a^	Amphotericin B Clotrimazole Difenoconazole Fluconazole Isavuconazole Itraconazole Ketoconazole Micafungin Miconazole Natamycin Posaconazole Propiconazole Ravuconazole Tebuconazole Terbinafine Voriconazole
*Fusarium oxysporum*	2-Hydroxy-4-methoxy-3,6-dimethylbenzoic acid	Bavistin (MIC and IZ) Flucytosine Fluconazole Miconazole	Natamycin Ketoconazole (MIC and IZ) Amphotericin B Itraconazole Posaconazole Ravuconazole Terbinafine Voriconazole	^a^
	Atranorin (+)-Usnic acid	^a^	Amphotericin B Bavistin (MIC and IZ) Flucytosine Fluconazole Ketoconazole (MIC and IZ) Miconazole Posaconazole Ravuconazole Terbinafine Itraconazole Voriconazole	Natamycin
	Lecanoric acid	Flucytosine Fluconazole	^a^	Amphotericin B Bavistin Ketoconazole Miconazole Natamycin Posaconazole Ravuconazole Terbinafine Itraconazole Voriconazole
	2′-O-methylanziaic acid Atranorin Fumarprotocetraric acid Gyrophoric acid Methyl evernate Physodalic acid Protocetraric acid Usnic acid	Flucytosine	^a^	Amphotericin B Bavistin Fluconazole Ketoconazole Miconazole Natamycin Posaconazole Ravuconazole Terbinafine Itraconazole Voriconazole
*Fusarium solani*	Barbatolic acid	Flucytosine	^a^	Amphotericin B Bavistin Caspofungin Fluconazole Isavuconazole Itraconazole Ketoconazole Miconazole Natamycin Posaconazole Propiconazole Rifampin Terbinafine Tebuconazole Voriconazole

See details and references in adequate tables: Table 3 and Tab. S11, S12 and S13

^a^No data

This finding proves to be further supported by the results of the statistical analysis of the higher antibiotic potential when confronted with lichen extracts against *F. oxysporum* and *F. solani* and when compared to the potential of lichen secondary compounds against *F. fujikuroi* and *F. oxysporum* (cf. Tab. S19, S20 and S21, and Tab. S22 and S23), where for certain comparative combinations, the potential of the reference substances did not prove to be statistically significantly different against the group of tested secondary metabolites (Tab. S22). Probably, the small number of groups influenced the obtained result of the statistical analysis. On the other hand, the antifungal potential of some of the antibiotics listed in Tab. S15A–B (e.g., flucytosine and fluconazole) may turn out to be weaker with respect to the lichen secondary metabolites, but the limited amount of data only allows tentative conclusions to be drawn.

However, in general, the present data review and statistical analysis indicate antagonism among extracted substances included in lichen extracts. The analysis of data does not directly indicate synergistic or at least additive (in terms of inhibitory potential) interactions of lichen substances, although they seem likely to occur in mixtures of water-extracted substances from *Hypotrachyna cirrhata* (Shahi et al. 2003) and *Leucodermia leucomelos* (Shahi et al. 2001). The use of water as a solvent indicates the implications in terms of obtaining rather different qualitative and quantitative compositions of the extracted substances relative to organic solvents, which may have implications for the direction (inhibition or stimulation) and dynamics of mycelial growth (viewed from a species-specific perspective). The limited number of experiments using water extracts and the research methodology used (e.g., few data using PFTs) suggest a wider consideration of other research methods in future experiments.

The data presented in Table 6 indicate the potential for the use of several lichen secondary metabolites in the substitution of non-lichen antifungal substances. The reference antibiotics that can be replaced include the most frequently flucytosine or fluconazole and ketoconazole. Many of the non-lichen antifungal substances achieve comparable potential against lichen secondary metabolites. Interestingly, 2-hydroxy-4-methoxy-3,6-dimethylbenzoic acid used against *F. oxysporum* was found to be at least equally effective of all 12 reference substances, but this needs further investigation. Indirectly, the different potential of the lichen secondary compounds in relation to the reference substances against *Fusarium* species seems to show this potential from the point of view of resistance of the specific fungus species, irrespective of the results obtained with the different methodology (Table 6).

### The influence of secondary compounds on inhibitory effect of lichen extracts—predictions

In understanding the antifungal potential of the most effective lichen extracts resulting from the sum of the interactions of the substances present in them, an analysis of the most effective individual secondary metabolites used against a given fungal species should be helpful. However, in the case of *Evernia divaricata* extract used against *F. acuminatum*, no data are available on the potential of individual lichen compounds such as divaricatic and evernic acids. Usnic acid, as well as atranorin and fumarprotocetric acid, present in extracts from *Cladonia mitis* and *C. rangiferina*, respectively (Tekiela et al. 2021), may have an impact on limiting the growth dynamics of *F. avenaceum* mycelium (not yet subjected to experiments using secondary compounds). All these metabolites exerted measurable inhibition against *F. oxysporum*. Given the potential of the extract of mixed thalli of these two species, combining their metabolites in this way may prove to be promising, also in terms of the potential of isolated and mixed metabolites such as atranorin + usnic acid + fumarprotocetric acid (cf. Tab. S4 and Table 7).

**Table 7:** Chemical composition of the main secondary metabolites of lichens present in the thallus of species with distinctive anti-fusarial potential of the extracts obtained from these ones

*Fusarium* species	Lichen species	Chemical profile of secondary metabolites of lichen species in data literature	References for chemical profile of secondary metabolites
*Fusarium acuminatum*	*Evernia divaricata*	Divaricatic acid, evernic acid	Çobanoğlu et al. (2010)
		Several terpenes derivatives (essential oils)	(detailed composition: Kahriman et al. 2011)
*Fusarium avenaceum*	*Cladonia mitis*	Usnic acid	Tekiela et al. (2021)
		Usnic acid, ± fumarprotocetraric acid, ± rangiformic acid	Smith et al. (2009)
	*Cladonia rangiferina*	Atranorin, fumarprotocetraric acid	Tekiela et al. (2021)
*Fusarium culmorum*	*Evernia prunastri*	Atranorin, evernic acid, usnic acid	Smith et al. (2009)
		Several terpenes derivatives (essential oils)	(detailed composition: Kahriman et al. 2011)
	*Pseudevernia furfuracea*	Atranorin, physodic acid, olivetoric acid	Smith et al. (2009)
		Atranorin, chloroatranorin, physodic acid, olivetoric acid	Türk et al. (2006)
*Fusarium oxysporum*	*Flavoparmelia caperata*	Caperatic acid, protocetraric acid, usnic acid	Smith et al. (2009)
	*Hypotrachyna cirrhata*	Atranorin, chloroatranorin, consalazinic acid, galbinic acid, protocetraric acid, salazinic acid	Furmanek et al. (2022); Nash et al. (2002)
	*Leucodermia leucomelos*	Salazinic acid, zeorin	Furmanek et al. (2022); Smith et al. (2009)
	*Parmotrema austrosinense*	Atranorin, dihydrolichesterinic acid, chloroatranorin, cladonioidesin, hydroxybenzoic acid, hypoconstictic acid, lecanoric acid, lichesterinic acid/protolichesterinic acid, 19-ethylprotolichesterinic acid, linoleic acid, linoleic acid isomer, methyl β-orsellinate, orsellinic acid	Kumar et al. (2018)
	*Parmotrema reticulatum*	atranorin, chloroatranorin, salazinic acid (major), consalazinic acid (minor)	Smith et al. (2009)
	*Physcia aipolia*	atranorin, zeorin	Smith et al. (2009)
	*Roccella montagnei*	Atranol, divarinol, divarinolmonomethylether, ethyl divaricatinate, ethyl haematommate, ethyl orsellinate, haematommic acid, methyl-2,6-dihydroxy-4-methylbenzoate, orcinol	Tatipamula et al. (2019)
		everninic acid, roccellic acid	Mishra et al. (2017)
		Angardianic acid, erythrin, lecanoric acid, montagnetol, orsellinylmontagnetol A or B or C, orsellinylmontagnetol D, roccellaric acid, roccellic acid, 8-methoxytrypethelone methylether or bis-(2,4-dihydroxy-6-n-propylphenyl)-methane or pannaric acid	Ferron et al. (2020)
	*Umbilicaria nylanderiana*	Gyrophoric acid	Smith et al. (2009)
*Fusarium udum*	*Parmotrema reticulatum*	See: as above	Smith et al. (2009)

It is not possible to determine which metabolites of *Evernia prunastri* could have strongly inhibited the growth of *F. culmorum*. It is also difficult to determine whether the presence of chloroatranorin and olivetoric acid could have played a greater role in the growth inhibition of this species. Their individual potential does not seem to indicate this (Tab. S11); in this case, the influence of atranorin plus additional interactions with other substances should be considered (cf. Tab. S5, S11 and Table 7). The potency of growth inhibition of *F. oxysporum* by *Flavoparmelia caperata* extract may have been due to the presence of protocetraric and usnic acids; the effect of caperatic acid remains undetermined (cf. Tab. S6, S12 and Table 7).

In the context of the fungicidal efficacy of the *Hypotrachyna cirrhata* extract, the potential of single secondary metabolites appears to be weakened. Atranorin appears to be of the greatest importance alongside the weaker protocetraric acid. Chloroatranorin and salazinic acid are unlikely to have contributed to the specific efficacy of this extract, although the latter in combination with zeorin and other lichen substances appear to be important for the fungicidal potential of an extract from *Leucodermia leucomelos*. Determination of the effect resulting from the combination of chloroatranorin and salazinic acid should help to indicate the reason for the inhibitory strength of the two water extracts discussed (cf. Tab. S6, S12 and Table 7).

Considering many secondary metabolites present in the *Parmotrema austrosinense* extract, its relatively strong potential could be due to atranorin, and perhaps lecanoric acid, and assumed influence of fatty acids such as lichesterinic and protolichesterinic acids (Tab. S2), which may have played a role, leading to the dysfunction of the cell membrane (other fatty acids: Avis and Bélanger 2001; Bhattacharyya et al. 2020). The latter strongly reduced the growth of *F. udum* species, although analogous data against *F. oxysporum* are lacking (cf. Tab. S6, S12 and Table 7). The inhibitory efficiency of an extract from *Parmotrema reticulatum* seems to be another example where atranorin may play a major role, although due to the presence of salazinic acid as the dominant compound, an experiment using these two metabolites proved to have potential. A similar conclusion can be drawn from an extract from *Physcia aipolia*, in which atranorin and zeorin are found, indicating further experiments involving these two combined compounds (cf. Tab. S6, S12 and Table 7).

In the light of the potential of the many metabolites that could be included in the extract of *Roccella montagnei*, lecanoric acid may have influenced an effect (cf. Tab S6, S12 and Table 7). In the case of the *Umbilicaria nylanderiana* extract, a significant contribution to its inhibitory potential may be related to the presence of gyrophoric acid, although the extract itself showed a stronger fungistatic potential (cf. Tab. S8, S12 and Table 7). The potential, as in the case of *Parmotrema reticulatum* extract against *F. oxysporum*, and its effectiveness against *F. udum* may be related to the presence of mainly atranorin and salazinic acid (cf. Tab. S6, S14 and Table 7).

### Extracts *vs* secondary metabolites—application

In absolute terms, the anti-fusarial potential of the isolated secondary metabolites is stronger than the mycelial growth inhibitory potential exerted by the mixture of substances included in the lichen extracts, apart from the few exceptions.

Utilization of single secondary metabolites should have been directed mainly to medical applications. Their contact with the afflicted area (e.g., by oral application, oils, tablets and drops) with their high potency to inhibit fungal growth and thus indirectly limit the biosynthesis of their mycotoxins, may be an effective means to alleviate the course of a disease. In this light, they could potentially replace currently used antifungal antibiotics such as flucytosine, fluconazole, itraconazole or ketoconazole and others (Table 6). Although evidence is lacking, it seems that their advantage may be their short duration of biological activity in the body. In order to properly assess their safety and usefulness, experiments on their pharmacokinetics in animals and man would have to be performed.

There is evidence that usnic acid can synergistically support the antimicrobial potential of some antibiotics (Segatore et al. 2012). In the context of antifungal activity, direct data are lacking, but at the current stage of knowledge, an attempt to link lichen secondary metabolites in interaction with applied antibiotic agents seems promising.

It also appears that compounds that are highly soluble in fat, e.g., protolichesterinic acid, could be used as potential food preservatives, thereby extending the expiry date, for example, meat.

The weaker antifungal potential of lichen extracts may paradoxically be beneficial from the phytopharmacological point of view, especially with the use of water as solvent. Their application as potential fungistatic agents for use in agriculture or forestry is not only dependent on the potency of inhibition of spore germination and mycelial growth, but primarily on the long-term duration of their biological activity, especially during exposure to external factors. A mixture of lichen substances (as an extract) seems to be more resistant in the context of maintaining the chemical structure of the constituent substances compared to single lichen metabolites. This is influenced by abiotic factors like UV-A/B radiation causing the breakdown of the chemical structure, i.e., compounds with relatively strong inhibitory potential, atranorin (Begora and Fahselt 2001) or the solvent used (Vos et al. 2018), and biotic factors, such as the breakdown of usnic acid by fungi (Bandoni and Towers 1967). The different fungistatic potential of the extracts may be influenced by the substrate from which the lichen species were collected (Badridze et al. 2019; in vitro studies of substrate modifications: e.g., Elshobary et al. 2016), including atranorin (Latkowska et al. 2019). This implies the planning of experiments, aiming to obtain the highest possible concentrations of the desired lichen substances, if only from the point of view of the concentration of the extract with usnic acid (Ivanovic et al. 2013; Zizovic et al. 2012) or the relative proportion of polysaccharides in it (Ullah et al. 2019), providing a background for the selective efficiency of extracts against the growth dynamics of different fungal species (not only against *Fusarium* spp.). On the other hand, the breakdown of the chemical structure of substances and their metabolism by other organisms is desirable in the light of their ecological safety. If the long-term fungistatic effects in the field for more than a week, as in the in vitro study by Tekiela et al. (2021), could be confirmed (Tab. S4 and Tab. S7), it would indicate the use of lichen extracts as plant protection agents in future, especially as extracts based on mixtures of lichen thalli from various species.

Notwithstanding the above, this review indicates that both lichen extracts and their individual secondary metabolites may constitute a source of natural antibiotic substances with potential for use in medicine, agronomy, phytopharmacology and forestry, especially in the light of increasing microbial resistance to the currently substances used.

## Conclusions

This meta-analysis of data on the inhibitory potential of lichen extracts and lichen secondary metabolites against *Fusarium* species shows that they will be increasingly important as fungistatic agents in future.

The abundance of lichens subjected to experiments seems to be high, but they represent only a small portion of the currently known species. *Fusarium oxysporum* is so far the most frequently tested species of this genus. Less attention has been paid to the possibility of preventing the growth of *F. solani*. The other *Fusarium* species mentioned in this paper represent only preliminary experiments, but the results of some methodological combinations appear to be promising.

In general, the heterogeneity of the research methodologies used can be seen. While the variety of methods used and forms of presentation of results is acceptable, reporting IZ results without testing the MIC may only encourage deeper research to determine this. Conversely, specifying IZ alongside MIC may confirm the efficacy of a particular methodological combination due to a slightly different interpretation of the results obtained (Furmanek et al. 2019). It is also apparent that the PFT method is underestimated, despite the fact that due to this, it is possible to show relative changes (compared to control samples) in mycelial growth dynamics over time (see: Tekiela et al. 2021); this possibility is not left to other methods such as BTDM, BMM or DDM. In the light of the search for solvents meeting the requirements of green chemistry, it is necessary to extend and deepen experiments using water, which, as described above, can effectively contribute to the fungicidal effects of a mixture of lichen substances, although rather at relatively high concentrations in the extract.

In the context of the use of lichen extracts, this meta-analysis has shown the following.

1. The greatest anti-fusarial potential is demonstrated by extracts from the thallus of relatively easily available epiphytic macrolichen species (*Alectoria sarmentosa*, *Evernia divaricata*, *E. prunastri*, *Flavoparmelia caperata*, *Hypotrachyna cirrhata*, *Leucodermia leucomelos*, *Parmotrema austrosinense*, *P. reticulatum*, *Physcia aipolia*, *Pseudevernia furfuracea* and *Roccella montagnei*), two epigeic (*Cladonia mitis* and *C. rangiferina*), including a mixture of both species, and one epilithic species (*Umbilicaria nylanderiana*).
2. An extract from *Evernia divaricata* can compete with the potential of amphotericin B against *F. acuminatum*; an extract from *Flavoparmelia caperata* can replace flucytosine and fluconazole, extracts from *Parmotrema austrosinense*, *Physcia aipolia* and *Roccella montagnei* may replace bavistin, flucytosine and ketoconazole; the effectiveness of an extract from *Umbilicaria nylanderiana* is comparable to flucytosine, miconazole, amphotericin B and ketoconazole against *F. oxysporum*; an *Alectoria sarmentosa* extract may prove to be a potential replacement for the systemic fungicide bavistin against *F. oxysporum*.
3. It is advisable to use a combination of extracts based on thalli from more than one species.
4. It is advisable to undertake more extensive experiments using poorly studied species of fungi of the genus *Fusarium* and to focus more attention on the potential, at least, of epigeic lichens.
5. It is necessary to pay more attention to lichen species containing atranorin, usnic and fumarprotocetric acids in higher concentrations (see also point 3 above) and as well as the lichens with secondary compounds listed below in the point 1 regarding individual secondary metabolites.
6. Field experiments using lichen extracts with the highest fungistatic potential as plant protection agents must be carried out.

In the context of the use of individual secondary metabolites of lichens, the meta-analysis conducted showed that:

1. the compounds with the highest fungistatic potential include 2-hydroxy-4-methoxy-3,6-dimethylbenzoic acid, atranorin, barbatolic acid, diffractaic acid, evernyl, lecanoric acid, methyl orsellinate and norstictic, orsellinic, protocetraric, protolichesterinic, sekikaic and (iso)usnic acids,
2. their potential does not appear to be related to membership of a particular biochemical group,
3. no compounds with strong fungistatic potential against *F. culmorum* have been found so far; high inhibitory effect against *F. fujikuroi* was shown by barbatolic, diffractaic, lecanoric, norstictic, orsellinic, protocetraric and usnic acids; reduction in mycelium growth of *F. oxysporum* can be obtained by the use of 2-hydroxy-4-methoxy-3,6-dimethylbenzoic acid, 2'-O-methylanziaic acid, atranorin, fumarprotocetraric acid, gyrophoric acid, lecanoric acid, methyl evernate, physodalic acid, protocetraric acid and usnic acid; barbatolic acid can be used against *F. solani*; all these metabolites can potentially replace at least flucytosine and fluconazole, to a lesser extent itraconazole, ketoconazole, clotrimazole and miconazole; in the case of *F. udum*, further experiments on MIC and IZ potential should be performed for comparison with reference substances,
4. a research methodology using mixed secondary metabolites is recommended, for example those that can potentially exert a strong effect on lichen extracts from a given lichen species,
5. it is recommended to extend experiments on solubility of metabolites in water using different factors (e.g., temperature, pH),
6. it is recommended that experiments be carried out on secondary metabolites that are difficult to dissolve in water, such as lichesterinic, protolichesterinic or rangiformic acids (higher aliphatic fatty acids) and that studies be carried out on the preservation potential of foods.

## Supplementary Information

Below is the link to the electronic supplementary material.

Supplementary file1 (DOCX 301 KB)

## Data Availability

Not applicable.

## References

[CR1] Agrawal S, Deshmukh SK, Reddy MS, Prasad R, Goel M (2020). Endolichenic fungi: a hidden source of bioactive metabolites. S Afr J Bot.

[CR2] Akbulut G, Yildiz A (2010). An overview to lichens: the nutrient composition of some species. Kafkas Üniversitesi Fen Bilimleri Enstitüsü Dergisi.

[CR3] Alastruey-Izquierdo A, Cuenca-Estrella M, Monzón A, Mellado E, Rodríguez-Tudela JL (2008). Antifungal susceptibility profile of clinical *Fusarium* spp. isolates identified by molecular methods. J Antimicrob Chemother.

[CR4] Al-Hatmi AMS, van Diepeningen AD, Curfs-Breuker I, de Hoog GS, Meis JF (2015). Specific antifungal susceptibility profiles of opportunists in the *Fusarium fujikuroi* complex. J Antimicrob Chemother.

[CR5] Al-Hatmi AMS, Bonifaz A, Ranque S, de Hoog GS, Verweij PE, Meis JF (2018). Current antifungal treatment of fusariosis. Int J Antimicrob Agents.

[CR6] Anjali DB, Mohabe S, Reddy AM, Nayaka S (2015). Efficacy of a potential lichen *Parmotrema andinum* (Müll. Arg.) Hale against pathogenic microorganisms. J New Biol Rep.

[CR7] Anjali DB, Mohabe S, Reddy AM, Nayaka S (2015). Antimicrobial activities of 2-propanol crude extract of lichen *Parmotrema tinctorum* (Despr. ex. Nyl.) Hale, collected from Eastern Ghats, India. Curr Res Environ Appl Mycol.

[CR8] Aravind SR, Sreelekha TT, Dileep Kumar BS, Nishanth KS, Mohandas C (2014). Characterization of three depside compounds from a Western Ghat lichen *Parmelia erumpens* Kurok with special reference to antimicrobial and anticancer activity. RSC Adv.

[CR9] Arie T (2019). *Fusarium* diseases of cultivated plants, control, diagnosis, and molecular and genetic studies. J Pestic Sci.

[CR10] Armaleo D, Zhang Y, Cheung S (2008). Light might regulate divergently depside and depsidone accumulation in the lichen *Parmotrema hypotropum* by affecting thallus temperature and water potential. Mycologia.

[CR11] Aslan A, Güllüce M, Sökmen M, Adɩgüzel A, Sahin F (2006). Antioxidant and antimicrobial properties of the lichens *Cladonia foliacea*, *Dermatocarpon miniatum*, *Evernia divaricata*, *Evernia prunastri*, and *Neofuscella pulla*. Pharm Biol.

[CR12] Avis TJ, Bélanger RR (2001). Specificity and mode of action of the antifungal fatty acid cis-9-heptadecenoic acid produced by *Pseudozyma flocculosa*. Appl Environ Microbiol.

[CR13] Babiah PS, Upreti DK, John SA (2014). Fungicidal efficacy of a foliose lichen *Flavoparmelia caperata* (L.) Hale against phytopathogenic fungi. Int J Curr Res Biosci Plant Biol.

[CR14] Babiah PS, Upreti DK, John SA (2014). An *in vitro* analysis of antifungal potential of lichen species *Parmotrema reticulatum* against phytopathogenic fungi. Int J Curr Microbiol App Sci.

[CR15] Badridze G, Chkhubianishvili E, Rapava L, Kikvidze M, Chigladze L, Tsiklauri N, Tsilosani K, Kupradze I, Chanishvili S (2019). Active metabolites of some lichens growing in Georgia. J Biodivers Environ Sci.

[CR16] Balasubramanian M, Nirmala P (2014). Antimycobacterial activity of foliose lichens on plant and animal pathogens. Int J Pharm Sci Res.

[CR17] Bandoni RJ, Towers GHN (1967). Degradation of usnic acid by microorganisms. Can J Biochem.

[CR18] Bates ST, Cropsey GWG, Caporaso JG, Knight R, Fierer N (2011). Bacterial community associated with the lichen symbiosis. Appl Environ Microbiol.

[CR19] Batista BG, de Chaves MA, Reginatto P, Saraiva OJ, Fuentefria AM (2020). Human fusariosis: an emerging infection that is difficult to treat. J Braz Soc Trop Med.

[CR20] Begora M, Fahselt D (2001). Photolability of secondary compounds in some lichen species. Symbiosis.

[CR21] Bennett JW, Klich M (2003). Mycotoxins. Clin Microbiol Rev.

[CR22] Bhattacharyya A, Sinha M, Singh H, Patel RS, Ghosh S, Sardana K, Ghosh S, Sengupta S (2020). Mechanistic insight into the antifungal fffects of a fatty acid derivative against drug-resistant fungal infections. Front Microbiol.

[CR23] Borman AM, Fraser M, Palmer MD, Szekely A, Houldsworth M, Patterson Z, Johnson EM (2017). MIC distributions and evaluation of fungicidal activity for amphotericin B, itraconazole, voriconazole, posaconazole and caspofungin and 20 species of pathogenic filamentous fungi determined using the CLSI broth microdilution method. J Fungi.

[CR24] Boustie J, Tomasi S, Grube M (2011). Bioactive lichens metabolites: alpine habitats as an untapped source. Phytochem Rev.

[CR25] Burkin AA, Kononenko GP (2014). Secondary fungal metabolites (mycotoxins) in lichens of different taxonomic groups. Biol Bull.

[CR26] Burkin AA, Kononenko GP (2015). Metabolites of toxigenic fungi in lichens of genera *Alectoria*, *Bryoria*, *Evernia*, *Pseudevernia*, and *Usnea*. Biol Bull.

[CR27] Burkin AA, Kononenko GP (2015). Metabolites of toxigenic fungi in lichens of genera *Nephroma*, *Peltigera*, *Umbilicaria*, and *Xanthoria*. Biol Bull.

[CR28] Burkin AA, Kononenko GP, Tolpysheva TYu (2013). Enzyme immunoassay of usnic acid in lichens. Appl Biochem Microbiol.

[CR29] Cankılıç MY, Sarıözlü NY, Candan M, Tay F (2017). Screening of antibacterial, antituberculosis and antifungal effects of lichen *Usnea florida* and its thamnolic acid constituent. Biomed Res.

[CR30] Cansaran Duman D, Aras S, Atakol O (2008). Determination of usnic acid content in some lichen species found in Anatolia. J Appl Biol Sci.

[CR31] Carbonero ER, Sassaki GL, Stuelp PM, Gorin PAJ, Woranovicz-Barreira SM, Iacomini M (2001). Comparative studies of the polysaccharides isolated from lichenized fungi of the genus *Cladonia*: significance as chemotypes. FEMS Microbiol Lett.

[CR32] Cardile V, Graziano ACE, Avola R, Piovano M, Russo A (2017). Potential anticancer activity of lichen secondary metabolite physodic acid. Chem Biol Interact.

[CR33] Chróst A (2016). Grzyby pleśniowe w środowisku człowieka – zagrożenie i skutki zdrowotne [Health risks associated with the environmental presence of toxigenic moulds]. Medycyna Doświadczalna i Mikrobiologia.

[CR34] CLSI (2015a) Methods for dilution antimicrobial susceptibility tests for bacteria that grow aerobically. Approved standard—10th edition M07-A10, 35(2), Wayne

[CR35] CLSI (2015b) Performance standards for antimicrobial disk susceptibility tests. Approved standard—12th edition M02-A12, 35(1), Wayne

[CR36] CLSI (2017) Reference method for broth dilution antifungal susceptibility testing of yeasts. 4th edition M27, Wayne

[CR37] Çobanoğlu G, Sesal C, Gökmen B, Çakar S (2010). Evaluation of the antimicrobial properties of some lichens. South West J Hortic Biol Environ.

[CR38] Cordeiro LMC, Stocker-Wörgötter E, Gorin PAJ, Iacomini M (2003). Comparative studies of the polysaccharides from species of the genus *Ramalina*—lichenized fungi—of three distinct habitats. Phytochemistry.

[CR39] Cordeiro LMC, Stocker-Wörgötter E, Gorin PAJ, Iacomini M (2004). Elucidation of polysaccharide origin in *Ramalina peruviana* symbiosis. FEMS Microbiol Lett.

[CR40] Cordeiro LMC, Carbonero ER, Sassaki GL, Reis RA, Stocker-Wörgötter E, Gorin PAJ, Iacomini M (2005). A fungus-type β-galactofuranan in the cultivated *Trebouxia* photobiont of the lichen *Ramalina gracilis*. FEMS Microbiol Lett.

[CR41] Cordeiro LMC, Sassaki GL, Iacomini M (2007). First report on polysaccharides of *Asterochloris* and their potential role in the lichen symbiosis. Int J Biol Macromol.

[CR42] Cordeiro LMC, Messias D, Sassaki GL, Gorin PAJ, Iacomini M (2011). Does aposymbiotically cultivated fungus *Ramalina* produce isolichenan?. FEMS Microbiol Lett.

[CR43] Czeczuga B, Christensen StN (1994). The carotenoids of *Pseudevernia furfuracea* along a North-South gradient in Europe. Feddes Repert.

[CR44] Czeczuga B, Lallemant R (1993). Investigations on carotenoids in lichens, 44: carotenoids of some lichens from France. Acta Botan Gallica.

[CR45] Devashree PA, Dikshit A, Nayaka S (2019). Antimicrobial activity of *Roccella montagnei* against pathogenic microorganisms. Int J Adv Res Ideas Innov Technol.

[CR46] Dignani MC, Anaissie E (2004). Human fusariosis. Clin Microbiol Infect.

[CR47] Dongzhen F, Xilin L, Xiaorong C, Wenwu Y, Yunlu H, Yi C, Jia C, Zhimin L, Litao G, Tuhong W, Xu J, Chunsheng G (2020). *Fusarium* species and *Fusarium oxysporum* species complex genotypes associated with yam wilt in South-Central China. Front Microbiol.

[CR48] Elix JA (2014). A catalogue of standardized chromatographic data and biosynthetic relationships for lichen substances.

[CR49] Elix JA, Stocker-Wörgötter E, Nash TH (2008). Biochemistry and secondary metabolites. Lichen biology.

[CR50] Elkhateeb WA, Daba GM, Sheir D, Nguyen T-D, Hapuarachchi KK, Thomas PW (2020). Mysterious world of lichens: highlights on their history, applications, and pharmaceutical potentials. Nat Prod J.

[CR51] Elshobary ME, Osman ME, Abo-Shady AM, Komatsu E, Perreault H, Sorensen J, Piercey-Normore MD (2016). Algal carbohydrates affect polyketide synthesis of the lichen-forming fungus *Cladonia rangiferina*. Mycologia.

[CR52] Esnakula AK, Summers I, Naab TJ (2013). Fatal disseminated *Fusarium* infection in a human immunodeficiency virus positive patient. Case Rep Infect Dis.

[CR53] Farkas E, Biró B, Szabó K, Veres K, Zs C, Engel R (2020). The amount of lichen secondary metabolites in *Cladonia foliacea* (Cladoniaceae, lichenised Ascomycota). Acta Bot Hungar.

[CR54] Ferron S, Berry O, Olivier-Jimenez D, Rouaud I, Boustie J, Lohézic-Le Dévéhat F, Poncet R (2020). Chemical diversity of five coastal *Roccella* species from mainland France, the Scattered Islands, and São Tomé and Príncipe. Plant Fungal Syst.

[CR55] Freysdottir J, Omarsdottir S, Ingolfsdottir K, Vikingsson A, Olafsdottir ES (2008). *In vitro* and *in vivo* immunomodulating effects of traditionally-prepared extract and purified compounds from *Cetraria islandica*. Proc Nutr Soc.

[CR56] Furmanek Ł (2019). Metabolizm wtórny porostów jako źródło potencjalnych związków przeciwgrzybicznych [Secondary metabolism of lichens as a source of potential antifungal compounds]. AURA.

[CR57] Furmanek Ł, Czarnota P, Seaward MRD (2019). Antifungal activity of lichen compounds against dermatophytes: a review. J Appl Microbiol.

[CR58] Furmanek Ł, Czarnota P, Seaward MRD (2022). The effect of lichen secondary metabolites on *Aspergillus* fungi. Arch Microbiol.

[CR59] Gazo SMT, Santiago KAA, Tjitrosoedirjo SS, Dela Cruz TEE (2019). Antimicrobial and herbicidal activities of the fruticose lichen *Ramalina* from Guimaras Island. Philipp Biotropia.

[CR60] Goel M, Singh B (2015). Efficacy of major chemical constituents isolated from Himalayan lichen *Ramalina roesleri* Nyl. against soil borne plant pathogenic fungi. Int J Appl Eng Res.

[CR61] Goel M, Dureja P, Rani A, Uniyal PL, Laatsch H (2011). Isolation, characterization and antifungal activity of major constituents of the Himalayan lichen *Parmelia reticulata* Tayl. J Agric Food Chem.

[CR62] Goel M, Sharma PK, Dureja P, Rani A, Uniyal PL (2011). Antifungal activity of extracts of the lichens *Parmelia reticulata*, *Ramalina roesleri*, *Usnea longissima* and *Stereocaulon himalayense*. Arch Phytopathol Plant Protect.

[CR63] Goga M, Elečko J, Marcinčinová M, Ručová D, Bačkorová M, Bačkor M, Merillon JM, Ramawat K (2018). Lichen metabolites: an overview of some secondary metabolites and their biological potential. Co-evolution of secondary metabolites. Reference series in phytochemistry.

[CR64] Grimm M, Grube M, Schiefelbein U, Zühlke D, Bernhardt J, Riedel K (2021). The lichens’ microbiota, still a mystery?. Front Microbiol.

[CR65] Grube M, Cardinale M, de Castro Jr JV, Mūller H, Berg G (2009). Species-specific structural and functional diversity of bacterial communities in lichen symbioses. ISME J.

[CR66] Grube M, Cernava T, Soh J, Fuchs S, Aschenbrenner I, Lassek C, Wegner U, Becher D, Riedel K, Sensen CW, Berg G (2015). Exploring functional contexts of symbiotic sustain within lichen-associated bacteria by comparative omics. ISME J.

[CR67] Grujičić D, Stošić I, Kosanić M, Stanojković T, Raković B, Milošević-Djordjević O (2014). Evaluation of *in vitro* antioxidant, antimicrobial, genotoxic and anticancer activities of lichen *Cetraria islandica*. Cytotechnology.

[CR68] Guarro J, Pujol I, Mayayo E (1999). *In vitro* and *in vivo* experimental activities of antifungal agents against *Fusarium solani*. Antimicrob Agents Chemother.

[CR69] Gulluce M, Aslan A, Sokmen M, Sahin F, Adiguzel A, Agar G, Sokmen A (2006). Screening the antioxidant and antimicrobial properties of the lichens *Parmelia saxatilis*, *Platismatia glauca*, *Ramalina pollinaria*, *Ramalina polymorpha* and *Umbilicaria nylanderiana*. Phytomedicine.

[CR70] Halama P, van Haluwin C (2004). Antifungal activity of lichen extracts and lichenic acids. Biocontrol.

[CR71] Hanuš LO, Temina M, Dembitsky VM (2007). Antibacterial and antifungal activities of some phenolic metabolites isolated from lichenized ascomycete *Ramalina lacera*. Nat Prod Commun.

[CR72] Hawksworth D, Grube M (2020). Lichens redefined as complex ecosystems. New Phytol.

[CR73] Hawrył A, Hawrył M, Hajnos-Stolarz A, Abramek J, Bogucka-Kocka A, Komsta Ł (2020). HPLC fingerprint analysis with the antioxidant and cytotoxic activities of selected lichens combined with the chemometric calculations. Molecules.

[CR74] Herkert PF, Al-Hatmi AMS, de Oliveira Salvador GL, Muro MD, Pinheiro RL, Nucci M, Queiroz-Telles F, de Hoog GS, Meis JF (2019). Molecular characterization and antifungal susceptibility of clinical *Fusarium* species from Brazil. Front Microbiol.

[CR75] Hickey BJ, Lumsden AJ, Cole AIJ, Walker JRL (1990). Antibiotic compounds from New Zealand plants: methyl haematommate, an anti-fungal agent from *Stereocaulon ramulosum*. N Z Nat Sci.

[CR76] Honegger R, Esser K, Deising HB (2009). Lichen-forming fungi and their photobionts. The Mycota. A comprehensive treatise on fungi as experimental systems for basic and applied research.

[CR77] Isaac MR, Leyva-Mir SG, Sahagún-Castellanos J, Câmara-Correia K, Tovar-Pedraza JM, Rodríguez-Pérez JE (2018). Occurrence, identification, and pathogenicity of *Fusarium* spp. associated with tomato wilt in Mexico. Notulae Botanicae Horti Agrobotanici Cluj-Napoca.

[CR78] Ivanovic J, Meyer F, Misic D, Asanin J, Jaeger P, Zizovic I, Eggers R (2013). Influence of different pre-treatment methods on isolation of extracts with strong antibacterial activity from lichen *Usnea barbata* using carbon dioxide as a solvent. J Supercrit Fluids.

[CR79] Ji F, He D, Olaniran AO, Mokoena MP, Xu J, Shi J (2019). Occurrence, toxicity, production and detection of *Fusarium* mycotoxin: a review. Food Prod Process Nutr.

[CR80] Jin J-Q, Rao Y, Bian X-L, Zeng A, Yang G-D (2013). Solubility of (+)-usnic acid in water, ethanol, acetone, ethyl acetate and *n*-hexane. J Solut Chem.

[CR81] Jüriado I, Kaasalainen U, Jylhä M, Rikkinen J (2019). Relationships between mycobiont identity, photobiont specificity and ecological preferences in the lichen genus *Peltigera* (Ascomycota) in Estonia (northeastern Europe). Fungal Ecol.

[CR82] Kahriman N, Yazici K, Arslan T, Aslan A, Karaoglu SA, Yayli N (2011). Chemical composition and antimicrobial activity of the essential oils from *Evernia prunastri* (L.) Ach and *Evernia divaricata* (L) Ach. Asian J Chem.

[CR83] Karabulut G, Ozturk S (2015). Antifungal activity of *Evernia prunastri*, *Parmelia sulcata*, *Pseudevernia furfuracea* var. *furfuracea*. Pak J Bot.

[CR84] Karim NFA, Mohd M, Nor NMIM, Zakaria L (2016). Saprophytic and potentially pathogenic *Fusarium* species from peat soil in Perak and Pahang. Trop Life Sci Res.

[CR85] Kellogg JJ, Raja HA (2017). Endolichenic fungi: a new source of rich bioactive secondary metabolites on the horizon. Phytochem Rev.

[CR86] Khadhri A, Mendili M, Araújo MEM, Seaward MRD (2019). Comparative study of secondary metabolites and bioactive properties of the lichen *Cladonia foliacea* with and without the lichenicolous fungus *Heterocephalacria bachmannii*. Symbiosis.

[CR87] König GM, Wright AD (1999). ^1^H and ^13^C-NMR and biological activity investigations of four lichen-derived compounds. Phytochem Anal.

[CR88] Kosanić M, Ranković B (2011). Antioxidant and antimicrobial properties of some lichens and their constituents. J Med Food.

[CR89] Kosanić M, Ranković B (2011). Antibacterial and antifungal activity of different lichens extracts and lichen acid. Res J Biotechnol.

[CR90] Kosanić M, Ranković B, Sukdolak S (2010). Antimicrobial activity of the lichen *Lecanora frustulosa* and *Parmeliopsis hyperopta* and their divaricatic acid and zeorin constituents. Afr J Microbiol Res.

[CR91] Kosanić M, Ranković B, Stanojković T (2012). Antioxidant, antimicrobial and anticancer activity of 3 *Umbilicaria* species. J Food Sci.

[CR92] Kosanić MM, Ranković BR, Stanojković TP (2012). Antioxidant, antimicrobial and anticancer activities of three *Parmelia* species. J Sci Food Agric.

[CR93] Kosanić M, Ranković B, Stanojković T (2013). Investigation of selected Serbian lichens for antioxidant, antimicrobial and anticancer properties. J Anim Plant Sci.

[CR94] Kosanić M, Ranković B, Stanojković T, Stošić I, Grujičić D, Milošević-Djordjević O (2016). *Lasallia pustulata* lichen as a possible natural antigenotoxic, antioxidant, antimicrobial and anticancer agent. Cytotechnology.

[CR95] Kosanić M, Ristić S, Stanojković T, Manojlović N, Ranković B (2018). Extracts of five *Cladonia* lichens as sources of biologically active compounds. Farmacia.

[CR96] Köycü ND (2018) Effect on *Fusarium culmorum* of fungicides used in wheat seed. In: International congress on engineering and life science, p 593–601

[CR97] Kumar K, Siva B, Sarma VUM, Mohabe S, Reddy AM, Boustie J, Tiwari AK, Rao NR, Babu KS (2018). UPLCMS/MS quantitative analysis and structural fragmentation study of five *Parmotrema* lichens from the Eastern Ghats. J Pharm Biomed Anal.

[CR98] Latkowska E, Bialczyk J, Węgrzyn M, Erychleb U (2019). Host species affects the phenolic compounds content in *Hypogymnia physodes* (L.) Nyl. Thalli. Allelopath J.

[CR99] Legouin B, Lohézic-Le DF, Ferron S, Rouaud I, Le Pogam P, Cornevin L, Bertrand M, Boustie J (2017). Specialized metabolites of the lichen *Vulpicida pinastri* act as photoprotective agents. Molecules.

[CR100] Li D-W, Yang CS (2004). Fungal contamination as a major contributor to Sick Building Syndrome. Adv Appl Microbiol.

[CR101] Lücking R, Hodkinson BP, Leavitt SD (2017). The 2016 classification of lichenized fungi in the Ascomycota and Basidiomycota—approaching one thousand genera. Bryologist.

[CR102] Mansoory D, Roozbahany NA, Mazinany H, Samimagan A (2003). Chronic *Fusarium* infection in an adult patient with undiagnosed chronic granulomatous disease. Clin Infect Dis.

[CR103] Maulidiyah M, Darmawan A, Usman U, Musdalifah A, Salim LOA, Nurdin M (2021). Antioxidant activity of secondary metabolite compounds from lichen *Teloschistes flavicans*. Biointerface Res Appl Chem.

[CR104] Millot M, Girardot M, Dutreix L, Mambu L, Imbert C (2017). Antifungal and anti-biofilm activities of acetone lichen extracts against *Candida albicans*. Molecules.

[CR105] Mishra T, Shukla S, Meena S, Singh R, Pal M, Upreti DK, Datta D (2017). Isolation and identification of cytotoxic compounds from a fruticose lichen *Roccella montagnei*, and it’s *in silico* docking study against CDK-10. Braz J Pharmacogn.

[CR106] Muggia L, Grube M (2018). Fungal diversity in lichens: from extremotolerance to interactions with algae. Life (basel).

[CR107] Nag PK (2019). Sick building syndrome and other building-related illnesses. Office buildings.

[CR108] Nash TH, Ryan BD, Diederich P, Gries C, Bungartz F (2002). Lichen flora of the greater sonoran desert region.

[CR109] Nelson PE, Dignani MC, Anaissie EJ (1994). Taxonomy, biology, and clinical aspects of *Fusarium* species. Clin Microbiol Rev.

[CR110] Norouzi H, Azizi A, Gholami M, Sohrabi M, Boustie J (2020). Chemotype variations among lichen ecotypes of *Umbilicaria aprina* as revealed by LC-ESI-MS/MS: a survey of antioxidant phenolics. Environ Sci Pollut Res.

[CR111] Nybakken L, Julkunen-Tiitto R (2006). UV-B induces usnic acid in reindeer lichens. Lichenologist.

[CR112] Olafsdottir ES, Ingólfsdottir K (2001). Polysaccharides from lichens: structural characteristics and biological activity. Planta Med.

[CR113] Oldenburg E, Höppner F, Ellner F, Weinert J (2017). *Fusarium* diseases of maize associated with mycotoxin contamination of agricultural products intended to be used for food and feed. Mycotoxin Res.

[CR114] Parrot D, Peresse T, Hitti E, Carrie D, Grube M, Tomasi S (2015). Qualitative and spatial metabolite profiling of lichens by a LC-MS approaches combined with optimized extraction. Phytochem Anal.

[CR115] Pitt JI, Hocking AD (2009). Fungi and food spoilage.

[CR116] Prashith Kekuda TR, Ranjitha MC, Ghazala F, Vidya P, Vinayaka KS (2015). Antimicrobial activity of selected corticolous macrolichens. Sci Technol Arts Res J.

[CR117] Prokop’ev IA, Yatsyna AP, Poryadina LN, Filippova GV, Shavarda AL (2018). Phenolic metabolites of lichens in the genus *Cladonia* growing in Belarus and Yakutia. Chem Nat Compd.

[CR118] Prokopiev IA, Shavarda AL, Filippova GV, Shein AA (2017). Application of high-performance liquid chromatography to the determination of the concentration of lichen secondary metabolites. J Anal Chem.

[CR119] Pujol I, Guarro J, Gené J, Sala J (1997). *In-vitro* antifungal susceptibility of clinical and environmental *Fusarium* spp. strains. J Antimicrob Chemother.

[CR120] Ráduly Z, Szabó L, Madar A, Pócsi I, Csermoch L (2020). Toxicological and medical aspects of *Aspergillus*-derived mycotoxins entering the feed and food chain. Front Microbiol.

[CR121] Ranković B, Kosanić M (2012). Antimicrobial activities of different extracts of *Lecanora atra*, *Lecanora muralis*, *Parmelia saxatilis*, *Parmelia sulcata* and *Parmeliopsis ambigua*. Pak J Bot.

[CR122] Ranković B, Kosanić M, Ranković B (2015). Lichens as a potential source of bioactive secondary metabolites. Lichen secondary metabolites. Bioactive properties and pharmaceutical potential.

[CR123] Ranković B, Mišić M (2007). Antifungal activity of extracts of the lichens *Alectoria sarmentosa* and *Cladonia rangiferina*. Mikologiya i Fitopalogiya.

[CR124] Ranković B, Mišić M (2008). The antimicrobial activity of the lichen substances of the lichens *Cladonia furcata*, *Ochrolechia androgyna*, *Parmelia caperata* and *Parmelia conspersa*. Pharm Biotechnol.

[CR125] Ranković B, Mišić M, Sukdolak S (2007). Evaluation of antimicrobial activity of the lichens *Lasallia pustulata*, *Parmelia sulcata*
*Umbilicaria crustulosa* and *Umbilicaria cylindrica*. Microbiology.

[CR126] Ranković B, Mišić M, Sukdolak S, Milosavljević D (2007). Antimicrobial activity of the lichens *Aspicilia cinerea*, *Collema cristatum*, *Ochrolechia adrogyna*, *Physcia aipolia* and *Physcia caesia*. Ital J Food Sci.

[CR127] Ranković B, Mišić M, Sukdolak S (2008). The antimicrobial activity of substances derived from the lichens *Physcia aipolia*, *Umbilicaria polyphylla*, *Parmelia caperata* and *Hypogymnia physodes*. World J Microbiol Biotechnol.

[CR128] Ranković B, Mišić M, Sukdolak S (2009). Antimicrobial activity of extracts of the lichens *Cladonia furcata*, *Parmelia caperata*, *Parmelia pertus*a, *Hypogymnia physodes* and *Umbilicaria polyphylla*. Biologia.

[CR129] Ranković B, Ranković D, Kosanić M, Marić D (2010). Antioxidant and antimicrobial properties of the lichens *Anaptychia ciliaris*, *Nephroma parile*, *Ochrolechia tartarea* and *Parmelia centrifuga*. Central Eur J Bot.

[CR130] Ranković B, Ranković D, Marić D (2010). Antioxidant and antimicrobial activity of some lichen species. Microbiology.

[CR131] Ranković B, Kosanić M, Sukdolak S (2010c) Antimicrobial activity of some lichens and their components. Recent Adv Clin Med 279–286

[CR132] Ranković BR, Kosanić MM, Stanojković TP (2011). Antioxidant, antimicrobial and anticancer activity of the lichens *Cladonia furcata*, *Lecanora atra* and *Lecanora muralis*. BMC Complement Altern Med.

[CR133] Ranković B, Kosanić M, Stanojković T (2014). *Stereocaulon paschale* lichen as antioxidant, antimicrobial and anticancer agent. Farmacia.

[CR134] Ristić S, Ranković B, Kosanić M, Stamenković S, Stanojković T, Sovrlić M, Manojlović N (2016). Biopharmaceutical potential of two *Ramalina* lichens and their metabolites. Curr Pharm Biotechnol.

[CR135] Ristić S, Ranković B, Kosanić M, Stanojković T, Stamenković S, Vasiljević P, Manojlović I, Manojlović N (2016). Phytochemical study and antioxidant, antimicrobial and anticancer activities of *Melanelia subaurifera* and *Melanelia fuliginosa* lichens. J Food Sci Technol.

[CR136] Rundel P (1978). The ecological role of secondary lichen substances. Biochem Syst Ecol.

[CR137] Ruotsalainen AL, Koopmann R, Hyvärinen M (2009). Lichen substances and the growth of root associated fungi *Phialocephala fortinii* and *Rhizoscyphus ericae* (Ascomycetes) in pure culture. Sydowia.

[CR138] Ruthes AC, Komura DL, Carbonero ER, Cordeiro LMC, Reis RA, Sassaki GL, Gorin PAJ, Iacomini M (2008). Polyssaccharides present in cultivated *Teloschistes flavicans* symbiosis: comparison with those of the thallus. Plant Physiol Biochem.

[CR139] Sargsyan R, Gasparyan A, Tadevosyan G, Panosyan H (2021). Antimicrobial and antioxidant potentials of non-cytotoxic extracts of corticolous lichens sampled in Armenia. AMB Express.

[CR140] Sarıözlü NY, Cankılıç MY, Candan M, Tay T (2016). Antimicrobial activity of lichen *Bryoria capillaris* and its compound barbatolic acid. Biomed Res.

[CR141] Segatore B, Bellio P, Setaci D, Brisdelli F, Piovano M, Garbarino JA, Nicoletti M, Amicosante G, Perilli M, Celenza G (2012). *In vitro* interaction of usnic acid in combination with antimicrobial agents against methicillin-resistant *Staphylococcus aureus* clinical isolates determined by FICI and ΔE model methods. Phytomedicine.

[CR142] Shahi SK, Shukla AC, Dikshit A, Uperti DK (2001). Broad spectrum antifungal properties of the lichen *Heterodermia leucomela*. Lichenologist.

[CR143] Shahi SK, Patra M, Dikshit A, Upreti DK (2003). *Parmelia cirrhatum*: a potential source of [a] broad spectrum [of] natural antifungal [properties]. Phytother Res.

[CR144] Shiromi PSAI, Hewawasam RP, Jayalal RGU, Rathnayake H, Wijayaratne WMDGB, Wanniarachchi D (2021). Chemical composition and antimicrobial activity of two Sri Lankan lichens, *Parmotrema rampoddense*, and *Parmotrema tinctorum* against methicillin-sensitive and methicillin-resistant *Staphylococcus aureus*. Evid Based Complement Altern Med.

[CR145] Shivanna R, Garampalli RH (2014). Efficacy of lichen extracts as biocontrol agents against *Fusarium oxysporum* f. sp. *capsici*. Adv Appl Sci Res.

[CR146] Shivanna R, Garampalli RH (2015). Evaluation of fungistatic potential of lichen extracts against *Fusarium solani* (Mart.) Sacc. causing rhizome rot disease in ginger. J Appl Pharm Sci.

[CR147] Shukla I, Azmi L, Gautam A, Shukla SK, Rao ChV (2018). Lichens are the next promising candidates for medicinally active compounds. Int J Phytopharm.

[CR148] Smeds AI, Kytöviita M-M (2010). Determination of usnic and perlatolic acids and identification of olivetoric acids in Northern reindeer lichen (*Cladonia stellaris*) extracts. Lichenologist.

[CR149] Smith CW, Aptroot A, Coppins BJ, Fletcher A, Gilbert OL, James PW, Wolseley PA (2009). The lichens of Great Britain and Ireland.

[CR150] Song Y, Liu X, Yang Z, Meng X, Xue R, Yu J, Al-Hatmi AMS, de Hoog GS (2021). Molecular and MALDI-ToF MS differentiation and antifungal susceptibility of prevalent clinical *Fusarium* species in China. Mycoses.

[CR151] Spribille T, Tuovinen V, Resl P, Vanderpool D, Wolinski H, Aime C, Schneider K, Stabentheiner E, Toome-Heller M, Thor G, Mayrhofer H, Johannesson H, McCutcheon JP (2016). Basidiomycete yeasts in the cortex of ascomycete macrolichens. Science.

[CR152] Spribille T, Tagirdzhanova G, Goyette S, Tuovinen V, Case R, Zandberg WF (2020). 3D biofilms: in search of the polysaccharides holding together lichen symbioses. FEMS Microbiol Lett.

[CR153] Staśkiewicz K, Szakiel A, Danielewska A, Maciąg M (2020). Pionierskie grzyby zlichenizowane, czyli porosty jako samowystarczalne źródło unikalnych metabolitów wtórnych. Osiągnięcia, wyzwania oraz problemy nauk przyrodniczych i medycznych.

[CR154] Surayot U, Yelithao K, Tabarsa M, Lee D-H, Palanisamy S, Prabhu NM, Lee JH, You SG (2019). Structural characterization of a polysaccharide from *Certaria islandica* and assessment of immunostimulatory activity. Process Biochem.

[CR155] Tatipamula VB, Vedula GS, Sastry AVS (2019). Chemical and pharmacological evaluation of manglicolous lichen *Roccella montagnei* Bel em. D. D. Awasthi. Future J Pharm Sci.

[CR156] Tekiela A, Furmanek Ł, Andrusiewicz M, Bara G, Seaward MRD, Kapusta I, Czarnota P (2021). Can lichen secondary compounds impact upon the pathogenic soil fungi *Fusarium oxysporum* and *F. avenaceum*?. Folia Cryptogam Estonica.

[CR157] Thadhani VM, Choudhary MI, Khan S, Karunaratne V (2012). Antimicrobial and toxicological activities of some depsides and depsidones. J Natl Sci Found.

[CR158] Thippeswamy B, Sushma NR, Naveenkumar KJ (2013). Evaluation of antimicrobial property of lichen *Parmelia perlata*. Afr J Pharm Pharmacol.

[CR159] Tiwari P, Rai H, Upreti DK, Trivedi S, Shukla P (2011). Assessment of antifungal activity of some Himalayan foliose lichens against plant pathogenic fungi. Am J Plant Sci.

[CR160] Tiwari P, Rai H, Upreti DK, Trivedi S, Shukla P (2011). Antifungal activity of a common Himalayan foliose lichen *Parmotrema tinctorum* (Despr. ex. Nyl.) Hale. Nat Sci.

[CR161] Tripathi AH, Negi N, Gahtori R, Kumari A, Joshi P, Tewari LM, Joshi Y, Bajpai R, Upreti DK, Upadhyay SK (2022). A review of anti-cancer and related properties of lichen-extracts and metabolites. Anticancer Agents Med Chem.

[CR162] Tuovinen V, Ekman S, Thor G, Vanderpool D, Spribille T, Johannesson H (2019). Two Basidiomycete fungi in the cortex of wolf lichens. Curr Biol.

[CR163] Türk AÖ, Yılmaz M, Kıvanç M, Türk H (2003). The antimicrobial activity of extracts of the lichen *Cetraria aculeata* and its protolichesterinic acid constituent. Zeitschrift Für Naturforschung C.

[CR164] Türk H, Yılmaz M, Tay T, Türk AÖ, Kıvanç M (2006). Antimicrobial activity of extracts of chemical races of the lichen *Pseudevernia furfuracea* and their physodic acid, chloroatranorin, atranorin, and olivetoric acid constituents. Zeitschrift Für Naturforschung C.

[CR165] Ujat AH, Vadamalai G, Hattori Y, Nakashima C, Wong CKF, Zulperi D (2021). Current classification and diversity of *Fusarium* species complex, the causal pathogen of *Fusarium* wilt disease of banana in Malaysia. Agronomy.

[CR166] Ullah S, Khalil AA, Shaukat F, Song Y (2019). Sources, extraction and biomedical properties of polysaccharides. Foods.

[CR167] Valadbeigi T, Shaddel M (2015). Antibacterial and antifungal activities of gelatinose and non-gelatinose lichen species. J Arch Mil Med.

[CR168] Valadbeigi T, Bahrami AM, Shaddel M (2014). Antibacterial and antifungal activities of different lichens extracts. J Med Microbiol Infect Dis.

[CR169] Veres K, Farkas E, Csintalan Z (2020). The bright and shaded side of duneland life: the photosynthetic response of lichens to seasonal changes is species-specific. Mycol Prog.

[CR170] Vinayaka KS, Prashith Kekuda TR, Noor Nawaz AS, Syed J, Dileep N, Rakesh KN (2014). Inhibitory activity of *Usnea pictoides* G. Awasthi (Parmeliaceae) against *Fusarium oxysporum* f. sp. *zingiberi* and *Pythium aphanidermatum* isolated from rhizome rot of ginger. Life Sci Leafl.

[CR171] Voicu DMF, Mitoi ME, Gavriloaie C, Helepciuc FE, Toma N (2019). Chemical investigations of lichen biomass in *Usnea barbata*, *Cetraria islandica*, and *Xanthoria parietina* species. ELBA Bioflux.

[CR172] Vos C, McKinney P, Pearson C, Heiny E, Gunawardena G, Holt EA (2018). The optimal extraction and stability of atranorin from lichens, in relation to solvent and pH. Lichenologist.

[CR173] Wang YY, Liu B, Zhang X-Y, Zhou Q-M, Zhang T, Li H, Yu Y-F, Zhang X-L, Hao X-Y, Wang M, Wang M, Wang L, Wei J-C (2014). Genome characteristics reveal the impact of lichenization of lichen-forming fungus *Endocarpon pusillum* Hedwig (Verrucariales, Ascomycota). BMC Genom.

[CR174] Witaszak N, Stępień Ł, Bocianowski J, Waśkiewicz A (2019). *Fusarium* species and mycotoxins contaminating veterinary diets for dogs and cats. Microorganisms.

[CR175] Wolny-Koładka K. (2016). The presence of airborne *Fusarium culmorum* and its resistance to antimycotics – hypothetical risk to human health. [Obecność w powietrzu atmosferycznym grzybów z gatunku *Fusarium culmorum* i ich oporność na antymikotyki – hipotetyczne zagrożenie dla zdrowia ludzi]. Medycyna Środowiskowa, 19(1):33–36. [in Polish]

[CR176] Yadav H, Nayaka S, Dwivedi M (2021). Analytics on antimicrobial activity of lichen extract. J Pure Appl Microbiol.

[CR177] Yazıcı K, Aslan A, Aptroot A, Etayo J, Karahan D, Sipman H (2020). Lichens and lichenicolous fungi from Bitlis province in Turkey. Lindbergia.

[CR178] Yılmaz M, Türk AÖ, Tay T, Kıvanç M (2004). The antimicrobial activity of extracts of the lichen *Cladonia foliacea* and its (-)-usnic acid, atranorin and fumarprotocetraric acid constituents. Zeitschrift Für Naturforschung C.

[CR179] Yücel O, Odabasoğlu F, Güllüce M, Çalik ZZ, Aslan A, Yazici K, Halici M (2007). Antioxidant and antimicrobial properties of a lichen species, *Cladonia rangiformis* growing in Turkey. Turkish Journal of Pharmaceutical Sciences.

[CR180] Zagoskina NV, Nikolaeva TN, Lapshin PV, Zavarzin AA, Zavarzina AG (2013). Water-soluble phenolic compounds in lichens. Microbiology.

[CR181] Zavarzina AG, Nikolaeva TN, Demin VV, Lapshin PV, Makarov MI, Zavarzin AA, Zagoskina NV (2019). Water-soluble phenolic metabolites in lichens and their potential role in soil organic matter formation at the pre-vascular stage. Eur J Soil Sci.

[CR182] Zhang B-W, Xu J-L, Zhang H, Zhang Q, Lu J, Wang J-H (2016). Structure elucidation of a polysaccharide from *Umbilicaria esculenta* and its immunostimulatory activity. PLoS One.

[CR183] Zhao Y, Wang M, Xu B (2021). A comprehensive review on secondary metabolites and health-promoting effects of edible lichen. J Funct Foods.

[CR184] Zizovic I, Ivanovic J, Misic D, Stamenic M, Djordjevic S, Kukic-Markovic J, Petrovic SD (2012). SFE as a superior technique for isolation of extracts with strong antibacterial activities from lichen *Usnea barbata* L. J Supercrit Fluids.

