# Supplementary material for: A review of the potential of lichen substances as antifungal agents: the effects of extracts and lichen secondary metabolites on Fusarium fungi
Source: Arch Microbiol. 2022 Jul 26;204(8):523. doi: 10.1007/s00203-022-03104-4 (PMC9325835; doi:10.1007/s00203-022-03104-4)
Supplement: Supplementary file 1 — Supplementary file1 (DOCX 301 KB) [file 203_2022_3104_MOESM1_ESM.docx]

**Table S1.** Lichen species assigned to the main ecological groups tested on their antifungal potential against *Fusarium* spp. in the form of lichen extracts.

| **Ecological group** | **Lichen species** |
| --- | --- |
| **Corticolous lichens** | *Parmotrema andinum* (Müll. Arg.) Hale |
|  | *Alectoria sarmentosa* (Ach.) Ach. |
|  | *Anaptychia ciliaris* (L.) Körb. ex A. Massal. |
|  | *Bryoria capillaris* (Ach.) Brodo & D. Hawksw. |
|  | *Bulbothrix setschwanensis* (Zahlbr.) Hale |
|  | *Cetrelia braunsiana* (Müll. Arg.) W.L. Culb. & C.F. Culb. |
|  | *Dolichousnea longissima* (Ach.) Articus |
|  | *Evernia divaricata* (L.) Ach. |
|  | *Evernia prunastri* (L.) Ach. |
|  | *Flavoparmelia caperata* (L.) Hale  [= *Parmelia caperata* (L). Ach.] |
|  | *Heterodermia diademata* (Taylor) D.D. Awasthi |
|  | *Hypogymnia physodes* (L.) Nyl. |
|  | *Hypotrachyna cirrhata* (Fr.) Divakar, A. Crespo, Sipman, Elix & Lumbsch  [= *Parmelia cirrhata* Fr.] |
|  | *Hypotrachyna nepalensis* (Taylor) Divakar, A. Crespo, Sipman, Elix & Lumbsch  [= *Everniastrum nepalense* (Taylor) Hale ex Sipman] |
|  | *Leucodermia boryi* (Fée) Kalb  [=*Heterodermia boryi* (Fée) Kr.P. Singh & S.R. Singh] |
|  | *Leucodermia leucomelos* (L.) Kalb.  [= *Heterodermia leucomelos* (L.) Poelt] |
|  | *Melanelixia fuliginosa* (Fr. ex Duby) O. Blanco, A. Crespo, Divakar, Essl., D. Hawksw. & Lumbsch  [= *Melanelia fuliginosa* (Fr. ex Duby) Essl.] |
|  | *Melanelixia subaurifera* (Nyl.) O. Blanco, A. Crespo, Divakar, Essl., D. Hawksw. & Lumbsch  [= *Melanelia subaurifera* (Nyl.) Essl.] |
|  | *Menegazzia terebrata* (Hoffm.) A. Massal.  [= *Parmelia pertusa* Schaer.] |
|  | *Nephroma parile* (Ach.) Ach. |
|  | *Ochrolechia androgyna* (Hoffm.) Arnold |
|  | *Parmelia omphalodes* (L.) Ach. |
|  | *Parmelia saxatilis* (L.) Ach. |
|  | *Parmelia sulcata* Taylor |
|  | *Parmeliopsis ambigua* (Hoffm.) Nyl. |
|  | *Parmeliopsis hyperopta* (Ach.) Vain. |
|  | *Parmotrema austrosinense* (Zahlbr.) Hale |
|  | *Parmotrema crinitum* (Ach.) M. Choisy  [= *Parmelia crinita* Ach.] |
|  | *Parmotrema grayanum* (Hue) Hale |
|  | *Parmotrema perlatum* (Huds.) M. Choisy  [= *Parmelia perlata* (Huds.) Ach.] |
|  | *Parmotrema thomsonii* (Stirt.) A. Crespo, Divakar & Elix  [= *Parmelaria thomsonii* (Stirt.) D.D. Awasthi] |
|  | *Parmotrema tinctorum* (Despr. ex Nyl.) Hale |
|  | *Parmotrema reticulatum* (Taylor) M. Choisy  [= *Parmelia reticulata* Taylor] |
|  | *Physcia aipolia* (Ehrh. ex Humb.) Fürnr. |
|  | *Physcia caesia* (Hoffm.) Fürnr. |
|  | *Platismatia glauca* (L.) W.L. Culb. & C.F. Culb. |
|  | *Pseudevernia furfuracea* (L.) Zopf  [=*P. furfuracea* var. *ceratea* (Ach.) D. Hawksw.] |
|  | *Ramalina farinacea* (L.) Ach. |
|  | *Ramalina fastigiata* (Pers.) Ach. |
|  | *Ramalina fraxinea* (L.) Ach. |
|  | *Ramalina nervulosa* (Müll. Arg.) Abbayes |
|  | *Ramalina pollinaria* (Westr.) Ach. |
|  | *Ramalina polymorpha* (Lilj.) Ach. |
|  | *Ramalina roesleri* (Schaer.) Nyl. |
|  | *Roccella montagnei* Bél. |
|  | *Sticta weigelii* (Ach.) Vain. |
|  | *Teloschistes flavicans* (Sw.) Norman |
|  | *Usnea dasopoga* (Ach.) Nyl. |
|  | *Usnea florida* (L.) F.H. Wigg. |
|  | *Usnea pictoides* G. Awasthi |
|  | *Vulpicida pinastri* (Scop.) J.-E. Mattsson & M.J. Lai  [= *Cetraria pinastri* (Scop.) Gray] |
| **Terricolous lichens** | *Cetraria aculeata* (Schreb.) Fr. |
|  | *Catapyrenium squamulosum* (Ach.) Breuss  [=*Placidium squamulosum* (Ach.) Breuss] |
|  | *Cetraria islandica* (L.) Ach. |
|  | *Cladonia digitata* (L.) Hoffm. |
|  | *Cladonia fimbriata* (L.) Fr. |
|  | *Cladonia foliacea* (Huds.) Willd. |
|  | *Cladonia furcata* (Huds.) Schrad. |
|  | *Cladonia mitis* Sandst. |
|  | *Cladonia portentosa* (Dufour) Coem. |
|  | *Cladonia rangiferina* (L.) Weber ex. F.H. Wigg. |
|  | *Cladonia rangiformis* Hoffm. |
|  | *Cladonia subulata* (L.) Weber ex F.H. Wigg. |
|  | *Gyalolechia fulgens* (Sw.) Søchting, Frödén &Arup  [=*Fulgensia fulgens* (Sw.) Elenkin] |
|  | *Gyalolechia subbracteata* (Nyl.) Søchting, Frödén & Arup  [=*Fulgensia subbracteata* (Nyl.) Poelt] |
|  | *Psora decipiens* (Hedw.) Hoffm. |
|  | *Stereocaulon himalayense* D.D. Awasthi & I.M. Lamb |
|  | *Stereocaulon paschale* (L.) Hoffm. |
| **Saxicolous lichens** | *Arctoparmelia centrifuga* (L.) Hale  [= *Parmelia centrifuga* (L.) Ach.] |
|  | *Aspicilia cinerea* (L.) Körb. |
|  | *Blennothallia crispa* (Huds.) Otálora, P.M. Jørg. & Wedin  [=*Collema crispum* (Huds.) Weber ex F.H. Wigg.] |
|  | *Dermatocarpon miniatum* (L.) W. Mann |
|  | *Dermatocarpon vellereum* Zschacke |
|  | *Lasallia pustulata* (L.) Mérat |
|  | *Lathagrium cristatum* (L.) Otálora, P.M. Jørg. & Wedin  [= *Collema cristatum* (L.) Weber ex F.H. Wigg.] |
|  | *Lecanora frustulosa* (Dicks.) Ach. |
|  | *Ochrolechia parella* (L.) A. Massal. |
|  | *Ochrolechia tartarea* (L.) A. Massal. |
|  | *Protoparmeliopsis muralis* (Schreb.) M. Choisy  [= *Lecanora muralis* (Schreb). Rabenh.] |
|  | *Pyrenodesmia variabilis* (Pers.) A. Massal.  [=*Caloplaca variabilis* (Pers.) Müll. Arg.] |
|  | *Tephromela atra* (Huds.) Hafellner  [=*Lecanora atra* (Huds.) Ach.] |
|  | *Umbilicaria crustulosa* (Ach.) Lamy |
|  | *Umbilicaria cylindrica* (L.) Delise |
|  | *Umbilicaria nylanderiana* (Zahlbr.) H. Magn. |
|  | *Umbilicaria polyphylla* (L.) Baumg. |
|  | *Xanthoparmelia pulla* (Ach.) O. Blanco, A. Crespo, Elix, D. Hawksw. & Lumbsch  [= *Neofuscelia pulla* (Ach.) Essl.] |

**Table S2.** Biochemical classes of lichen secondary metabolites used against species of *Fusarium*. Unless otherwise stated, the names of the biochemical classes of secondary metabolites are given according to Elix (2014).

| **Biochemical classes of lichen secondary metabolites** | **Secondary metabolites** |
| --- | --- |
| aliphatic acids | protolichesterinic acid |
| benzyl esters | barbatolic acid |
| monocyclic aromatic derivatives | ethyl hematommate  ethyl orsellinate  methyl haematommate  methyl orsellinate  methyl β-orcinol carboxylate (evernyl**^†^**)  orsellinic acid |
| orcinol depsides | divaricatic acid  erythrin  homosekikaic acid  lecanoric acid  methyl evernate  olivetoric acid  sekikaic acid  2'-O-methylanziaic acid |
| orcinol β-orcinol depsides | obtusatic acid |
| β-orcinol depsides | atranorin  baeomycesic acid  chloroatranorin  diffractaic acid  thamnolic acid  2-hydroxy-4-methoxy-3,6-dimethylbenzoic acid^††^ |
| orcinol depsidones | lobaric acid  physodic acid |
| β-orcinol depsidones | fumarprotocetraric acid  norstictic acid  protocetraric acid  salazinic acid  stictic acid |
| orcinol tridepsides | gyrophoric acid |
| pulvinic acid derivatives | vulpinic acid |
| terpenoids | hopane-6α, 22-diol (zeorin) |
| usnic acid derivatives | isousnic acid  usnic acid  (+)-usnic acid |
| xanthones | lichexanthone |

**^†^** synonym: [www.pubchem.com](http://www.pubchem.com) [date: 06.11.2021]

**^††^** depside: Aravind et al. 2014

**Table S3.** Effect of crude extracts from corticolous, terricolous and saxicolous lichens on *Fusarium acuminatum*. Method of measurement abbreviations: BMM = Broth microdilution method (MIC); DDM = Disk diffusion method (IZ); MIC = Minimal Inhibitory Concentration; MFC = Minimal Fungicidal Concentration;
IZ = Inhibition Zone. Literature abbreviations: [1] Aslan et al. (2006); [2] Gulluce et al. (2006); [3] Yücel
et al. (2007).

| **Ecological group** | **Lichen species** | **Extracting solvent** | **Results** | | | **Measurement method** | **Literature** |
| --- | --- | --- | --- | --- | --- | --- | --- |
|  |  |  | **MIC**  **[**mg·ml^-1^] | **MFC**  **[**mg·ml^-1^] | **IZ** [mm]  (dose) |  |  |
| corticolous lichens | *Evernia divaricata* | methanol | 62.5 × 10^-3^ | ‡ | 30  (300 µg/disk)  (conc. 30 mg·ml^-1^) | BMM; DDM | [1] |
|  | *Evernia prunastri* | methanol | ‡ | ‡ | † | DDM | [1] |
|  | *Parmelia saxatilis* | methanol | ‡ | ‡ | † | DDM | [2] |
|  | *Platismatia glauca* | methanol | ‡ | ‡ | † | DDM | [2] |
|  | *Ramalina pollinaria* | methanol | ‡ | ‡ | † | DDM | [2] |
|  | *Ramalina polymorpha* | methanol | ‡ | ‡ | † | DDM | [2] |
| terricolous lichens | *Cladonia foliacea* | methanol | ‡ | ‡ | † | DDM | [1] |
|  | *Cladonia rangiformis* | chloroform | ‡ | ‡ | † | DDM | [3] |
|  |  | methanol | ‡ | ‡ | † |  |  |
|  |  | water | ‡ | ‡ | † |  |  |
| saxicolous lichens | *Dermatocarpon miniatum* | methanol | ‡ | ‡ | † | DDM | [1] |
|  | *Xanthoparmelia pulla* | methanol | ‡ | ‡ | † | DDM | [1] |
|  | *Umbilicaria nylanderiana* | methanol | ‡ | ‡ | † | DDM | [2] |

† no effect, ‡ not investigated

**Table S4.** Effect of crude extracts from two corticolous and three terricolous lichens on *Fusarium avenaceum*. Method of measurement abbreviations: PFT = Poisoned Food Technique. Literature abbreviations: [1] Tekiela
et al. (2021).

| **Lichen species** | **Extracting solvent** | **Results** | **Measurement method** | **Literature** |
| --- | --- | --- | --- | --- |
|  |  | **Average mycelium diameter***  [%] (dose) |  |  |
| *Cladonia mitis* | acetone | 6.6 (1 ml) | PFT | [1] |
| *Cladonia rangiferina* |  | 16.2 (1 ml) |  |  |
| joint *Cladonia mitis  + Cladonia rangiferina* | ethanol | 13.7 (1 ml) |  |  |
| joint *Cetraria islandica* + *Pseudevernia furfuracea* |  | 79.5 (1 ml) |  |  |
| joint *Pseudevernia furfuracea  + Usnea dasopoga* |  | 54.7 (1 ml) |  |  |
| joint *Cetraria islandica*  + *Pseudevernia furfuracea*  + *Usnea dasopoga* |  | 59.9 (1 ml) |  |  |

* The lichen inhibition potential is expressed as a percentage of the mean diameter of the mycelium in the control test after 180 h of experiment (Tekiela et al. 2021)

**Table S5.** Effect of crude extracts from corticolous and terricolous lichens on *Fusarium culmorum.* Method
of measurement abbreviations: DDM = Disk diffusion method (MIC; IZ); PFT = Poisoned Food Technique (IR%); IR% = percentage fungus inhibition rate. For MIC, MFC and IZ abbreviations: see Tab. S3. Literature abbreviations: [1] Karabulut and Ozturk (2015); [2] Türk et al. (2006); [3] Türk et al. (2003); [4] Yılmaz
et al. (2004).

| **Ecological group** | **Lichen species** | **Extracting solvent** | **Results** | | | | **Measurement method** | **Literature** |
| --- | --- | --- | --- | --- | --- | --- | --- | --- |
|  |  |  | **MIC**  **[**mg·ml^-1^] | **MFC**  **[**mg·ml^-1^] | **IZ** [mm]  (dose) | **Others** |  |  |
| corticolous lichens | *Evernia prunastri* | ethanol | ‡ | ‡ | ‡ | IR%: 100 (375 µl) (extract conc.: 10%) | PFT | [1] |
|  | *Parmelia sulcata* | ethanol | ‡ | ‡ | ‡ | IR%: 67.51 (375 µl) (extract conc.: 10%) | PFT | [1] |
|  | *Pseudevernia furfuracea* | acetone  (test sample  1 & 2) | 320 µg/disk  (conc. 6 mg·ml^-1^) | ‡ | ‡ | ‡ | DDM | [2] |
|  |  |  | 597 µg/disk  (conc. 5.6 mg·ml^-1^) | ‡ | ‡ | ‡ |  |  |
|  |  | chloroform  (test sample  1 & 2) | 787 µg/disk  (conc. 7.4 mg·ml^-1^) | ‡ | ‡ | ‡ | DDM | [2] |
|  |  |  | 1013 µg/disk  (conc. 8.5 mg·ml^-1^) | ‡ | ‡ | ‡ |  |  |
|  |  | ethanol  (test sample  1 & 2) | 800 µg/disk  (conc. 15 mg·ml^-1^) | ‡ | ‡ | ‡ | DDM | [2] |
|  |  |  | 1460 µg/disk  (conc. 6.8 mg·ml^-1^) | ‡ | ‡ | ‡ |  |  |
|  |  |  | ‡ | ‡ | ‡ | IR%: 42.72 (375 µl) (extract conc.: 10%) | PFT | [1] |
| terricolous lichens | *Cetraria aculeata* | acetone | ‡ | ‡ | † | ‡ | DDM | [3] |
|  |  | diethyl ether | ‡ | ‡ | † | ‡ |  |  |
|  |  | ethanol | ‡ | ‡ | † | ‡ |  |  |
|  | *Cladonia foliacea* | acetone | † | ‡ | ‡ | ‡ | DDM | [4] |
|  |  | chloroform | † | ‡ | ‡ | ‡ |  |  |
|  |  | diethyl ether | † | ‡ | ‡ | ‡ |  |  |
|  |  | ethanol | † | ‡ | ‡ | ‡ |  |  |
|  |  | petroleum ether | † | ‡ | ‡ | ‡ |  |  |

† no effect, ‡ not investigated

**Table S6.** Effect of crude extracts from corticolous lichens on *Fusarium oxysporum.* Method of measurement abbreviations: BMM = Broth microdilution method (MIC); BMMwTTC = Broth microdilution method with 2, 3, 5-triphenyltetrazolium chloride (MIC); BTDM = Broth tube dilution method (MIC); DDM= Disk diffusion method (IZ, IR%); MMwR = Microdilution method with resazurin (MIC); AWDM = Agar well diffusion method (IZ); SGI = Spore Germination Inhibition; PFT = Poisoned Food Technique (ED_50_, IG%);
ED_50_ = Effective Dose required for 50% growth inhibition; IG% = percentage growth inhibition of mycelium compared to the diameter of mycelium in control sample; IR% = percentage fungus inhibition rate. For MIC, MFC and IZ abbreviations: see Tab. S3. Literature abbreviations: [1] Ranković and Mišić (2007); [2] Ranković
et al. (2010a); [3] Tiwari et al. (2011a); [4] Yadav et al. (2021); [5] Aslan et al. (2006); [6] Shivanna
and Garampalli (2014); [7] Babiah et al. (2014a); [8] Ranković et al. (2009); [9] Kosanić et al. (2012a); [10] Kosanić and Ranković (2011a); [11] Kosanić et al. (2013); [12] Shahi et al. (2003); [13] Balasubramanian
and Nirmala (2014); [14] Shahi et al. (2001); [15] Ristić et al. (2016b); [16] Ranković et al. (2007a);
[17] Kosanić and Ranković (2011b); [18] Ranković and Kosanić (2012); [19] Gulluce et al. (2006);
[20] Ranković et al. (2007b); [21] Anjali et al. (2015a); [22] Ranković et al. (2010c); [23] Kosanić et al. (2010); [24] Ranković et al. (2010b); [25] Thippeswamy et al. (2013); [26] Babiah et al. (2014b); [27] Goel
et al. (2011a); [28] Prashith Kekuda et al. (2015); [29] Tiwari et al. (2011b); [30] Anjali et al. (2015b); [31] Türk et al. (2006); [32] Gazo et al. (2019); [33] Ristić et al. (2016a); [34] Devashree et al. (2019); [35] Vinayaka
et al. (2014).

| **Lichen species** | **Extracting solvent** | **Results** | | | | **Measurement method** | **Literature** |
| --- | --- | --- | --- | --- | --- | --- | --- |
|  |  | **MIC**  **[**mg·ml^-1^] | **MFC**  **[**mg·ml^-1^] | **IZ** [mm]  (dose) | **Others** |  |  |
| *Alectoria sarmentosa* | ethanol | 50 | 100 | 18  (20 µl/well) | ‡ | BTDM; AWDM | [1] |
|  | ethyl acetate | 100 | 150 | 15  (20 µl/well) | ‡ |  |  |
|  | water | >200 | ‡ | † | ‡ |  |  |
| *Anaptychia ciliaris* | methanol | † | ‡ | † | ‡ | BMM; DDM | [2] |
| *Bulbothrix setschwanensis* | acetone | ‡ | ‡ | 10 (5 ml)  (conc. 50 mg·ml^-1^) | ‡ | DDM | [3] |
|  | chloroform | ‡ | ‡ | c. 12 (5 ml)  (conc. 50 mg·ml^-1^) | ‡ |  |  |
|  | methanol | ‡ | ‡ | c. 6 (5 ml)  (conc. 50 mg·ml^-1^) | ‡ |  |  |
| *Cetrelia braunsiana* | acetone | ‡ | ‡ | 22  (extract conc.: 20 mg·ml^-1^) | ‡ | AWDM | [4] |
|  | ethanol | ‡ | ‡ | 25  (extract conc.: 20 mg·ml^-1^) | ‡ |  |  |
|  | ethyl acetate | ‡ | ‡ | 24  (extract conc.: 20 mg·ml^-1^) | ‡ |  |  |
|  | methanol | ‡ | ‡ | 22  (extract conc.: 20 mg·ml^-1^) | ‡ |  |  |
| *Dolichousnea longissima* | acetone | ‡ | ‡ | 10  (extract conc.: 20 mg·ml^-1^) | ‡ | AWDM | [4] |
|  | ethanol | ‡ | ‡ | 12  (extract conc.: 20 mg·ml^-1^) | ‡ |  |  |
|  | ethyl acetate | ‡ | ‡ | 12  (extract conc.: 20 mg·ml^-1^) | ‡ |  |  |
|  | methanol | ‡ | ‡ | 14  (extract conc.: 20 mg·ml^-1^) | ‡ |  |  |
| *Evernia divaricata* | methanol | ‡ | ‡ | † | ‡ | DDM | [5] |
| *Evernia prunastri* | methanol | ‡ | ‡ | † | ‡ | DDM | [5] |
| *Flavoparmelia caperata* | acetone | 1.562 | ‡ | 13  (100 µl/well)  (30 mg·ml^-1^) | ‡ | BMMwTTC; AWDM | [6] |
|  |  | 6.25 | ‡ | 11.6  (50 µl) | ‡ | BTDM; DDM | [7] |
|  |  | 1.56 | ‡ | 21  (15 µl)  (conc. 50 mg·ml^-1^) | ‡ | BTDM; DDM | [8] |
|  | acetone | 25 | ‡ | ‡ | ‡ | MMwR | [9] |
|  | chloroform | 6.25 | ‡ | 7.6  (50 µl) | ‡ | BTDM; DDM | [7] |
|  | ethyl acetate | 1.875 | ‡ | 18.3  (100 µl/well) (30 mg·ml^-1^) | ‡ | BMMwTTC; AWDM | [6] |
|  | methanol | 0.097 | ‡ | 13.3  (100 µl/well) (30 mg·ml^-1^) | ‡ | BMMwTTC; AWDM | [6] |
|  |  | 6.25 | ‡ | 9.3  (50 µl) | ‡ | BTDM; DDM | [7] |
|  |  | 1.56 | ‡ | 21  (15 µl)  (conc. 50 mg·ml^-1^) | ‡ | BTDM; DDM | [8] |
|  | water | † | ‡ | † | ‡ | BTDM; DDM | [8] |
| *Heterodermia diademata* | acetone | ‡ | ‡ | c. 11  (5 ml)  (conc. 50 mg·ml^-1^) | ‡ | DDM | [3] |
|  | chloroform | ‡ | ‡ | † | ‡ |  |  |
|  | methanol | ‡ | ‡ | c. 12  (5 ml)  (conc. 50 mg·ml^-1^) | ‡ |  |  |
| *Hypogymnia physodes* | acetone | 12.5 | ‡ | 19  (15 µl)  (conc. 50 mg·ml^-1^) | ‡ | BTDM; DDM | [8] |
|  |  | 12.5 | ‡ | ‡ | ‡ | BMM | [10] |
|  |  | 12.5 | ‡ | ‡ | ‡ | MMwR | [11] |
|  | methanol | 6.25 | ‡ | 16  (15 µl)  (conc. 50 mg·ml^-1^) | ‡ | BTDM; DDM | [8] |
|  |  | 6.25 | ‡ | ‡ | ‡ | BMM | [10] |
|  | water | † | ‡ | † | ‡ | BTDM; DDM | [8] |
|  |  | † | ‡ | ‡ | ‡ | BMM | [10] |
| *Hypotrachyna cirrhata* | water | ‡ | ‡ | ‡ | SGI: 100%  (80 µl·ml^-1^) (fungicidal) | SGI | [12] |
| *Hypotrachyna nepalensis* | acetone | ‡ | ‡ | c. 11 (5 ml)  (conc. 50 mg·ml^-1^) | ‡ | DDM | [3] |
|  | chloroform | ‡ | ‡ | † | ‡ |  |  |
|  | methanol | ‡ | ‡ | 15 (5 ml)  (conc. 50 mg·ml^-1^) | ‡ |  |  |
| *Leucodermia boryi* | acetone | ‡ | ‡ | 12  (20 µl/well) (conc. 50 mg·ml^-1^) | ‡ | AWDM | [13] |
|  | chloroform | ‡ | ‡ | 6  (20 µl/well) (conc. 50 mg·ml^-1^) | ‡ |  |  |
|  | diethyl ether | ‡ | ‡ | † | ‡ |  |  |
|  | ethanol | ‡ | ‡ | 21  (20 µl/well) (conc. 50 mg·ml^-1^) | ‡ |  |  |
|  | ethyl acetate | ‡ | ‡ | 20  (20 µl/well) (conc. 50 mg·ml^-1^) | ‡ |  |  |
|  | hexane | ‡ | ‡ | 14  (20 µl/well) (conc. 50 mg·ml^-1^) | ‡ |  |  |
|  | methanol | ‡ | ‡ | † | ‡ |  |  |
| *Leucodermia leucomelos* | water | ‡ | ‡ | ‡ | SGI: 100%  (80 µl·ml^-1^) (fungicidal) | SGI | [14] |
| *Melanelixia fuliginosa* | acetone | 2.5 | ‡ | ‡ | ‡ | MMwR | [15] |
| *Melanelixia subaurifera* | acetone | 10 | ‡ | ‡ | ‡ | MMwR | [15] |
| *Menegazzia terebrata* | acetone | 1.56 | ‡ | 21  (15 µl)  (conc. 50 mg·ml^-1^) | ‡ | BTDM; DDM | [8] |
|  |  | 6.25 | ‡ | ‡ | ‡ | MMwR | [11] |
|  | methanol | 1.56 | ‡ | 21  (15 µl)  (conc. 50 mg·ml^-1^) | ‡ | BTDM; DDM | [8] |
|  | water | † | ‡ | † | ‡ | BTDM; DDM | [8] |
| *Nephroma parile* | methanol | † | ‡ | † | ‡ | BMM; DDM | [2] |
| *Ochrolechia androgyna* | acetone | 3.12 | ‡ | 20  (15 µl)  (conc. 50 mg·ml^-1^) | ‡ | BTDM; DDM | [16] |
|  | methanol | 50 | ‡ | 9  (15 µl)  (conc. 50 mg·ml^-1^) | ‡ |  |  |
|  | water | † | ‡ | † | ‡ |  |  |
| *Parmelia omphalodes* | acetone | 3.12 | ‡ | 15  (15 µl)  (conc. 50 mg·ml^-1^) | ‡ | BTDM; DDM | [17] |
|  | methanol | 3.12 | ‡ | 18  (15 µl)  (conc. 50 mg·ml^-1^) | ‡ |  |  |
|  | water | † | ‡ | † | ‡ |  |  |
| *Parmelia saxatilis* | acetone | 25 | ‡ | ‡ | ‡ | MMwR | [9] |
|  |  | 25 | ‡ | 13  (15 µl)  (conc. 50 mg·ml^-1^) | ‡ | BTDM; DDM | [18] |
|  | methanol | 12.5 | ‡ | 17  (15 µl)  (conc. 50 mg·ml^-1^) | ‡ | BTDM; DDM | [18] |
|  |  | ‡ | ‡ | † | ‡ | DDM | [19] |
|  | water | † | ‡ | † | ‡ | BTDM; DDM | [18] |
| *Parmelia sulcata* | acetone | 1.56 | ‡ | ‡ | ‡ | MMwR | [9] |
|  |  | 25 | ‡ | 12  (15 µl) | ‡ | BTDM; DDM | [20] |
|  |  | 6.25 | ‡ | 14  (15 µl)  (conc. 50 mg·ml^-1^) | ‡ | BTDM; DDM | [18] |
|  | methanol | 25 | ‡ | 11  (15 µl) | ‡ | BTDM; DDM | [20] |
|  |  | 3.12 | ‡ | 16  (15 µl)  (conc. 50 mg·ml^-1^) | ‡ | BTDM; DDM | [18] |
|  | water | † | ‡ | † | ‡ | BTDM; DDM | [20] |
|  |  | † | ‡ | † | ‡ | BTDM; DDM | [18] |
| *Parmeliopsis ambigua* | acetone | 25 | ‡ | ‡ | ‡ | MMwR | [11] |
|  |  | 25 | ‡ | 12  (15 µl)  (conc. 50 mg·ml^-1^) | ‡ | BTDM; DDM | [18] |
|  | methanol | 6.25 | ‡ | 17  (15 µl)  (conc. 50 mg·ml^-1^) | ‡ | BTDM; DDM | [18] |
|  | water | † | ‡ | † | ‡ | BTDM; DDM | [18] |
| *Parmotrema andinum* | acetone | ‡ | ‡ | 5.33  (15 µl)  (conc.  200 mg·2 ml^-1^) | ‡ | DDM | [21] |
|  | 2-propanol | ‡ | ‡ | 19.66  (15 µl)  (conc.  200 mg·2 ml^-1^) | ‡ |  |  |
| *Parmotrema austrosinense* | acetone | 1.562 | ‡ | 12.3  (100 µl/well) (30 mg·ml^-1^) | ‡ | BMMwTTC; AWDM | [6] |
|  | ethyl acetate | 3.125 | ‡ | 14.6  (100 µl/well) (30 mg·ml^-1^) | ‡ |  |  |
|  | methanol | 0.39 | ‡ | 21.3  (100 µl/well) (30 mg·ml^-1^) | ‡ |  |  |
| *Parmotrema grayanum* | acetone | † | ‡ | † | ‡ | BMMwTTC; AWDM | [6] |
|  | ethyl acetate | 3.125 | ‡ | 11.6  (100 µl/well) (30 mg·ml^-1^) | ‡ |  |  |
|  | methanol | † | ‡ | † | ‡ |  |  |
| *Parmeliopsis hyperopta* | acetone | 12.5 | ‡ | 12  (15 µl)  (conc. 50 mg·ml^-1^) | ‡ | BTDM; DDM | [22] |
|  |  | 12.5 | ‡ | 12  (15 µl)  (conc. 50 mg·ml^-1^) | ‡ | BTDM; DDM | [23] |
|  | methanol | 3.12 | ‡ | 16  (15 µl)  (conc. 50 mg·ml^-1^) | ‡ | BTDM; DDM | [22] |
|  |  | 3.12 | ‡ | 16  (15 µl)  (conc. 50 mg·ml^-1^) | ‡ | BTDM; DDM | [23] |
|  | water | † | ‡ | † | ‡ | BTDM; DDM | [22] |
|  |  | † | ‡ | † | ‡ | BTDM; DDM | [23] |
| *Parmotrema crinitum* | methanol | 15 | ‡ | 12  (10 µl)  (300 µg/disk) (conc. 30 mg·ml^-1^) | ‡ | BTDM; DDM | [24] |
| *Parmotrema perlatum* | acetone | ‡ | ‡ | 30  (conc. 50 µg·µl^-1^) | ‡ | AWDM | [25] |
|  | chloroform | ‡ | ‡ | 20  (conc. 50 µg·µl^-1^) | ‡ |  |  |
|  | methanol | ‡ | ‡ | 11  (conc. 50 µg·µl^-1^) | ‡ |  |  |
|  |  | ‡ | ‡ | 26  (conc. 50 µg·µl^-1^) | ‡ |  |  |
|  | petroleum ether | ‡ | ‡ | 17  (conc. 50 µg·µl^-1^) | ‡ |  |  |
| *Parmotrema reticulatum* | acetone | 12.5 | ‡ | 15  (15 µl)  (conc. 50 mg·ml^-1^) | ‡ | BTDM; DDM | [17] |
|  |  | † | ‡ | ‡ | ‡ | BMMwTTC; AWDM | [6] |
|  |  | 50 | ‡ | 11.6  (50 µl) | IR%: 68 | BTDM; DDM | [26] |
|  | chloroform | 50 | ‡ | 8  (50 µl) | IR%: 47 | BTDM; DDM | [26] |
|  | dichloromethane (methylene chloride) | ‡ | ‡ | ‡ | ED_50_: 105.5 µg·ml^-1^ | PFT | [27] |
|  | ethyl acetate | ‡ | ‡ | ‡ | ED_50_: 43.7 µg·ml^-1^ | PFT | [27] |
|  |  | † | ‡ | ‡ | ‡ | BMMwTTC; AWDM | [6] |
|  | hexane | ‡ | ‡ | ‡ | ED_50_: 200.3 µg·ml^-1^ | PFT | [27] |
|  | methanol | 6.25 | ‡ | 19  (15 µl)  (conc. 50 mg·ml^-1^) | ‡ | BTDM; DDM | [17] |
|  |  | 50 | ‡ | 10  (50 µl) | IR%: 58 | BTDM; DMM | [26] |
|  |  | † | ‡ | ‡ | ‡ | BMMwTTC; AWDM | [6] |
|  |  | ‡ | ‡ | ‡ | ED_50_: 59.2 µg·ml^-1^ | PFT | [27] |
|  |  | ‡ | ‡ | ‡ | IG%: 39.13 | PFT | [28] |
|  | water | † | ‡ | † | ‡ | BTDM; DDM | [17] |
|  |  | ‡ | ‡ | ‡ | ED_50_: 213.4 µg·ml^-1^ | PFT | [27] |
| *Parmotrema tinctorum* | acetone | † | ‡ | † | ‡ | BMMwTTC; AWDM | [6] |
|  |  | ‡ | ‡ | 15.6  (5 ml) | ‡ | DDM | [29] |
|  | chloroform | ‡ | ‡ | 6.3  (5 ml) | ‡ | DDM | [29] |
|  | ethyl acetate | 3.125 | ‡ | 14.3  (100 µl/well)  (30 mg·ml^-1^) | ‡ | BMMwTTC; AWDM | [6] |
|  | methanol | 6.25 | ‡ | 11.3  (100 µl/well)  (30 mg·ml^-1^) | ‡ | BMMwTTC; AWDM | [6] |
|  |  | ‡ | ‡ | 17.6  (5 ml) | ‡ | DDM | [29] |
|  | 2-propanol | ‡ | ‡ | 7.33  (15 µl)  (conc.  100 mg·ml^-1^) | ‡ | DDM | [30] |
| *Parmotrema thomsonii* | acetone | ‡ | ‡ | c. 20  (5 ml)  (conc. 50 mg·ml^-1^) | ‡ | DDM | [3] |
|  | chloroform | ‡ | ‡ | c. 6  (5 ml)  (conc. 50 mg·ml^-1^) | ‡ | DDM | [3] |
|  | methanol | ‡ | ‡ | c. 7  (5 ml)  (conc. 50 mg·ml^-1^) | ‡ | DDM | [3] |
| *Physcia aipolia* | acetone | 6.25 | ‡ | 14  (15 µl)  (conc. 50 mg·ml^-1^) | ‡ | BTDM; DDM | [16] |
|  |  | † | ‡ | † | ‡ | BMMwTTC; AWDM | [6] |
|  | ethyl acetate | 0.39 | ‡ | 12.3  (100 µl/well)  (30 mg·ml^-1^) | ‡ | BMMwTTC; AWDM | [6] |
|  | methanol | 25 | ‡ | 12  (15 µl)  (conc. 50 mg·ml^-1^) | ‡ | BTDM; DDM | [16] |
|  |  | 0.39 | ‡ | 12.6  (100 µl/well)  (30 mg·ml^-1^) | ‡ | BMMwTTC; AWDM | [6] |
|  | water | † | ‡ | † | ‡ | BTDM; DDM | [16] |
| *Physcia caesia* | acetone | 6.25 | ‡ | 16  (15 µl)  (conc. 50 mg·ml^-1^) | ‡ | BTDM; DDM | [16] |
|  | methanol | 3.12 | ‡ | 18  (15 µl)  (conc. 50 mg·ml^-1^) | ‡ |  |  |
|  | water | † | ‡ | † | ‡ |  |  |
| *Platismatia glauca* | methanol | ‡ | ‡ | † | ‡ | DDM | [19] |
| *Pseudevernia furfuracea* | acetone | 597 µg/disk  (conc. 5.6 mg·ml^-1^) | ‡ | ‡ | ‡ | DDM | [31] |
|  |  | 640 µg/disk  (conc. 6 mg·ml^-1^) | ‡ | ‡ | ‡ | DDM | [31] |
|  | chloroform | 1574 µg/disk  (conc. 7.4 mg·ml^-1^) | ‡ | ‡ | ‡ | DDM | [31] |
|  |  | 1813 µg/disk  (conc. 8.5 mg·ml^-1^) | ‡ | ‡ | ‡ | DDM | [31] |
|  | ethanol | 800 µg/disk  (conc. 15 mg·ml^-1^) | ‡ | ‡ | ‡ | DDM | [31] |
|  |  | 1460 µg/disk  (conc. 6.8 mg·ml^-1^) | ‡ | ‡ | ‡ | DDM | [31] |
| *Ramalina faricacea* | acetone | ‡ | ‡ | † | ‡ | DDM | [32] |
| *Ramalina fastigiata* | acetone | 2.5 | ‡ | ‡ | ‡ | MMwR | [33] |
| *Ramalina fraxinea* | acetone | 5 | ‡ | ‡ | ‡ | MMwR | [33] |
| *Ramalina nervulosa* | acetone | ‡ | ‡ | † | ‡ | DDM | [32] |
| *Ramalina pollinaria* | methanol | ‡ | ‡ | † | ‡ | DDM | [19] |
| *Ramalina polymorpha* | methanol | ‡ | ‡ | † | ‡ | DDM | [19] |
| *Ramalina roesleri* | acetone | ‡ | ‡ | † | ‡ | DDM | [32] |
| *Roccella montagnei* | acetone | 3.125 | ‡ | 12.6  (100 µl/well)  (30 mg·ml^-1^) | ‡ | BMMwTTC; AWDM | [6] |
|  | acetone | ‡ | ‡ | 14 | ‡ | AWDM | [34] |
|  | ethanol | ‡ | ‡ | 32 | ‡ | AWDM | [34] |
|  | ethyl acetate | 0.39 | ‡ | 11.3  (100 µl/well)  (30 mg·ml^-1^) | ‡ | BMMwTTC; AWDM | [6] |
|  | ethyl acetate | ‡ | ‡ | 24 | ‡ | AWDM | [34] |
|  | methanol | 0.39 | ‡ | 13  (100 µl/well)  (30 mg·ml^-1^) | ‡ | BMMwTTC; AWDM | [6] |
|  | methanol | ‡ | ‡ | 25 | ‡ | AWDM | [34] |
| *Sticta weigelii* | acetone | ‡ | ‡ | 15  (20 µl/well)  (conc. 50 mg·ml^-1^) | ‡ | AWDM | [13] |
|  | chloroform | ‡ | ‡ | † | ‡ |  |  |
|  | diethyl ether | ‡ | ‡ | † | ‡ |  |  |
|  | ethanol | ‡ | ‡ | † | ‡ |  |  |
|  | ethyl acetate | ‡ | ‡ | 8  (20 µl/well)  (conc. 50 mg·ml^-1^) | ‡ |  |  |
|  | hexane | ‡ | ‡ | † | ‡ |  |  |
|  | methanol | ‡ | ‡ | † | ‡ |  |  |
| *Teloschistes flavicans* | acetone | † | ‡ | † | ‡ | BMMwTTC; AWDM | [6] |
|  | ethyl acetate | 6.25 | ‡ | 16.6  (100 µl/well)  (30 mg·ml^-1^) | ‡ |  |  |
|  | methanol | † | ‡ | † | ‡ |  |  |
| *Usnea pictoides*  (vs. *Fusarium oxysporum* f. sp*. zingiberi* E.E. Trujillo) | water | ‡ | ‡ | ‡ | IG%: c. 45  (extract conc.: 25%) | PFT | [35] |
| *Vulpicida pinastri* | methanol | 7.5 | ‡ | 18 (10 µl)  (300 µg/disk) (conc. 30 mg·ml^-1^) | ‡ | BTDM; DDM | [24] |

† no effect, ‡ not investigated

**Table S7.** Effect of crude extracts from terricolous lichens on *Fusarium oxysporum.* Method of measurement abbreviations: BMM = Broth microdilution method (MIC); BTDM = Broth tube dilution method (MIC);
DDM = Disk diffusion method (MIC, IZ); MMwR = Microdilution method with resazurin (MIC);
PFT = Poisoned Food Technique (ED_50_); ED_50_ = Effective Dose required for 50% growth inhibition. For MIC, MFC and IZ abbreviations: see Tab. S3. Literature abbreviations: [1] Türk et al. (2003); [2] Grujičić et al. (2014); [3] Ranković et al. (2010b); [4] Kosanić et al. (2018); [5] Yılmaz et al. (2004); [6] Aslan et al. (2006); [7] Ranković et al. (2009); [8] Ranković et al. (2011); [9] Kosanić and Ranković (2011a); [10] Tekiela et al. (2021); [11] Ranković and Mišić (2007); [12] Yücel et al. (2007); [13] Ranković et al. (2014); [14] Goel et al. (2011a).

| **Lichen species** | **Extracting solvent** | **Results** | | | | **Measurement method** | **Literature** |
| --- | --- | --- | --- | --- | --- | --- | --- |
|  |  | **MIC**  **[**mg·ml^-1^] | **MFC**  **[**mg·ml^-1^] | **IZ** [mm]  (dose) | **Others** (dose) |  |  |
| *Cetraria aculeata* | acetone | ‡ | ‡ | † | ‡ | DDM | [1] |
|  | diethyl ether | ‡ | ‡ | † | ‡ |  |  |
|  | ethanol | ‡ | ‡ | † | ‡ |  |  |
| *Cetraria islandica* | methanol | 2.5 | ‡ | ‡ | ‡ | MMwR | [2] |
| *Cladonia digitata* | methanol | † | ‡ | † | ‡ | BTDM; DDM | [3] |
| *Cladonia fimbriata* | acetone | 5 | ‡ | ‡ | ‡ | MMwR | [4] |
|  | methanol | † | ‡ | † | ‡ | BTDM; DDM | [3] |
| *Cladonia foliacea* | acetone | † | ‡ | ‡ | ‡ | DDM | [5] |
|  |  | 10 | ‡ | ‡ | ‡ | MMwR | [4] |
|  | chloroform | † | ‡ | ‡ | ‡ | DDM | [5] |
|  | diethyl ether | † | ‡ | ‡ | ‡ |  | [5] |
|  | ethanol | † | ‡ | ‡ | ‡ |  | [5] |
|  | methanol | ‡ | ‡ | † | ‡ |  | [6] |
|  | petroleum ether | † | ‡ | ‡ | ‡ |  | [5] |
| *Cladonia furcata* | acetone | 25 | ‡ | 11  (15 µl)  (conc. 50 mg·ml^-1^) | ‡ | BTDM; DDM | [7] |
|  |  | 25 | ‡ | ‡ | ‡ | BMM | [8] |
|  |  | 25 | ‡ | ‡ | ‡ | BMM | [9] |
|  |  | 10 | ‡ | ‡ | ‡ | MMwR | [4] |
|  | methanol | 25 | ‡ | 12  (15 µl)  (conc. 50 mg·ml^-1^) | ‡ | BTDM; DDM | [7] |
|  |  | 25 | ‡ | ‡ | ‡ | BMM | [9] |
|  | water | † | ‡ | † | ‡ | BTDM; DDM | [7] |
|  |  | † | ‡ | ‡ | ‡ | BTDM | [9] |
| *Cladonia mitis* | acetone | ‡ | ‡ | ‡ | 23.3 (1 ml)***** | PFT | [10] |
|  | ethanol | ‡ | ‡ | ‡ | 42.1 (1 ml)***** | PFT | [10] |
| joint *Cladonia mitis*  + *Cladonia rangiferina* | acetone | ‡ | ‡ | ‡ | 50.3 (1 ml)***** | PFT | [10] |
|  | ethanol | ‡ | ‡ | ‡ | 41.8 (1 ml)***** | PFT | [10] |
| *Cladonia rangiferina* | acetone | 10 | ‡ | ‡ | ‡ | MMwR | [4] |
|  |  | ‡ | ‡ | ‡ | 21.4 (1 ml)***** | PFT | [10] |
|  | ethanol | ‡ | ‡ | ‡ | 43.3 (1 ml)***** | PFT | [10] |
|  |  | 100 | 100 | 14  (20 µl/well) | ‡ | BTDM; AWDM | [11] |
|  | ethyl acetate | 150 | 150 | 13  (20 µl/well) | ‡ |  |  |
|  | water | >200 | ‡ | † | ‡ |  |  |
| *Cladonia rangiformis* | chloroform | ‡ | ‡ | † | ‡ | DDM | [12] |
|  | methanol | ‡ | ‡ | † | ‡ |  |  |
|  | water | ‡ | ‡ | † | ‡ |  |  |
| *Cladonia subulata* | acetone | 5 | ‡ | ‡ | ‡ | MMwR | [4] |
| *Gyalolechia fulgens* | methanol | 30 | ‡ | 12  (10 µl)  (300 µg/disk) (conc. 30 mg·ml^-1^) | ‡ | BTDM; DDM | [3] |
| *Stereocaulon paschale* | methanol | 5 | ‡ | ‡ | ‡ | MMwR | [13] |
| *Stereocaulon himalayense* | dichloromethane (methylene chloride) | ‡ | ‡ | ‡ | ED_50_: 72.23 µg·ml^-1^ | PFT | [14] |
|  | ethyl acetate | ‡ | ‡ | ‡ | ED_50_: 137.6 µg·ml^-1^ |  |  |
|  | hexane | ‡ | ‡ | ‡ | ED_50_: 66.54 µg·ml^-1^ |  |  |
|  | methanol | ‡ | ‡ | ‡ | ED_50_: 246.9 µg·ml^-1^ |  |  |
|  | water | ‡ | ‡ | ‡ | ED_50_: 293.2 µg·ml^-1^ |  |  |

† no effect, ‡ not investigated

* The lichen inhibition potential is expressed as a percentage of the mean diameter of the mycelium in the control test after 180 h of experiment
(Tekiela et al. 2021)

**Table S8.** Effect of crude extracts from saxicolous lichens on *Fusarium oxysporum.* Method of measurement abbreviations: BMM = Broth microdilution method (MIC); BTDM = Broth tube dilution method (MIC);
DDM = Disk diffusion method (IZ); MMwR = Microdilution method with resazurin (MIC); AWDM = Agar well diffusion method (IZ). For MIC, MFC and IZ abbreviations: see Tab. S3. Literature abbreviations:
[1] Ranković et al. (2010a); [2] Ranković et al. (2007a); [3] Aslan et al. (2006); [4] Balasubramanian
and Nirmala (2014); [5] Ranković et al. (2007b); [6] Kosanić et al. (2016); [7] Ranković et al. (2010c);
[8] Kosanić et al. (2010); [9] Ranković et al. (2010b); [10] Ranković and Kosanić (2012); [11] Ranković
et al. (2011); [12] Kosanić and Ranković (2011b); [13] Kosanić et al. (2012b); [14] Gulluce et al. (2006);
[15] Ranković et al. (2009); [16] Kosanić and Ranković (2011a).

| **Lichen species** | **Extracting solvent** | **Results** | | | **Measurement method** | **Literature** |
| --- | --- | --- | --- | --- | --- | --- |
|  |  | **MIC**  **[**mg·ml^-1^] | **MFC**  **[**mg·ml^-1^] | **IZ** [mm]  (dose) |  |  |
| *Arctoparmelia centrifuga* | methanol | 30 | ‡ | 8  (10 µl) (300 µg/disc)  (conc. 30 mg·ml^-1^) | BMM; DDM | [1] |
| *Aspicilia cinerea* | acetone | 25 | ‡ | 12  (15 µl)  (conc. 50 mg·ml^-1^) | BTDM; DDM | [2] |
|  | methanol | 25 | ‡ | 11  (15 µl)  (conc. 50 mg·ml^-1^) |  |  |
|  | water | † | ‡ | † |  |  |
| *Dermatocarpon miniatum* | methanol | ‡ | ‡ | † | DDM | [3] |
| *Dermatocarpon vellereum* | acetone | ‡ | ‡ | 10  (20 µl/well)  (conc. 50 mg·ml^-1^) | AWDM | [4] |
|  | chloroform | ‡ | ‡ | 18  (20 µl/well)  (conc. 50 mg·ml^-1^) | AWDM | [4] |
|  | diethyl ether | ‡ | ‡ | † |  |  |
|  | ethanol | ‡ | ‡ | † |  |  |
|  | ethyl acetate | ‡ | ‡ | † |  |  |
|  | hexane | ‡ | ‡ | 8  (20 µl/well)  (conc. 50 mg·ml^-1^) |  |  |
|  | methanol | ‡ | ‡ | † |  |  |
| *Lasallia pustulata* | acetone | † | ‡ | † | BTDM; DDM | [5] |
|  | methanol | † | ‡ | † | BTDM; DDM | [5] |
|  |  | 5 | ‡ | ‡ | MMwR | [6] |
|  | water | † | ‡ | † | BTDM; DDM | [5] |
| *Lathagrium cristatum* | acetone | † | ‡ | † | BTDM; DDM | [2] |
|  | methanol | † | ‡ | † |  |  |
|  | water | † | ‡ | † |  |  |
| *Lecanora frustulosa* | acetone | † | ‡ | † | BTDM; DDM | [7] |
|  |  | † | ‡ | † |  | [8] |
|  | methanol | 12.5 | ‡ | 15  (15 µl)  (conc. 50 mg·ml^-1^) | BTDM; DDM | [7] |
|  |  | 12.5 | ‡ | 15  (15 µl)  (conc. 50 mg·ml^-1^) |  | [8] |
|  | water | † | ‡ | † | BTDM; DDM | [7] |
|  |  | † | ‡ | † |  | [8] |
| *Ochrolechia parella* | methanol | † | ‡ | † | BTDM; DDM | [9] |
| *Ochrolechia tartarea* | methanol | 30 | ‡ | 7  (10 µl) (300 µg/disc)  (conc. 30 mg·ml^-1^) | BMM; DDM | [1] |
| *Protoparmeliopsis muralis* | acetone | † | ‡ | † | BTDM; DDM | [10] |
|  |  | † | ‡ | ‡ | BMM | [11] |
|  | methanol | 25 | ‡ | 14  (15 µl)  (conc. 50 mg·ml^-1^) | BTDM; DDM | [10] |
|  | water | † | ‡ | † | BTDM; DDM | [10] |
| *Tephromela atra* | acetone | 25 | ‡ | ‡ | BMM | [11] |
|  |  | 25 | ‡ | 12  (15 µl)  (conc. 50 mg·ml^-1^) | BTDM; DDM | [12] |
|  |  | 25 | ‡ | 12  (15 µl)  (conc. 50 mg·ml^-1^) | BTDM; DDM | [10] |
|  | methanol | 6.25 | ‡ | 18  (15 µl)  (conc. 50 mg·ml^-1^) | BTDM; DDM | [12] |
|  |  | 6.25 | ‡ | 18  (15 µl)  (conc. 50 mg·ml^-1^) | BTDM; DDM | [10] |
|  | water | † | ‡ | † | BTDM; DDM | [12] |
|  |  | † | ‡ | † | BTDM; DDM | [10] |
| *Umbilicaria crustulosa* | acetone | 25 | ‡ | ‡ | MMwR | [13] |
|  |  | 25 | ‡ | 8  (15 µl) | BTDM; DDM | [5] |
|  | methanol | 6.25 | ‡ | 11  (15 µl) | BTDM; DDM | [5] |
|  | water | † | ‡ | † | BTDM; DDM | [5] |
| *Umbilicaria cylindrica* | acetone | † | ‡ | ‡ | MMwR | [13] |
|  |  | † | ‡ | † | BTDM; DDM | [5] |
|  | methanol | † | ‡ | † | BTDM; DDM | [5] |
|  | water | † | ‡ | † | BTDM; DDM | [5] |
| *Umbilicaria nylanderiana* | methanol | 62.5 × 10^-3^ | ‡ | 33  (300 µg/disk)  (conc. 30 mg·ml^-1^) | BMM; DDM | [14] |
| *Umbilicaria polyphylla* | acetone | 12.5 | ‡ | ‡ | MMwR | [13] |
|  |  | 12.5 | ‡ | 12  (15 µl)  (conc. 50 mg·ml^-1^) | BTDM; DDM | [15] |
|  |  | 12.5 | ‡ | ‡ | BMM | [16] |
|  | methanol | 1.56 | ‡ | 22  (15 µl)  (conc. 50 mg·ml^-1^) | BTDM; DDM | [15] |
|  |  | 1.56 | ‡ | ‡ | BMM | [16] |
|  | water | † | ‡ | † | BTDM; DDM | [15] |
|  |  | † | ‡ | ‡ | BMM | [16] |

† no effect, ‡ not investigated

**Table S9.** Effect of crude extracts from corticolous lichens on *Fusarium roseum*. Method of measurement abbreviations: DDM = Disk diffusion method (IZ). For MIC, MFC and IZ abbreviations: see Tab. S3. Literature abbreviations: [1] Tiwari et al. (2011a); [2] Tiwari et al. (2011b).

| **Lichen species** | **Extracting solvent** | **Results** | | | **Measurement method** | **Literature** |
| --- | --- | --- | --- | --- | --- | --- |
|  |  | **MIC**  **[**mg·ml^-1^] | **MFC**  **[**mg·ml^-1^] | **IZ** [mm]  (dose) |  |  |
| *Bulbothrix setschwanensis* | acetone | ‡ | ‡ | c. 14 (5 ml)  (conc. 50 mg·ml^-1^) | DDM | [1] |
|  | chloroform | ‡ | ‡ | † |  |  |
|  | methanol | ‡ | ‡ | c. 6 (5 ml)  (conc. 50 mg·ml^-1^) |  |  |
| *Hypotrachyna nepalensis* | acetone | ‡ | ‡ | † | DDM | [1] |
|  | chloroform | ‡ | ‡ | † |  |  |
|  | methanol | ‡ | ‡ | c. 11 (5 ml)  (conc. 50 mg·ml^-1^) |  |  |
| *Heterodermia diademata* | acetone | ‡ | ‡ | c. 14 (5 ml)  (conc. 50 mg·ml^-1^) | DDM | [1] |
|  | chloroform | ‡ | ‡ | † |  |  |
|  | methanol | ‡ | ‡ | c. 13 (5 ml)  (conc. 50 mg·ml^-1^) |  |  |
| *Parmotrema thomsonii* | acetone | ‡ | ‡ | c. 13 (5 ml)  (conc. 50 mg·ml^-1^) | DDM | [1] |
|  | chloroform | ‡ | ‡ | c. 15 (5 ml)  (conc. 50 mg·ml^-1^) |  |  |
|  | methanol | ‡ | ‡ | c. 25 (5 ml)  (conc. 50 mg·ml^-1^) |  |  |
| *Parmotrema tinctorum* | acetone | ‡ | ‡ | 14 (5 ml) | DDM | [2] |
|  | chloroform | ‡ | ‡ | † |  |  |
|  | methanol | ‡ | ‡ | 18 (5 ml) |  |  |

† no effect, ‡ not investigated

**Table S10**. Effect of crude extracts from terricolous lichen on *Fusarium udum*. Method of measurement abbreviations: PFT = Poisoned Food Technique (ED_50_); ED_50_ = Effective Dose required for 50% growth inhibition. For MIC, MFC and IZ abbreviations: see Tab. S3. Literature abbreviations: [1] Goel et al. (2011b).

| **Lichen species** | **Extracting solvent** | **Results** | | | | **Measurement method** | **Literature** |
| --- | --- | --- | --- | --- | --- | --- | --- |
|  |  | **MIC**  **[**mg·ml^-1^] | **MFC**  **[**mg·ml^-1^] | **IZ** [mm]  (dose) | **Other** |  |  |
| *Parmotrema reticulatum* | ethyl acetate | ‡ | ‡ | ‡ | ED_50_: 43.7 µg·ml^-1^ | PFT | [1] |
|  | hexane | ‡ | ‡ | ‡ | ED_50_: 200.3 µg·ml^-1^ |  |  |
|  | methanol | ‡ | ‡ | ‡ | ED_50_: 59.2 µg·ml^-1^ |  |  |

‡ not investigated

**Table S11.** Effect of lichen secondary metabolites on *Fusarium culmorum*. Method of measurement abbreviations: DDM = Disk Diffusion Method (MIC). For MIC abbreviation: see Tab. S3. Literature abbreviations: [1] Türk et al. (2006).

| **Biochemical class** | **Secondary metabolites** | **Extracting solvent** | **Results** | **Measurement method** | **Literature** |
| --- | --- | --- | --- | --- | --- |
|  |  |  | **MIC [**mg·ml^-1^] |  |  |
| orcinol depsides | olivetoric acid | acetone | 10  [= 500 µg·50 µl^-1^] | DDM | [1] |
| β-orcinol depsides | chloroatranorin |  | 7.5  [= 300 µg·40 µl^-1^] |  |  |

**Table S12.** Effect of lichen secondary metabolites on *Fusarium oxysporum*. Method of measurement abbreviations: BTDM = Broth tube dilution method (MIC); DDM = Disk Diffusion Method (MIC);
MMwR = Microdilution method with resazurin (MIC). For MIC and IZ abbreviation: see Tab. S3. Literature abbreviations: [1] Hickey et al. (1990); [2] Ranković et al. (2010c); [3] Kosanić et al. (2010); [4] Ranković
and Mišić (2008); [5] Ristić et al. (2016b); [6] Türk et al. (2006); [7] Ristić et al. (2016a); [8] Aravind
et al. (2014); [9] König and Wright (1999); [10] Ranković et al. (2008); [11] Kosanić and Ranković (2011a); [12] Kosanić and Ranković (2011b).

| **Biochemical class** | **Secondary metabolites** | **Extracting solvent** | **Results** | | **Measurement method** | **Literature** |
| --- | --- | --- | --- | --- | --- | --- |
|  |  |  | **MIC [**mg·ml^-1^] | **IZ** [mm] (dose) |  |  |
| monocyclic aromatic derivative | methyl haematommate | methanol | ‡ | 4  (340 µg/disk)  (disk applied before germination) | DDM | [1] |
|  |  |  | ‡ | †  (disk applied after germination) |  |  |
| orcinol depsides | divaricatic acid | acetone;  methanol;  water | 6.25 | ‡ | BTDM | [2] |
|  |  | acetone;  methanol;  water | 6.25 | ‡ | BTDM | [3] |
|  | lecanoric acid | acetone;  methanol | 0.125 | ‡ | BTDM | [4] |
|  |  | acetone | 1 | ‡ | MMwR | [5] |
|  | olivetoric acid | acetone | 10  [= 500 µg·50 µl^-1^] | ‡ | DDM | [6] |
|  | methyl evernate | acetone | 0.5 | ‡ | MMwR | [7] |
|  | 2'-O-methylanziaic acid | acetone | 0.25 | ‡ | MMwR | [5] |
| orcinol β-orcinol depsides | obtusatic acid | acetone | 1 | ‡ | MMwR | [7] |
| β-orcinol depsides | atranorin | chloroform | 16 × 10^-3^ | 19  (conc. 16 × 10^-3^ mg·ml^-1^) | BMM; DDM | [8] |
|  |  | dichloromethane (methylene chloride) | ‡ | † | DDM | [9] |
|  |  | acetone;  methanol | 0.5 | ‡ | BTDM | [10] |
|  | chloroatranorin | acetone | 7.5  [= 300 µg·40 µl^-1^] | ‡ | DDM | [6] |
|  | 2-hydroxy-4-methoxy-3,6-dimethylbenzoic acid | chloroform | 4 × 10^-3^ | 26  (conc. 4 × 10^-3^ mg·ml^-1^) | BMM; DDM | [8] |
| orcinol depsidone | physodic acid | acetone;  methanol | 1 | ‡ | BTDM | [10] |
|  |  | acetone;  methanol;  water | 0.25 | ‡ | BMM | [11] |
| β-orcinol depsidones | fumarprotocetraric acid | acetone;  methanol | 0.25 | ‡ | BTDM | [4] |
|  |  | acetone;  methanol;  water | 0.25 | ‡ | BMM | [11] |
|  | protocetraric acid | acetone;  methanol | 0.5 | ‡ | BTDM | [4] |
|  | salazinic acid | acetone;  methanol;  water | 3.12 | ‡ | BTDM | [12] |
|  | stictic acid | acetone;  methanol | 1 | ‡ | BTDM | [4] |
| orcinol tridepside | gyrophoric acid | acetone;  methanol | 0.25 | ‡ | BTDM | [10] |
|  |  | acetone;  methanol;  water | 0.25 | ‡ | BMM | [11] |
| pulvinic acid derivative | vulpinic acid | dichloromethane (methylene chloride) | ‡ | † | DDM | [9] |
| terpenoid | hopane-6α, 22-diol (zeorin) | dichloromethane (methylene chloride) | ‡ | † | DDM | [9] |
|  |  | acetone;  methanol;  water | 3.12 | ‡ | BTDM | [2] |
|  |  | acetone;  methanol;  water | 3.12 | ‡ | BTDM | [3] |
| usnic acid derivative | usnic acid | dichloromethane (methylene chloride) | ‡ | † | DDM | [9] |
|  |  | acetone;  methanol | 0.5 | ‡ | BTDM | [10] |
|  | (+)-usnic acid | chloroform | 16 × 10^-3^ | 13  (conc. 16 × 10^-3^ mg·ml^-1^) | BMM; DDM | [8] |

† no effect, ‡ not investigated

**Table S13.** Effect of lichen secondary metabolites on *Fusarium solani*. Method of measurement abbreviations: DDM = Disk Diffusion Method (MIC); PFT = Poisoned Food Technique (IG%); MMwR = Microdilution method with resazurin (MIC); IG% = percentage growth inhibition of mycelium compared to the diameter
of mycelium in control sample. For MIC abbreviation: see Tab. S3. Literature abbreviations: [1] Sarıözlü
et al. (2016); [2] Thadhani et al. (2012); [3] Türk et al. (2006); [4] Cankılıç et al. (2017).

| **Biochemical class** | **Secondary metabolites** | **Extracting solvent** | **Results** | | **Measurement method** | **Literature** |
| --- | --- | --- | --- | --- | --- | --- |
|  |  |  | **MIC [**mg·ml^-1^] | **Other** |  |  |
| benzyl esters | barbatolic acid | methanol | 200 × 10^-3^ | ‡ | MMwR | [1] |
| monocyclic aromatic derivatives | methyl orsellinate | dichloromethane  (methylene chloride);  methanol | ‡ | IG%: 70  (conc. 200 µg·ml^-1^) | PFT | [2] |
|  | methyl β-orcinol carboxylate | dichloromethane  (methylene chloride);  methanol | ‡ | IG%: 90  (conc. 200 µg·ml^-1^) |  |  |
| orcinol depsides | erythrin | dichloromethane  (methylene chloride);  methanol | ‡ | † | PFT | [2] |
|  | lecanoric acid | dichloromethane  (methylene chloride);  methanol | ‡ | IG%: 50  (conc. 200 µg·ml^-1^) | PFT | [2] |
|  | olivetoric acid | acetone | 10  [= 500 µg·50 µl^-1^] | ‡ | DDM | [3] |
|  | sekikaic acid | dichloromethane  (methylene chloride);  methanol | ‡ | † | PFT | [2] |
| β-orcinol depsides | atranorin | dichloromethane  (methylene chloride);  methanol | ‡ | † | PFT | [2] |
|  | chloroatranorin | acetone | 7.5  [= 300 µg·40 µl^-1^] | ‡ | DDM | [3] |
|  | thamnolic acid | methanol | † | ‡ | MMwR | [4] |
| orcinol depsidones | lobaric acid | dichloromethane  (methylene chloride);  methanol | ‡ | † | PFT | [2] |

† no effect, ‡ not investigated

**Table S14.** Effect of lichen secondary metabolites on *Fusarium udum*. Method of measurement abbreviations: PFT = Poisoned Food Technique (IG%, ED_50_); IG% = percentage growth inhibition of mycelium compared
to the diameter of mycelium in control sample; ED_50_ = Effective Dose required for 50% growth inhibition.
For MIC abbreviation: see Tab. S3. Literature abbreviations: [1] Goel et al. (2011b); [2] Goel and Singh (2015).

| **Biochemical class** | **Secondary metabolites** | **Extracting solvent** | **Results** | | **Measurement method** | **Literature** |
| --- | --- | --- | --- | --- | --- | --- |
|  |  |  | **MIC [**mg·ml^-1^] | **Other** |  |  |
| aliphatic acids | protolichesterinic acid | ethyl acetate;  hexane;  methanol | ‡ | IG%: 90.33  (conc. 250 µg·ml^-1^)  ED_50_: 55.68 µg·ml^-1^ | PFT | [1] |
| monocyclic aromatic derivatives | ethyl hematommate | ethyl acetate;  hexane;  methanol | ‡ | IG%: 27.40  (conc. 250 µg·ml^-1^)  ED_50_: 250.36 µg·ml^-1^ | PFT | [1] |
|  | ethyl orsellinate | ethyl acetate;  hexane;  methanol | ‡ | IG%: 79.43  (conc. 250 µg·ml^-1^)  ED_50_: 98.20 µg·ml^-1^ |  |  |
|  | evernyl  (methyl β-orcinol carboxylate) | ethyl acetate;  hexane;  methanol | ‡ | IG%: 80.46  (conc. 250 µg·ml^-1^)  ED_50_: 82.31 µg·ml^-1^ |  |  |
|  | methyl haematommate | ethyl acetate;  hexane;  methanol | ‡ | IG%: 67.33  (conc. 250 µg·ml^-1^)  ED_50_: 71.22 µg·ml^-1^ |  |  |
| orcinol depsides | homosekikaic acid | ethyl acetate | ‡ | ED_50_: 147.18 µg·ml^-1^ | PFT | [2] |
|  | sekikaic acid | ethyl acetate;  hexane;  methanol | ‡ | ED_50_: 32.85 µg·ml^-1^ |  |  |
| β-orcinol depsides | atranorin | ethyl acetate;  hexane;  methanol | ‡ | IG%: 90  (conc. 250 µg·ml^-1^)  ED_50_: 50.32 µg·ml^-1^ | PFT | [1] |
|  | baeomycesic acid | ethyl acetate;  hexane;  methanol | ‡ | IG%: 57.86  (conc. 250 µg·ml^-1^)  ED_50_: 109.85 µg·ml^-1^ |  |  |
|  | 2-hydroxy-4-methoxy-3,6-dimethylbenzoic acid | ethyl acetate;  hexane;  methanol | ‡ | IG%: 58.00  (conc. 250 µg·ml^-1^)  ED_50_: 106.13 µg·ml^-1^ |  |  |
| β-orcinol depsidones | salazinic acid | ethyl acetate;  hexane;  methanol | ‡ | IG%: 67.00  (conc. 250 µg·ml^-1^)  ED_50_: 88.20 µg·ml^-1^ | PFT | [1] |
| usnic acid derivative | isousnic acid | ethyl acetate;  hexane;  methanol | ‡ | IG%: 81  (conc. 250 µg·ml^-1^)  ED_50_: 70.86 µg·ml^-1^ | PFT | [1] |
| xanthone | lichexanthone | ethyl acetate;  hexane;  methanol | ‡ | IG%: 53.33  (conc. 250 µg·ml^-1^)  ED_50_: 151.03 µg·ml^-1^ | PFT | [1] |

‡ not investigated

Table S15A-B. Activity of antifungal non-lichen substances against *Fusarium* fungi follow: [1] Anjali et al. (2015a); [2] Anjali et al. (2015b); [3] Aslan et al. (2006);
[4] Gulluce et al. (2006); [5] Ranković and Mišić (2007); [6] Ranković et al. (2010a); [7] Ranković et al. (2007a); [8] Tiwari et al. (2011a); [9] Grujičić et al. (2014);
[10] Ranković et al. (2010b); [11] Ranković et al. (2009); [12] Ranković et al. (2011); [13] Kosanić and Ranković (2011a); [14] Balasubramanian and Nirmala (2014);
[15] Shivanna and Garampalli (2014); [16] Babiah et al. (2014a); [17] Kosanić et al. (2013); [18] Ranković et al. (2007b); [19] Kosanić et al. (2016); [20] Kosanić
and Ranković (2011b); [21] Ranković and Kosanić (2012); [22] Ranković et al. (2010c); [23] Kosanić et al. (2010); [24] Kosanić et al. (2012a); [25] Babiah et al. (2014b); [26] Tiwari et al. (2011b); [27] Ranković et al. (2014); [28] Kosanić et al. (2012b); [29] Shivanna and Garampalli (2015); [30] Babiah et al. (2014b); [31] Goel et al. (2011b); [32] Devashree et al. (2019); [33] Kosanić et al. (2018); [34] Ristić et al. (2016a); [35] Thippeswamy et al. (2013); [36] Yadav et al. (2021); [37] Hanuš et al. (2007);
[38] Ranković et al. (2008); [39] Ranković and Mišić (2008); [40] Thadhani et al. (2012); [41] Ristić et al. (2016b); [42] Aravind et al. (2014); [43] Sarıözlü et al. (2016);
[44] Borman et al. (2017); [45] Pujol et al. (1997); [46] Herkert et al. (2019); [47] Al-Hatmi et al. (2015); [48] Alastruey-Izquierdo et al. (2008); [49] Guarro et al. (1999); [50] Al-Hatmi et al. (2018); [51] Wolny-Koładka (2016); [52] Köycü (2018).

* = not given; † = no effect; ‡ = MFC_90_; 48 h/72 h = mean value of MICs after 48 and 72 h of experiment, respectively; IR% = percentage fungus inhibition rate;
ED_50_ = Effective Dose required for 50% growth inhibition. For MIC, MFC and IZ abbreviations: see Tab. S3. All values are given in mg·ml^-1^ for MIC/MFC or in mm for IZ. In some cases, units have been converted to standardize the results. *Fusarium avenaceum* species was not included in the table because of the impossibility of comparing the results obtained by different measurement methods (see: Tab. S4).

| Activity of antifungal non-lichen substances (part A) | *Fusarium acuminatum* | *Fusarium culmorum* | *Fusarium fujikuroi* | | *Fusarium oxysporum* | |
| --- | --- | --- | --- | --- | --- | --- |
|  | MIC | MIC | MIC | IZ | MIC | IZ |
| Amphotericin B | 62.5 × 10^-3^ [3] [4] | 0.023 × 10^-3^ [51] | 1.98 × 10^-3^ (48 h) [45]  3.8 × 10^-3^ (72 h) [45]  1 × 10^-3^ [46]  2 × 10^-3^ [46]  0.5-1 × 10^-3^ [47]  1-2 × 10^-3^ [47]  0.25-1 × 10^-3^ [47]  0.5-32 × 10^-3^ [48]  0.25-1 × 10^-3^ [50]  0.5-1 × 10^-3^ [50]  1-2 × 10^-3^ [50] | * | 62.5 × 10^-3^ [3] [4]  2.13 × 10^-3^ (48 h) [45]  5.65 × 10^-3^ (72 h) [45]  0.12-2 × 10^-3^ [48]  0.5-2 × 10^-3^ [50] | * |
| Bavistin  (systemic fungicide) | * | * | * | * | 16 × 10^-3^ [42] | 16.5 [15]  11 [35]  18  (conc. 62.5 × 10^-3^ mg·ml^-1^) [42] |
| Caspofungin | * | 0.25 × 10^-3^ [51] | * | * | * | * |
| Clotrimazole | * | * | 18.8 × 10^-3^ [37] | * | * | * |
| Difenoconazole  (fungicide) | * | * | 8 × 10^-3^ [46]  8 × 10^-3^ [46] | * | * | * |
| Flucytosine | * | * | 645.4 × 10^-3^ (48 h) [45]  645.4 × 10^-3^ (72 h) [45] | * | 645.4 × 10^-3^ (48 h) [45]  645.4 × 10^-3^ (72 h) [45] | * |
| Fluconazole | * | † [51] | 160 × 10^-3^ (48 h) [45]  160 × 10^-3^ (72 h) [45]  64->64 × 10^-3^ [47]  64->64 × 10^-3^ [47]  >64-1 × 10^-3^ [47] | * | 160 × 10^-3^ (48 h) [45]  160 × 10^-3^ (72 h) [45] | * |
| Isavuconazole | * | * | 4 × 10^-3^ [46]  4 × 10^-3^ [46]  2-4 × 10^-3^ [47]  1-2 × 10^-3^ [47]  4-16 × 10^-3^ [47] | * | * | * |
| Itraconazole | * | † [51] | 20 × 10^-3^ (48 h) [45]  20 × 10^-3^ (72 h) [45]  64 × 10^-3^ [46]  64 × 10^-3^ [46]  >16 × 10^-3^ [47]  16->16 × 10^-3^ [47]  16->16 × 10^-3^ [47]  1-16 × 10^-3^ [48] | * | 19.01 × 10^-3^ (48 h) [45]  19.21 × 10^-3^ (72 h) [45]  1-16 × 10^-3^ [48] | * |
| Ketoconazole | * | † [51] | 39.06 × 10^-3^ [43]  38.86 × 10^-3^ (48 h) [45]  46.17 × 10^-3^ (72 h) [45] | 11.33 [1]  11 [2]  38 [43] | 3.75 × 10^-3^ [6] [10]  3.9 × 10^-3^ [7] [9] [11] [12] [13] [17] [18] [19] [20] [21] [22] [23] [24] [27] [28] [38] [39]  78 × 10^-3^ [33] [34] [41]  33.26 × 10^-3^ (48 h) [45]  43.54 × 10^-3^ (72 h) [45] | 30 (30 µg·ml^-1^) [6]  35 (1 mg·ml^-1^) [7]  † [8]  35 (30 µg·ml^-1^) [10]  35 [11] [18] [20] [21] [22] [23]  10 (1 mg·ml^-1^) [14]  17.3 [16]  17.3 [25]  20 [26]  15.33 [2]  14 [32]  † [36]  12 [36]  14 [36] |
| Micafungin | * | * | >8 × 10^-3^ [47]  >8 × 10^-3^ [47]  >8 × 10^-3^ [47] | * | * | * |
| Miconazole | * | * | 69.29 × 10^-3^ (48 h) [45]  70 × 10^-3^ (72 h) [45] | * | 71.68 × 10^-3^ (48 h) [45]  71.74 × 10^-3^ (72 h) [45] | * |
| Natamycin | * | * | 4 × 10^-3^ [47]  4 × 10^-3^ [47]  4 × 10^-3^ [47]  4 × 10^-3^ [50]  4 × 10^-3^ [50]  4 × 10^-3^ [50] | * | 4 × 10^-3^ [50] | * |
| Posaconazole | * | 0.006 × 10^-3^ [51] | 0.5 × 10^-3^ [46]  1 × 10^-3^ [46]  0.125-0.25 × 10^-3^ [47]  0.25-1 × 10^-3^ [47]  1-2 × 10^-3^ [47]  0.25-16 × 10^-3^ [48]  1-2 × 10^-3^ [50]  0.125-0.25 × 10^-3^ [50]  0.25-1 × 10^-3^ [50] | * | 0.06-16 × 10^-3^ [48]  0.5-16 × 10^-3^ [50] | * |
| Propiconazole | * | * | 2 × 10^-3^ [46]  8 × 10^-3^ [46] | * | * | * |
| Ravuconazole | * | * | 2-16 × 10^-3^ [48] | * | 1-16 × 10^-3^ [48] | * |
| Terbinafine | * | * | 1-32 × 10^-3^ [48]  0.25-0.5 × 10^-3^ [50] | * | 15 × 10^-3^ [5]  0.5-32 × 10^-3^ [48]  2-8 × 10^-3^ [50] | * |
| Tebuconazole | * | 3 × 10^-3^ [52]  3 × 10^-3^ [52]  3 × 10^-3^ [52]  10 × 10^-3^ [52] | 4 × 10^-3^ [46]  4 × 10^-3^ [46] | * | * | * |
| Voriconazole | * | 0.016 × 10^-3^ [51] | 2 × 10^-3^ [46]  4 × 10^-3^ [46]  1 × 10^-3^ [47]  1-4 × 10^-3^ [47]  2-8 × 10^-3^ [47]  1-16 × 10^-3^ [48]  1-8 × 10^-3^ [50]  1-2 × 10^-3^ [50]  1-4 × 10^-3^ [50] | * | 0.5-16 × 10^-3^ [48]  2-8 × 10^-3^ [50] | * |

| Activity of antifungal non-lichen substances (part B) | *Fusarium roseum* | *Fusarium solani* | | | *Fusarium udum* |
| --- | --- | --- | --- | --- | --- |
|  | IZ | MIC | MFC | IZ | Other |
| Amphotericin B | * | 62.5 × 10^-3^ [3] [4]  3.47 × 10^-3^ (48 h) [45]  17.52 × 10^-3^ (72 h) [45]  2 × 10^-3^ [46]  0.5 × 10^-3^ [46]  0.5-8 × 10^-3^ [48]  2.31 × 10^-3^ [49]  1.16 × 10^-3^ [49]  1.16 × 10^-3^ [49]  1.16 × 10^-3^ [49]  1.16 × 10^-3^ [49]  1-2 × 10^-3^ [50] | 4^‡^ [44] | * | * |
| Bavistin  (systemic fungicide) | * | 1.562 [29] | * | 16 [29] | * |
| Caspofungin | * | * | >64^‡^ [44] | * | * |
| Difenoconazole | * | 64 × 10^-3^ [46]  64 × 10^-3^ [46] | * | * | * |
| Flucytosine | * | 645.4 × 10^-3^ (48 h) [45]  645.4 × 10^-3^ (72 h) [45]  322.75 × 10^-3^ [49]  322.75 × 10^-3^ [49]  322.75 × 10^-3^ [49]  322.75 × 10^-3^ [49]  322.75 × 10^-3^ [49] | * | * | * |
| Fluconazole | * | 160 × 10^-3^ (48 h) [45]  160 × 10^-3^ (72 h) [45] | * | * | * |
| Hexaconazole | * | * | * | * | IR%: 70 (conc. 31 µg·ml^-1^ ) [31]  ED_50_: 22.01 µg·ml^-1^ [31] |
| Isavuconazole | * | 64 × 10^-3^ [46]  64 × 10^-3^ [46] | * | * | * |
| Itraconazole | * | 20 × 10^-3^ (48 h) [45]  20 × 10^-3^ (72 h) [45]  64 × 10^-3^ [46]  64 × 10^-3^ [46]  16 × 10^-3^ [48] | >16^‡^ [44] | * | * |
| Ketoconazole | † [26] | 39.06 × 10^-3^ [43]  88.4 × 10^-3^ (48 h) [45]  92 × 10^-3^ (72 h) [45] | * | 15.3 [30]  † [26]  35 [43] | * |
| Miconazole | * | 74 × 10^-3^ [40]  77.5 × 10^-3^ (48 h) [45]  80 × 10^-3^ (72 h) [45] | * | * | * |
| Natamycin | * | 4 × 10^-3^ [50] | * | * | * |
| Posaconazole | * | 64 × 10^-3^ [46]  64 × 10^-3^ [46]  16 × 10^-3^ [48]  16 × 10^-3^ [50] | >16^‡^ [44] | * | * |
| Propiconazole | * | 64 × 10^-3^ [46]  64 × 10^-3^ [46] | * | * | * |
| Ravuconazole | * | 16 × 10^-3^ [48] | * | * | * |
| Rifampin | * | >40 × 10^-3^ [49]  >40 × 10^-3^ [49]  >40 × 10^-3^ [49]  >40 × 10^-3^ [49]  >40 × 10^-3^ [49] | * | * | * |
| Terbinafine | * | 16-32 × 10^-3^ [48] | * | * | * |
| Tebuconazole | * | 64 × 10^-3^ [46]  64 × 10^-3^ [46] | * | * | * |
| Voriconazole | * | 8 × 10^-3^ [46]  16 × 10^-3^ [46]  4-16 × 10^-3^ [48]  16 × 10^-3^ [50] | >16^‡^ [44] | * | * |

**Table S16.** Comparative statistical analysis between two sets of Minimal Inhibitory Concentration (MIC) values for extracts from corticolous, terricolous and saxicolous lichen species against *Fusarium oxysporum* based on literature data. Normality of data distribution was checked by Shapiro-Wilk test and λ Kolmogorov-Smirnov test with
a Lilliefors's amendment (N<30), and *X*^2^ test and λ Kolmogorov-Smirnov test (N≥30). Because the assumption of normality of data distribution was not met, homogeneity
of variance tests were not performed. Statistical significance was tested by U Mann-Whitney and λ Kolmogorov-Smirnov tests. Significance level
of α=0.05 was assumed for all tests. The compared variant with statistically significantly lower (methodologically beneficial) difference in mean MIC value is marked in red. Abbreviations: $\bar{x}$ = MIC arithmetic mean; SD = MIC Standard Deviation; Me = median; [S-W] = Shapiro-Wilk test; [*X*^2^] = *X*^2^ test; [λ] = Kolmogorov-Smirnov test;
[λ-L] = Kolmogorov-Smirnov with a Lilliefors’s amendment test; [U] = Mann-Whitney test. For literature references: see adequate Tab. S6-S8.

| **Group no.** | **Methodological combinations compared** | | | | **Variants general data** | | **Tests of distribution normality** | | **Statistical tests** |
| --- | --- | --- | --- | --- | --- | --- | --- | --- | --- |
| **MICs of crude extracts from corticolous (variant 1) lichen species *vs* MICs of crude extracts from terricolous (variant 2) lichen species on *Fusarium oxysporum*** | | | | | | | | | |
|  | **Variant 1** | | **Variant 2** | | **Variant 1** | **Variant 2** | **Variant 1** | **Variant 2** | **[U] p=0.007968**  **[λ] p<0.05** |
|  | **Lichen species** | **MIC [mg·ml^-1^]** | **Lichen species** | **MIC [mg·ml^-1^]** | **N = 65**  $\bar{\boldsymbol{x}}$ **= 12.47**  **SD = 17.47**  **Me = 6.25** | N = 14  $\bar{x}$ = 31.96  SD = 41.67  Me = 25 | [λ] p<0.01  [*X*^2^] p=0.00000 | [λ-L] p<0.01  [S-W] p=0.0001 |  |
| No. 1 | *Alectoria sarmentosa* | 50 | *Cetraria islandica* | 2.5 |  |  |  |  |  |
|  |  | 100 | *Cladonia fimbriata* | 5 |  |  |  |  |  |
|  | *Flavoparmelia caperata* | 1.562 | *Cladonia foliacea* | 10 |  |  |  |  |  |
|  |  | 6.25 | *Cladonia furcata* | 25 |  |  |  |  |  |
|  |  | 1.56 |  | 25 |  |  |  |  |  |
|  |  | 25 |  | 25 |  |  |  |  |  |
|  |  | 6.25 |  | 10 |  |  |  |  |  |
|  |  | 1.875 |  | 25 |  |  |  |  |  |
|  |  | 0.097 |  | 25 |  |  |  |  |  |
|  |  | 6.25 | *Cladonia rangiferina* | 10 |  |  |  |  |  |
|  |  | 1.56 |  | 100 |  |  |  |  |  |
|  | *Hypogymnia physodes* | 12.5 |  | 150 |  |  |  |  |  |
|  |  | 12.5 | *Gyalolechia fulgens* | 30 |  |  |  |  |  |
|  |  | 12.5 | *Stereocaulon paschale* | 5 |  |  |  |  |  |
|  |  | 6.25 |  |  |  |  |  |  |  |
|  |  | 6.25 |  |  |  |  |  |  |  |
|  | *Melanelixia fuliginosa* | 2.5 |  |  |  |  |  |  |  |
|  | *Melanelixia subaurifera* | 10 |  |  |  |  |  |  |  |
|  | *Menegazzia terebrata* | 1.56 |  |  |  |  |  |  |  |
|  |  | 6.25 |  |  |  |  |  |  |  |
|  |  | 1.56 |  |  |  |  |  |  |  |
|  | *Ochrolechia androgyna* | 3.12 |  |  |  |  |  |  |  |
|  |  | 50 |  |  |  |  |  |  |  |
|  | *Parmelia omphalodes* | 3.12 |  |  |  |  |  |  |  |
|  |  | 3.12 |  |  |  |  |  |  |  |
|  | *Parmelia saxatilis* | 25 |  |  |  |  |  |  |  |
|  |  | 25 |  |  |  |  |  |  |  |
|  |  | 12.5 |  |  |  |  |  |  |  |
|  | *Parmelia sulcata* | 1.56 |  |  |  |  |  |  |  |
|  |  | 25 |  |  |  |  |  |  |  |
|  |  | 6.25 |  |  |  |  |  |  |  |
|  |  | 25 |  |  |  |  |  |  |  |
|  |  | 3.12 |  |  |  |  |  |  |  |
|  | *Parmeliopsis ambigua* | 25 |  |  |  |  |  |  |  |
|  |  | 25 |  |  |  |  |  |  |  |
|  |  | 6.25 |  |  |  |  |  |  |  |
|  | *Parmotrema austrosinense* | 1.562 |  |  |  |  |  |  |  |
|  |  | 3.125 |  |  |  |  |  |  |  |
|  |  | 0.390 |  |  |  |  |  |  |  |
|  | *Parmotrema grayanum* | 3.125 |  |  |  |  |  |  |  |
|  | *Parmeliopsis hyperopta* | 12.5 |  |  |  |  |  |  |  |
|  |  | 12.5 |  |  |  |  |  |  |  |
|  |  | 3.12 |  |  |  |  |  |  |  |
|  |  | 3.12 |  |  |  |  |  |  |  |
|  | *Parmotrema crinitum* | 15 |  |  |  |  |  |  |  |
|  | *Parmotrema reticulatum* | 12.5 |  |  |  |  |  |  |  |
|  |  | 50 |  |  |  |  |  |  |  |
|  |  | 50 |  |  |  |  |  |  |  |
|  |  | 6.25 |  |  |  |  |  |  |  |
|  |  | 50 |  |  |  |  |  |  |  |
|  | *Parmotrema tinctorum* | 3.125 |  |  |  |  |  |  |  |
|  |  | 6.25 |  |  |  |  |  |  |  |
|  | *Physcia aipolia* | 6.25 |  |  |  |  |  |  |  |
|  |  | 0.39 |  |  |  |  |  |  |  |
|  |  | 25 |  |  |  |  |  |  |  |
|  |  | 0.39 |  |  |  |  |  |  |  |
|  | *Physcia ceasia* | 6.25 |  |  |  |  |  |  |  |
|  |  | 3.12 |  |  |  |  |  |  |  |
|  | *Ramalina fastigiata* | 2.5 |  |  |  |  |  |  |  |
|  | *Ramalina fraxinea* | 5 |  |  |  |  |  |  |  |
|  | *Roccella montagnei* | 3.125 |  |  |  |  |  |  |  |
|  |  | 0.39 |  |  |  |  |  |  |  |
|  |  | 0.39 |  |  |  |  |  |  |  |
|  | *Teloschistes flavicans* | 6.25 |  |  |  |  |  |  |  |
|  | *Vulpicida pinastri* | 7.5 |  |  |  |  |  |  |  |
| **MICs of crude extracts from corticolous (variant 1) lichen species *vs* MICs of crude extracts from saxicolous (variant 2) lichen species on *Fusarium oxysporum*** | | | | | | | | | |
| No. 2 | *Alectoria sarmentosa* | 50 | *Arctoparmelia centrifuga* | 30 | **N = 65**  $\bar{\boldsymbol{x}}$ **= 12.47**  **SD = 17.47**  **Me = 6.25** | N = 22  $\bar{x}$ = 15.88  SD = 10.18  Me = 12.5 | [λ] p<0.01  [*X*^2^] p=0.00000 | [λ-L] p<0.01  [S-W] p=0.0072 | **[U] p=0.028104**  **[λ] p<0.05** |
|  |  | 100 | *Aspicilia cinerea* | 25 |  |  |  |  |  |
|  | *Flavoparmelia caperata* | 1.562 |  | 25 |  |  |  |  |  |
|  |  | 6.25 | *Lasallia pustulata* | 5 |  |  |  |  |  |
|  |  | 1.56 | *Lecanora frustulosa* | 12.5 |  |  |  |  |  |
|  |  | 25 |  | 12.5 |  |  |  |  |  |
|  |  | 6.25 | *Ochrolechia tarterea* | 30 |  |  |  |  |  |
|  |  | 1.875 | *Protoparmeliopsis muralis* | 25 |  |  |  |  |  |
|  |  | 0.097 | *Tephromela atra* | 25 |  |  |  |  |  |
|  |  | 6.25 |  | 25 |  |  |  |  |  |
|  |  | 1.56 |  | 25 |  |  |  |  |  |
|  | *Hypogymnia physodes* | 12.5 |  | 6.25 |  |  |  |  |  |
|  |  | 12.5 |  | 6.25 |  |  |  |  |  |
|  |  | 12.5 | *Umbilicaria crustulosa* | 25 |  |  |  |  |  |
|  |  | 6.25 |  | 25 |  |  |  |  |  |
|  |  | 6.25 |  | 6.25 |  |  |  |  |  |
|  | *Melanelixia fuliginosa* | 2.5 | *Umbilicaria nylanderiana* | 62.5 × 10^-3^ |  |  |  |  |  |
|  | *Melanelixia subaurifera* | 10 | *Umbilicaria polyphylla* | 12.5 |  |  |  |  |  |
|  | *Menegazzia terebrata* | 1.56 |  | 12.5 |  |  |  |  |  |
|  |  | 6.25 |  | 12.5 |  |  |  |  |  |
|  |  | 1.56 |  | 1.56 |  |  |  |  |  |
|  | *Ochrolechia androgyna* | 3.12 |  | 1.56 |  |  |  |  |  |
|  |  | 50 |  |  |  |  |  |  |  |
|  | *Parmelia omphalodes* | 3.12 |  |  |  |  |  |  |  |
|  |  | 3.12 |  |  |  |  |  |  |  |
|  | *Parmelia saxatilis* | 25 |  |  |  |  |  |  |  |
|  |  | 25 |  |  |  |  |  |  |  |
|  |  | 12.5 |  |  |  |  |  |  |  |
|  | *Parmelia sulcata* | 1.56 |  |  |  |  |  |  |  |
|  |  | 25 |  |  |  |  |  |  |  |
|  |  | 6.25 |  |  |  |  |  |  |  |
|  |  | 25 |  |  |  |  |  |  |  |
|  |  | 3.12 |  |  |  |  |  |  |  |
|  | *Parmeliopsis ambigua* | 25 |  |  |  |  |  |  |  |
|  |  | 25 |  |  |  |  |  |  |  |
|  |  | 6.25 |  |  |  |  |  |  |  |
|  | *Parmotrema austrosinense* | 1.562 |  |  |  |  |  |  |  |
|  |  | 3.125 |  |  |  |  |  |  |  |
|  |  | 0.390 |  |  |  |  |  |  |  |
|  | *Parmotrema grayanum* | 3.125 |  |  |  |  |  |  |  |
|  | *Parmeliopsis hyperopta* | 12.5 |  |  |  |  |  |  |  |
|  |  | 12.5 |  |  |  |  |  |  |  |
|  |  | 3.12 |  |  |  |  |  |  |  |
|  |  | 3.12 |  |  |  |  |  |  |  |
|  | *Parmotrema crinitum* | 15 |  |  |  |  |  |  |  |
|  | *Parmotrema reticulatum* | 12.5 |  |  |  |  |  |  |  |
|  |  | 50 |  |  |  |  |  |  |  |
|  |  | 50 |  |  |  |  |  |  |  |
|  |  | 6.25 |  |  |  |  |  |  |  |
|  |  | 50 |  |  |  |  |  |  |  |
|  | *Parmotrema tinctorum* | 3.125 |  |  |  |  |  |  |  |
|  |  | 6.25 |  |  |  |  |  |  |  |
|  | *Physcia aipolia* | 6.25 |  |  |  |  |  |  |  |
|  |  | 0.39 |  |  |  |  |  |  |  |
|  |  | 25 |  |  |  |  |  |  |  |
|  |  | 0.39 |  |  |  |  |  |  |  |
|  | *Physcia ceasia* | 6.25 |  |  |  |  |  |  |  |
|  |  | 3.12 |  |  |  |  |  |  |  |
|  | *Ramalina fastigiata* | 2.5 |  |  |  |  |  |  |  |
|  | *Ramalina fraxinea* | 5 |  |  |  |  |  |  |  |
|  | *Roccella montagnei* | 3.125 |  |  |  |  |  |  |  |
|  |  | 0.39 |  |  |  |  |  |  |  |
|  |  | 0.39 |  |  |  |  |  |  |  |
|  | *Teloschistes flavicans* | 6.25 |  |  |  |  |  |  |  |
|  | *Vulpicida pinastri* | 7.5 |  |  |  |  |  |  |  |
| **MICs of crude extracts from terricolous (variant 1) lichens species *vs* MICs of crude extracts from saxicolous (variant 2) lichen species on *Fusarium oxysporum*** | | | | | | | | | |
| No. 3 | *Cetraria islandica* | 2.5 | *Arctoparmelia centrifuga* | 30 | N= 14  $\bar{x}$ = 31.96  SD = 41.67  Me = 25 | N = 22  $\bar{x}$ = 15.88  SD = 10.18  Me = 12.5 | [λ-L] p<0.01  [S-W] p=0.0001 | [λ-L] p<0.01  [S-W] p=0.0072 | [U] p=0.473741  [λ] p<0.1 |
|  | *Cladonia fimbriata* | 5 | *Aspicilia cinerea* | 25 |  |  |  |  |  |
|  | *Cladonia foliacea* | 10 |  | 25 |  |  |  |  |  |
|  | *Cladonia furcata* | 25 | *Lasallia pustulata* | 5 |  |  |  |  |  |
|  |  | 25 | *Lecanora frustulosa* | 12.5 |  |  |  |  |  |
|  |  | 25 |  | 12.5 |  |  |  |  |  |
|  |  | 10 | *Ochrolechia tarterea* | 30 |  |  |  |  |  |
|  |  | 25 | *Protoparmeliopsis muralis* | 25 |  |  |  |  |  |
|  |  | 25 | *Tephromela atra* | 25 |  |  |  |  |  |
|  | *Cladonia rangiferina* | 10 |  | 25 |  |  |  |  |  |
|  |  | 100 |  | 25 |  |  |  |  |  |
|  |  | 150 |  | 6.25 |  |  |  |  |  |
|  | *Gyalolechia fulgens* | 30 |  | 6.25 |  |  |  |  |  |
|  | *Stereocaulon paschale* | 5 | *Umbilicaria crustulosa* | 25 |  |  |  |  |  |
|  |  |  |  | 25 |  |  |  |  |  |
|  |  |  |  | 6.25 |  |  |  |  |  |
|  |  |  | *Umbilicaria nylanderiana* | 62.5 × 10^-3^ |  |  |  |  |  |
|  |  |  | *Umbilicaria polyphylla* | 12.5 |  |  |  |  |  |
|  |  |  |  | 12.5 |  |  |  |  |  |
|  |  |  |  | 12.5 |  |  |  |  |  |
|  |  |  |  | 1.56 |  |  |  |  |  |
|  |  |  |  | 1.56 |  |  |  |  |  |

**Table S17.** Comparative statistical analysis between two sets of Inhibition Zone (IZ) values for extracts from corticolous, terricolous and saxicolous lichen species against *Fusarium oxysporum* based on literature data. Normality of data distribution was checked by Shapiro-Wilk and λ Kolmogorov-Smirnov with a Lilliefors's amendment tests (N<30), and *X*^2^ test and λ Kolmogorov-Smirnov tests (N≥30). Because the assumption of normality of data distribution was not met, homogeneity of variance tests were not performed. In the case of group no. 1, for which the assumption of data distribution normality was met on the basis of *X*^2^ (variant 1) and Shapiro-Wilk tests (variant 2), the analysis of homogeneity of variance was not performed due to (1) lack of meeting the assumption of data distribution normality by λ Kolmogorov-Smirnov (variant 1) and
λ Kolmogorov-Smirnov with a Lilliefors’s amendment (variant 2) tests and (2) due to low data quantity (N=5) of variant 2, which may determine the causality fulfillment of the normality assumption of data distribution for this variant. The analysis of statistical significance was checked with the U Mann-Whitney and the λ Kolmogorov-Smirnov tests. A significance level of α=0.05 was assumed for all tests. Abbreviations: $\bar{x}$ = IZ arithmetic mean; SD = IZ Standard Deviation; Me = median; [S-W] = Shapiro-Wilk test; [*X*^2^] = *X*^2^ test; [λ] = Kolmogorov-Smirnov test; [λ-L] = Kolmogorov-Smirnov with a Lilliefors’s amendment test; [U] = Mann-Whitney test. For literature references: see adequate Tab. S6-S8.

| **Group no.** | **Methodological combinations compared** | | | | **Variants general data** | | **Tests of distribution normality**  **(normal distribution in bold)** | | **Statistical tests** |
| --- | --- | --- | --- | --- | --- | --- | --- | --- | --- |
| **IZs of crude extracts from corticolous (variant 1) lichen species *vs* IZs of crude extracts from terricolous (variant 2) lichen species on *Fusarium oxysporum*** | | | | | | | | | |
|  | **Variant 1** | | **Variant 2** | | **Variant 1** | **Variant 2** | **Variant 1** | **Variant 2** |  |
|  | **Lichen species** | **IZ**  **[mm]** | **Lichen species** | **IZ**  **[mm]** | N = 93  $\bar{x}$ = 14.95  SD = 5.36  Me = 14 | N = 5  $\bar{x}$ = 12.4  SD = 1.14  Me = 12 | [λ] p=not significant  **[*X*^2^] p=0.07174** | [λ-L] p=not significant  **[S-W] p=0.8140** | [U] p=0.257594  [λ] p>0.1 |
| No. 1 | *Alectoria sarmentosa* | 18 | *Cladonia furcata* | 11 |  |  |  |  |  |
|  |  | 15 |  | 12 |  |  |  |  |  |
|  | *Bulbothrix setschwanensis* | 10 | *Cladonia rangiferina* | 14 |  |  |  |  |  |
|  |  | 12 |  | 13 |  |  |  |  |  |
|  |  | 6 | *Gyalolechia fulgens* | 12 |  |  |  |  |  |
|  | *Cetrelia braunsiana* | 22 |  |  |  |  |  |  |  |
|  |  | 25 |  |  |  |  |  |  |  |
|  |  | 24 |  |  |  |  |  |  |  |
|  |  | 22 |  |  |  |  |  |  |  |
|  | *Dolichousnea longissima* | 10 |  |  |  |  |  |  |  |
|  |  | 12 |  |  |  |  |  |  |  |
|  |  | 12 |  |  |  |  |  |  |  |
|  |  | 14 |  |  |  |  |  |  |  |
|  | *Flavoparmelia caperata* | 13 |  |  |  |  |  |  |  |
|  |  | 11.6 |  |  |  |  |  |  |  |
|  |  | 21 |  |  |  |  |  |  |  |
|  |  | 7.6 |  |  |  |  |  |  |  |
|  |  | 18.3 |  |  |  |  |  |  |  |
|  |  | 13.3 |  |  |  |  |  |  |  |
|  |  | 9.3 |  |  |  |  |  |  |  |
|  |  | 21 |  |  |  |  |  |  |  |
|  | *Heterodermia diademata* | 11 |  |  |  |  |  |  |  |
|  |  | 12 |  |  |  |  |  |  |  |
|  | *Hypogymnia physodes* | 19 |  |  |  |  |  |  |  |
|  |  | 16 |  |  |  |  |  |  |  |
|  | *Hypotrachyna nepalensis* | 11 |  |  |  |  |  |  |  |
|  |  | 15 |  |  |  |  |  |  |  |
|  | *Leucodermia boryi* | 12 |  |  |  |  |  |  |  |
|  |  | 6 |  |  |  |  |  |  |  |
|  |  | 21 |  |  |  |  |  |  |  |
|  |  | 20 |  |  |  |  |  |  |  |
|  |  | 14 |  |  |  |  |  |  |  |
|  | *Menegazzia terebrata* | 21 |  |  |  |  |  |  |  |
|  |  | 21 |  |  |  |  |  |  |  |
|  | *Ochrolechia androgyna* | 20 |  |  |  |  |  |  |  |
|  |  | 9 |  |  |  |  |  |  |  |
|  | *Parmelia omphalodes* | 15 |  |  |  |  |  |  |  |
|  |  | 18 |  |  |  |  |  |  |  |
|  | *Parmelia saxatilis* | 13 |  |  |  |  |  |  |  |
|  |  | 17 |  |  |  |  |  |  |  |
|  | *Parmelia sulcata* | 12 |  |  |  |  |  |  |  |
|  |  | 14 |  |  |  |  |  |  |  |
|  |  | 11 |  |  |  |  |  |  |  |
|  |  | 16 |  |  |  |  |  |  |  |
|  | *Parmeliopsis ambigua* | 12 |  |  |  |  |  |  |  |
|  |  | 17 |  |  |  |  |  |  |  |
|  | *Parmotrema andinum* | 5.33 |  |  |  |  |  |  |  |
|  |  | 19.66 |  |  |  |  |  |  |  |
|  | *Parmotrema austrosinense* | 12.3 |  |  |  |  |  |  |  |
|  |  | 14.6 |  |  |  |  |  |  |  |
|  |  | 21.3 |  |  |  |  |  |  |  |
|  | *Parmotrema grayanum* | 11.6 |  |  |  |  |  |  |  |
|  | *Parmeliopsis hyperopta* | 12 |  |  |  |  |  |  |  |
|  |  | 12 |  |  |  |  |  |  |  |
|  |  | 16 |  |  |  |  |  |  |  |
|  |  | 16 |  |  |  |  |  |  |  |
|  | *Parmotrema crinitum* | 12 |  |  |  |  |  |  |  |
|  | *Parmotrema perlatum* | 30 |  |  |  |  |  |  |  |
|  |  | 20 |  |  |  |  |  |  |  |
|  |  | 11 |  |  |  |  |  |  |  |
|  |  | 26 |  |  |  |  |  |  |  |
|  |  | 17 |  |  |  |  |  |  |  |
|  | *Parmotrema reticulatum* | 15 |  |  |  |  |  |  |  |
|  |  | 11.6 |  |  |  |  |  |  |  |
|  |  | 8 |  |  |  |  |  |  |  |
|  |  | 19 |  |  |  |  |  |  |  |
|  |  | 10 |  |  |  |  |  |  |  |
|  | *Parmotrema tinctorum* | 15.6 |  |  |  |  |  |  |  |
|  |  | 6.3 |  |  |  |  |  |  |  |
|  |  | 14.3 |  |  |  |  |  |  |  |
|  |  | 11.3 |  |  |  |  |  |  |  |
|  |  | 17.6 |  |  |  |  |  |  |  |
|  |  | 7.33 |  |  |  |  |  |  |  |
|  | *Parmotrema thomsonii* | 20 |  |  |  |  |  |  |  |
|  |  | 6 |  |  |  |  |  |  |  |
|  |  | 7 |  |  |  |  |  |  |  |
|  | *Physcia aipolia* | 14 |  |  |  |  |  |  |  |
|  |  | 12.3 |  |  |  |  |  |  |  |
|  |  | 12 |  |  |  |  |  |  |  |
|  |  | 12.6 |  |  |  |  |  |  |  |
|  | *Physcia caesia* | 16 |  |  |  |  |  |  |  |
|  |  | 18 |  |  |  |  |  |  |  |
|  | *Roccella montagnei* | 12.6 |  |  |  |  |  |  |  |
|  |  | 14 |  |  |  |  |  |  |  |
|  |  | 32 |  |  |  |  |  |  |  |
|  |  | 11.3 |  |  |  |  |  |  |  |
|  |  | 24 |  |  |  |  |  |  |  |
|  |  | 13 |  |  |  |  |  |  |  |
|  |  | 25 |  |  |  |  |  |  |  |
|  | *Sticta weigelli* | 15 |  |  |  |  |  |  |  |
|  |  | 8 |  |  |  |  |  |  |  |
|  | *Teloschistes flavicans* | 16.6 |  |  |  |  |  |  |  |
|  | *Vulpicida pinastri* | 18 |  |  |  |  |  |  |  |
| **IZs of crude extracts from corticolous (variant 1) lichen species *vs* IZs of crude extracts from saxicolous (variant 2) lichen species on *Fusarium oxysporum*** | | | | | | | | | |
| No. 2 | *Alectoria sarmentosa* | 18 | *Arctoparmelia centrifuga* | 8 | N = 93  $\bar{x}$ = 14.95  SD = 5.36  Me = 14 | N = 19  $\bar{x}$ = 13.89  SD = 6.15  Me = 12 | [λ] p=not significant  **[*X*^2^] p=0.07174** | [λ-L] p=not significant  [S-W] p=0.0050 | [U] p=0.124533  [λ] p>0.1 |
|  |  | 15 | *Aspicilia cinerea* | 12 |  |  |  |  |  |
|  | *Bulbothrix setschwanensis* | 10 |  | 11 |  |  |  |  |  |
|  |  | 12 | *Dermatocarpon vellereum* | 10 |  |  |  |  |  |
|  |  | 6 |  | 18 |  |  |  |  |  |
|  | *Cetrelia braunsiana* | 22 |  | 8 |  |  |  |  |  |
|  |  | 25 | *Lecanora frustulosa* | 15 |  |  |  |  |  |
|  |  | 24 |  | 15 |  |  |  |  |  |
|  |  | 22 | *Ochrolechia tartarea* | 7 |  |  |  |  |  |
|  | *Dolichousnea longissima* | 10 | *Protoparmeliopsis muralis* | 14 |  |  |  |  |  |
|  |  | 12 | *Tephromela atra* | 12 |  |  |  |  |  |
|  |  | 12 |  | 12 |  |  |  |  |  |
|  |  | 14 |  | 18 |  |  |  |  |  |
|  | *Flavoparmelia caperata* | 13 |  | 18 |  |  |  |  |  |
|  |  | 11.6 | *Umbilicaria crustulosa* | 8 |  |  |  |  |  |
|  |  | 21 |  | 11 |  |  |  |  |  |
|  |  | 7.6 | *Umbilicaria nylanderiana* | 33 |  |  |  |  |  |
|  |  | 18.3 | *Umbilicaria polyphylla* | 12 |  |  |  |  |  |
|  |  | 13.3 |  | 22 |  |  |  |  |  |
|  |  | 9.3 |  |  |  |  |  |  |  |
|  |  | 21 |  |  |  |  |  |  |  |
|  | *Heterodermia diademata* | 11 |  |  |  |  |  |  |  |
|  |  | 12 |  |  |  |  |  |  |  |
|  | *Hypogymnia physodes* | 19 |  |  |  |  |  |  |  |
|  |  | 16 |  |  |  |  |  |  |  |
|  | *Hypotrachyna nepalensis* | 11 |  |  |  |  |  |  |  |
|  |  | 15 |  |  |  |  |  |  |  |
|  | *Leucodermia boryi* | 12 |  |  |  |  |  |  |  |
|  |  | 6 |  |  |  |  |  |  |  |
|  |  | 21 |  |  |  |  |  |  |  |
|  |  | 20 |  |  |  |  |  |  |  |
|  | *Menegazzia terebrata* | 14 |  |  |  |  |  |  |  |
|  |  | 21 |  |  |  |  |  |  |  |
|  |  | 21 |  |  |  |  |  |  |  |
|  | *Ochrolechia androgyna* | 20 |  |  |  |  |  |  |  |
|  |  | 9 |  |  |  |  |  |  |  |
|  | *Parmelia omphalodes* | 15 |  |  |  |  |  |  |  |
|  |  | 18 |  |  |  |  |  |  |  |
|  | *Parmelia saxatilis* | 13 |  |  |  |  |  |  |  |
|  |  | 17 |  |  |  |  |  |  |  |
|  | *Parmelia sulcata* | 12 |  |  |  |  |  |  |  |
|  |  | 14 |  |  |  |  |  |  |  |
|  |  | 11 |  |  |  |  |  |  |  |
|  |  | 16 |  |  |  |  |  |  |  |
|  | *Parmeliopsis ambigua* | 12 |  |  |  |  |  |  |  |
|  |  | 17 |  |  |  |  |  |  |  |
|  | *Parmotrema andinum* | 5.33 |  |  |  |  |  |  |  |
|  |  | 19.66 |  |  |  |  |  |  |  |
|  | *Parmotrema austrosinense* | 12.3 |  |  |  |  |  |  |  |
|  |  | 14.6 |  |  |  |  |  |  |  |
|  |  | 21.3 |  |  |  |  |  |  |  |
|  | *Parmotrema grayanum* | 11.6 |  |  |  |  |  |  |  |
|  | *Parmeliopsis hyperopta* | 12 |  |  |  |  |  |  |  |
|  |  | 12 |  |  |  |  |  |  |  |
|  |  | 16 |  |  |  |  |  |  |  |
|  |  | 16 |  |  |  |  |  |  |  |
|  | *Parmotrema crinitum* | 12 |  |  |  |  |  |  |  |
|  | *Parmotrema perlatum* | 30 |  |  |  |  |  |  |  |
|  |  | 20 |  |  |  |  |  |  |  |
|  |  | 11 |  |  |  |  |  |  |  |
|  |  | 26 |  |  |  |  |  |  |  |
|  |  | 17 |  |  |  |  |  |  |  |
|  | *Parmotrema reticulatum* | 15 |  |  |  |  |  |  |  |
|  |  | 11.6 |  |  |  |  |  |  |  |
|  |  | 8 |  |  |  |  |  |  |  |
|  |  | 19 |  |  |  |  |  |  |  |
|  |  | 10 |  |  |  |  |  |  |  |
|  | *Parmotrema tinctorum* | 15.6 |  |  |  |  |  |  |  |
|  |  | 6.3 |  |  |  |  |  |  |  |
|  |  | 14.3 |  |  |  |  |  |  |  |
|  |  | 11.3 |  |  |  |  |  |  |  |
|  |  | 17.6 |  |  |  |  |  |  |  |
|  |  | 7.33 |  |  |  |  |  |  |  |
|  | *Parmotrema thomsonii* | 20 |  |  |  |  |  |  |  |
|  |  | 6 |  |  |  |  |  |  |  |
|  |  | 7 |  |  |  |  |  |  |  |
|  | *Physcia aipolia* | 14 |  |  |  |  |  |  |  |
|  |  | 12.3 |  |  |  |  |  |  |  |
|  |  | 12 |  |  |  |  |  |  |  |
|  |  | 12.6 |  |  |  |  |  |  |  |
|  | *Physcia caesia* | 16 |  |  |  |  |  |  |  |
|  |  | 18 |  |  |  |  |  |  |  |
|  | *Roccella montagnei* | 12.6 |  |  |  |  |  |  |  |
|  |  | 14 |  |  |  |  |  |  |  |
|  |  | 32 |  |  |  |  |  |  |  |
|  |  | 11.3 |  |  |  |  |  |  |  |
|  |  | 24 |  |  |  |  |  |  |  |
|  |  | 13 |  |  |  |  |  |  |  |
|  |  | 25 |  |  |  |  |  |  |  |
|  | *Sticta weigelli* | 15 |  |  |  |  |  |  |  |
|  |  | 8 |  |  |  |  |  |  |  |
|  | *Teloschistes flavicans* | 16.6 |  |  |  |  |  |  |  |
|  | *Vulpicida pinastri* | 18 |  |  |  |  |  |  |  |
| **IZs of crude extracts from terricolous (variant 1) lichen species *vs* IZs of crude extracts from saxicolous (variant 2) lichen species on *Fusarium oxysporum*** | | | | | | | | | |
| No. 3 | *Cladonia furcata* | 11 | *Arctoparmelia centrifuga* | 8 | N = 5  $\bar{x}$ = 12.4  SD = 1.14  Me = 12 | N = 19  $\bar{x}$ = 13.89  SD = 6.15  Me = 12 | [λ-L] p=not significant  **[S-W] p=0.8140** | [λ-L] p=not significant  [S-W] p=0.0050 | [U] p=0.971342  [λ] p>0.1 |
|  |  | 12 | *Aspicilia cinerea* | 12 |  |  |  |  |  |
|  | *Cladonia rangiferina* | 14 |  | 11 |  |  |  |  |  |
|  |  | 13 | *Dermatocarpon vellereum* | 10 |  |  |  |  |  |
|  | *Gyalolechia fulgens* | 12 |  | 18 |  |  |  |  |  |
|  |  |  |  | 8 |  |  |  |  |  |
|  |  |  | *Lecanora frustulosa* | 15 |  |  |  |  |  |
|  |  |  |  | 15 |  |  |  |  |  |
|  |  |  | *Ochrolechia tartarea* | 7 |  |  |  |  |  |
|  |  |  | *Protoparmeliopsis muralis* | 14 |  |  |  |  |  |
|  |  |  | *Tephromela atra* | 12 |  |  |  |  |  |
|  |  |  |  | 12 |  |  |  |  |  |
|  |  |  |  | 18 |  |  |  |  |  |
|  |  |  |  | 18 |  |  |  |  |  |
|  |  |  | *Umbilicaria crustulosa* | 8 |  |  |  |  |  |
|  |  |  |  | 11 |  |  |  |  |  |
|  |  |  | *Umbilicaria nylanderiana* | 33 |  |  |  |  |  |
|  |  |  | *Umbilicaria polyphylla* | 12 |  |  |  |  |  |
|  |  |  |  | 22 |  |  |  |  |  |

**Table S18.** Comparative statistical analysis between two sets of Minimal Inhibitory Concentration (MIC) values for extracts from corticolous, terricolous and saxicolous lichen species and lichen secondary metabolites against *Fusarium oxysporum* based on literature data. Normality of data distribution was checked by Shapiro-Wilk
and λ Kolmogorov-Smirnov with Lilliefors’s amendment tests (N<30), and *X*^2^ and λ Kolmogorov-Smirnov tests (N≥30). Because the assumption of normality of the data distribution was not met, homogeneity of variance tests were not performed. The analysis of statistical significance was checked by U Mann-Whitney and the λ Kolmogorov-Smirnov tests. A significance level of α=0.05 was assumed for all tests. In red the compared variant with statistically significantly lower (methodologically beneficial) difference in mean MIC value is marked. Abbreviations: $\bar{x}$ = MIC arithmetic mean; SD = MIC Standard Deviation; Me = median; [S-W] = Shapiro-Wilk test; [*X*^2^] = *X*^2^ test; [λ] = Kolmogorov-Smirnov test; [λ-L] = Kolmogorov-Smirnov with Lilliefors’s amendment test; [U] = Mann-Whitney test. For literature references: see adequate Tab. S6-S8 & S12.

| **Group no.** | **Methodological combinations compared** | | | | **Variants general data** | | **Tests of distribution normality**  **(normal distribution in bold)** | | **Statistical tests** |
| --- | --- | --- | --- | --- | --- | --- | --- | --- | --- |
|  | **Variant 1** | | **Variant 2** | | **Variant 1** | **Variant 2** | **Variant 1** | **Variant 2** |  |
| **MICs of crude extracts from corticolous lichen extracts (variant 1) *vs* MICs of lichen secondary metabolites (variant 2) on *Fusarium oxysporum*** | | | | | | | | | |
|  | **Lichen species** | **MIC [mg·ml^-1^]** | **Lichen secondary metabolites** | **MIC**  **[mg·ml^-1^]** | N = 65  $\bar{x}$ = 12.47  SD = 17.47  Me = 6.25 | **N = 25**  $\bar{\boldsymbol{x}}$ **= 1.88**  **SD = 2.74**  **Me = 0.5** | [λ] p<0.01  [*X*^2^] p=0.00000 | [λ-L] p<0.01  [S-W] p=0.00001 | **[U] p=0.000001**  **[λ] p<0.001** |
| No. 1 | *Alectoria sarmentosa* | 50 | divaricatic acid | 6.25 |  |  |  |  |  |
|  |  | 100 |  | 6.25 |  |  |  |  |  |
|  | *Flavoparmelia caperata* | 1.562 | lecanoric accid | 0.125 |  |  |  |  |  |
|  |  | 6.25 |  | 1 |  |  |  |  |  |
|  |  | 1.56 | olivetoric acid | 10  [= 500 µg·50 µl^-1^] |  |  |  |  |  |
|  |  | 25 | methyl evernate | 0.5 |  |  |  |  |  |
|  |  | 6.25 | 2'-O-methylanziaic acid | 0.25 |  |  |  |  |  |
|  |  | 1.875 | obtusatic acid | 1 |  |  |  |  |  |
|  |  | 0.097 | atranorin | 16 × 10^-3^ |  |  |  |  |  |
|  |  | 6.25 |  | 0.5 |  |  |  |  |  |
|  |  | 1.56 | chloroatranorin | 7.5  [= 300 µg·40 µl^-1^] |  |  |  |  |  |
|  | *Hypogymnia physodes* | 12.5 | 2-hydroxy-4-methoxy-3,6-dimethylbenzoic acid | 4 × 10^-3^ |  |  |  |  |  |
|  |  | 12.5 | physodic acid | 1 |  |  |  |  |  |
|  |  | 12.5 |  | 0.25 |  |  |  |  |  |
|  |  | 6.25 | fumarprotocetraric acid | 0.25 |  |  |  |  |  |
|  |  | 6.25 |  | 0.25 |  |  |  |  |  |
|  | *Melanelixia fuliginosa* | 2.5 | protocetraric acid | 0.5 |  |  |  |  |  |
|  | *Melanelixia subaurifera* | 10 | salazinic acid | 3.12 |  |  |  |  |  |
|  | *Menegazzia terebrata* | 1.56 | stictic acid | 1 |  |  |  |  |  |
|  |  | 6.25 | gyrophororic acid | 0.25 |  |  |  |  |  |
|  |  | 1.56 |  | 0.25 |  |  |  |  |  |
|  | *Ochrolechia androgyna* | 3.12 | hopane-6α, 22-diol (zeorin) | 3.12 |  |  |  |  |  |
|  |  | 50 |  | 3.12 |  |  |  |  |  |
|  | *Parmelia omphalodes* | 3.12 | usnic acid | 0.5 |  |  |  |  |  |
|  |  | 3.12 | (+)-usnic acid | 16 × 10^-3^ |  |  |  |  |  |
|  | *Parmelia saxatilis* | 25 |  |  |  |  |  |  |  |
|  |  | 25 |  |  |  |  |  |  |  |
|  |  | 12.5 |  |  |  |  |  |  |  |
|  | *Parmelia sulcata* | 1.56 |  |  |  |  |  |  |  |
|  |  | 25 |  |  |  |  |  |  |  |
|  |  | 6.25 |  |  |  |  |  |  |  |
|  |  | 25 |  |  |  |  |  |  |  |
|  |  | 3.12 |  |  |  |  |  |  |  |
|  | *Parmeliopsis ambigua* | 25 |  |  |  |  |  |  |  |
|  |  | 25 |  |  |  |  |  |  |  |
|  |  | 6.25 |  |  |  |  |  |  |  |
|  | *Parmotrema austrosinense* | 1.562 |  |  |  |  |  |  |  |
|  |  | 3.125 |  |  |  |  |  |  |  |
|  |  | 0.390 |  |  |  |  |  |  |  |
|  | *Parmotrema grayanum* | 3.125 |  |  |  |  |  |  |  |
|  | *Parmeliopsis hyperopta* | 12.5 |  |  |  |  |  |  |  |
|  |  | 12.5 |  |  |  |  |  |  |  |
|  |  | 3.12 |  |  |  |  |  |  |  |
|  |  | 3.12 |  |  |  |  |  |  |  |
|  | *Parmotrema crinitum* | 15 |  |  |  |  |  |  |  |
|  | *Parmotrema reticulatum* | 12.5 |  |  |  |  |  |  |  |
|  |  | 50 |  |  |  |  |  |  |  |
|  |  | 50 |  |  |  |  |  |  |  |
|  |  | 6.25 |  |  |  |  |  |  |  |
|  |  | 50 |  |  |  |  |  |  |  |
|  | *Parmotrema tinctorum* | 3.125 |  |  |  |  |  |  |  |
|  |  | 6.25 |  |  |  |  |  |  |  |
|  | *Physcia aipolia* | 6.25 |  |  |  |  |  |  |  |
|  |  | 0.39 |  |  |  |  |  |  |  |
|  |  | 25 |  |  |  |  |  |  |  |
|  |  | 0.39 |  |  |  |  |  |  |  |
|  | *Physcia ceasia* | 6.25 |  |  |  |  |  |  |  |
|  |  | 3.12 |  |  |  |  |  |  |  |
|  | *Ramalina fastigiata* | 2.5 |  |  |  |  |  |  |  |
|  | *Ramalina fraxinea* | 5 |  |  |  |  |  |  |  |
|  | *Roccella montagnei* | 3.125 |  |  |  |  |  |  |  |
|  |  | 0.39 |  |  |  |  |  |  |  |
|  |  | 0.39 |  |  |  |  |  |  |  |
|  | *Teloschistes flavicans* | 6.25 |  |  |  |  |  |  |  |
|  | *Vulpicida pinastri* | 7.5 |  |  |  |  |  |  |  |
| **MICs of crude extracts from terricolous lichen extracts (variant 1) *vs* MICs of lichen secondary metabolites (variant 2) on *Fusarium oxysporum*** | | | | | | | | | |
| No. 2 | *Cetraria islandica* | 2.5 | divaricatic acid | 6.25 | N = 14  $\bar{x}$ = 31.96  SD = 41.67  Me = 25 | **N = 25**  $\bar{\boldsymbol{x}}$ **= 1.88**  **SD = 2.74**  **Me = 0.5** | [λ-L] p<0.01  [S-W] p=0.0001 | [λ-L] p<0.01  [S-W] p=0.00001 | **[U] p=0.000003**  **[λ] p<0.001** |
|  | *Cladonia fimbriata* | 5 |  | 6.25 |  |  |  |  |  |
|  | *Cladonia foliacea* | 10 | lecanoric accid | 0.125 |  |  |  |  |  |
|  | *Cladonia furcata* | 25 |  | 1 |  |  |  |  |  |
|  |  | 25 | olivetoric acid | 10  [= 500 µg·50 µl^-1^] |  |  |  |  |  |
|  |  | 25 | methyl evernate | 0.5 |  |  |  |  |  |
|  |  | 10 | 2'-O-methylanziaic acid | 0.25 |  |  |  |  |  |
|  |  | 25 | obtusatic acid | 1 |  |  |  |  |  |
|  |  | 25 | atranorin | 16 × 10^-3^ |  |  |  |  |  |
|  | *Cladonia rangiferina* | 10 |  | 0.5 |  |  |  |  |  |
|  |  | 100 | chloroatranorin | 7.5  [= 300 µg·40µl^-1^] |  |  |  |  |  |
|  |  | 150 | 2-hydroxy-4-methoxy-3,6-dimethylbenzoic acid | 4 × 10^-3^ |  |  |  |  |  |
|  | *Gyalolechia fulgens* | 30 | physodic acid | 1 |  |  |  |  |  |
|  | *Stereocaulon paschale* | 5 |  | 0.25 |  |  |  |  |  |
|  |  |  | fumarprotocetraric acid | 0.25 |  |  |  |  |  |
|  |  |  |  | 0.25 |  |  |  |  |  |
|  |  |  | protocetraric acid | 0.5 |  |  |  |  |  |
|  |  |  | salazinic acid | 3.12 |  |  |  |  |  |
|  |  |  | stictic acid | 1 |  |  |  |  |  |
|  |  |  | gyrophororic acid | 0.25 |  |  |  |  |  |
|  |  |  |  | 0.25 |  |  |  |  |  |
|  |  |  | hopane-6α, 22-diol (zeorin) | 3.12 |  |  |  |  |  |
|  |  |  |  | 3.12 |  |  |  |  |  |
|  |  |  | usnic acid | 0.5 |  |  |  |  |  |
|  |  |  | (+)-usnic acid | 16 × 10^-3^ |  |  |  |  |  |
| **MICs of crude extracts from saxicolous lichen extracts (variant 1) *vs* MICs of lichen secondary metabolites (variant 2) on *Fusarium oxysporum*** | | | | | | | | | |
| No. 3 | *Arctoparmelia centrifuga* | 30 | divaricatic acid | 6.25 | N = 22  $\bar{x}$ = 15.88  SD = 10.18  Me = 12.5 | **N = 25**  $\bar{\boldsymbol{x}}$ **= 1.88**  **SD = 2.74**  **Me = 0.5** | [λ-L] p<0.01  [S-W] p=0.0072 | [λ-L] p<0.01  [S-W] p=0.00001 | **[U] p=0.000001**  **[λ] p<0.01** |
|  | *Aspicilia cinerea* | 25 |  | 6.25 |  |  |  |  |  |
|  |  | 25 | lecanoric accid | 0.125 |  |  |  |  |  |
|  | *Lasallia pustulata* | 5 |  | 1 |  |  |  |  |  |
|  | *Lecanora frustulosa* | 12.5 | olivetoric acid | 10  [= 500 µg·50 µl^-1^] |  |  |  |  |  |
|  |  | 12.5 | methyl evernate | 0.5 |  |  |  |  |  |
|  | *Ochrolechia tarterea* | 30 | 2'-O-methylanziaic acid | 0.25 |  |  |  |  |  |
|  | *Protoparmeliopsis muralis* | 25 | obtusatic acid | 1 |  |  |  |  |  |
|  | *Tephromela atra* | 25 | atranorin | 16 × 10^-3^ |  |  |  |  |  |
|  |  | 25 |  | 0.5 |  |  |  |  |  |
|  |  | 25 | chloroatranorin | 7.5  [= 300 µg·40 µl^-1^] |  |  |  |  |  |
|  |  | 6.25 | 2-hydroxy-4-methoxy-3,6-dimethylbenzoic acid | 4 × 10^-3^ |  |  |  |  |  |
|  |  | 6.25 | physodic acid | 1 |  |  |  |  |  |
|  | *Umbilicaria crustulosa* | 25 |  | 0.25 |  |  |  |  |  |
|  |  | 25 | fumarprotocetraric acid | 0.25 |  |  |  |  |  |
|  |  | 6.25 |  | 0.25 |  |  |  |  |  |
|  | *Umbilicaria nylanderiana* | 62.5 × 10^-3^ | protocetraric acid | 0.5 |  |  |  |  |  |
|  | *Umbilicaria polyphylla* | 12.5 | salazinic acid | 3.12 |  |  |  |  |  |
|  |  | 12.5 | stictic acid | 1 |  |  |  |  |  |
|  |  | 12.5 | gyrophororic acid | 0.25 |  |  |  |  |  |
|  |  | 1.56 |  | 0.25 |  |  |  |  |  |
|  |  | 1.56 | hopane-6α, 22-diol (zeorin) | 3.12 |  |  |  |  |  |
|  |  |  |  | 3.12 |  |  |  |  |  |
|  |  |  | usnic acid | 0.5 |  |  |  |  |  |
|  |  |  | (+)-usnic acid | 16 × 10^-3^ |  |  |  |  |  |

**Table S19.** Comparative statistical analysis between two sets of Minimal Inhibitory Concentration (MIC) values for extracts from corticolous, terricolous and saxicolous lichen species and antifungal non-lichen substances against *Fusarium oxysporum* based on literature data. Normality of data distribution was checked by Shapiro-Wilk and
λ Kolmogorov-Smirnov with a Lilliefors’s amendment tests (N<30), and *X*^2^ and λ Kolmogorov-Smirnov tests (N≥30). Because the assumption of normality of data distribution was not met, homogeneity of variance tests were not performed. Statistical significance was tested by U Mann-Whitney and λ Kolmogorov-Smirnov tests. A significance level of α=0.05 was assumed for all tests. The compared variant with statistically significantly lower (methodologically beneficial) difference in mean MIC value is marked in red. Abbreviations: $\bar{x}$ = MIC arithmetic mean; SD = MIC Standard Deviation; Me = median; [S-W] = Shapiro-Wilk test; [*X*^2^] = *X*^2^ test;
[λ] = Kolmogorov-Smirnov test; [λ-L] = Kolmogorov-Smirnov with a Lilliefors’s amendment test [U] = Mann-Whitney test. For literature references: see adequate Tab. S6-S8 & S15A.

| **Group no.** | **Methodological combinations compared** | | | | **Variants general data** | | **Tests of distribution normality**  **(normal distribution in bold)** | | **Statistical tests** |
| --- | --- | --- | --- | --- | --- | --- | --- | --- | --- |
|  | **Variant 1** | | **Variant 2** | | **Variant 1** | **Variant 2** | **Variant 1** | **Variant 2** |  |
| **MICs of crude extracts from corticolous lichen extracts (variant 1) *vs* MICs of non-lichen antifungal substances (variant 2) on *Fusarium oxysporum*** | | | | | | | | | |
|  | **Lichen species** | **MIC [mg·ml^-1^]** | **Non-lichen antifungal substances** | **MIC**  **[mg·ml^-1^]** | N = 65  $\bar{x}$ = 12.47  SD = 17.47  Me = 6.25 | **N = 8**  $\bar{\boldsymbol{x}}$ **= 0.017**  **SD = 0.028**  **Me = 0.002** | [λ] p<0.01  [*X*^2^] p=0.00000 | [λ-L] p<0.01  [S-W] p=0.0002 | **[U] p=0.000004**  **[λ] p<0.001** |
| No. 1 | *Alectoria sarmentosa* | 50 | Amphotericin B | 62.5 × 10^-3^ |  |  |  |  |  |
|  |  | 100 |  | 62.5 × 10^-3^ |  |  |  |  |  |
|  | *Flavoparmelia caperata* | 1.562 |  | 2.13 × 10^-3^ |  |  |  |  |  |
|  |  | 6.25 |  | 5.65 × 10^-3^ |  |  |  |  |  |
|  |  | 1.56 |  | 0.12 × 10^-3^ |  |  |  |  |  |
|  |  | 25 |  | 2 × 10^-3^ |  |  |  |  |  |
|  |  | 6.25 |  | 0.5 × 10^-3^ |  |  |  |  |  |
|  |  | 1.875 |  | 2 × 10^-3^ |  |  |  |  |  |
|  |  | 0.097 | Ketoconazole | 3.75 × 10^-3^ |  | **N = 24**  $\bar{\boldsymbol{x}}$ **= 0.16**  **SD = 0.026**  **Me = 0.004** |  | [λ-L] p<0.01  [S-W] p=0.00000 | **[U] p=0.000000**  **[λ] p<0.001** |
|  |  | 6.25 |  | 3.75 × 10^-3^ |  |  |  |  |  |
|  |  | 1.56 |  | 3.9 × 10^-3^  [x 17 references] |  |  |  |  |  |
|  | *Hypogymnia physodes* | 12.5 |  | 78 × 10^-3^  [x 3 references] |  |  |  |  |  |
|  |  | 12.5 |  | 33.26 × 10^-3^ |  |  |  |  |  |
|  |  | 12.5 |  | 43.54 × 10^-3^ |  |  |  |  |  |
|  |  | 6.25 | Terbinafine | 15 × 10^-3^ |  | **N = 5**  $\bar{\boldsymbol{x}}$ **= 0.0115**  **SD = 0.019**  **Me = 0.008** |  | [λ-L] p=not significant  **[S-W] p=0.3253** | **[U] p=0.000203**  **[λ] p<0.001** |
|  |  | 6.25 |  | 0.5 × 10^-3^ |  |  |  |  |  |
|  | *Melanelixia fuliginosa* | 2.5 |  | 32 × 10^-3^ |  |  |  |  |  |
|  | *Melanelixia subaurifera* | 10 |  | 2 × 10^-3^ |  |  |  |  |  |
|  | *Menegazzia terebrata* | 1.56 |  | 8 × 10^-3^ |  |  |  |  |  |
|  |  | 6.25 |  |  |  |  |  |  |  |
|  |  | 1.56 |  |  |  |  |  |  |  |
|  | *Ochrolechia androgyna* | 3.12 |  |  |  |  |  |  |  |
|  |  | 50 |  |  |  |  |  |  |  |
|  | *Parmelia omphalodes* | 3.12 |  |  |  |  |  |  |  |
|  |  | 3.12 |  |  |  |  |  |  |  |
|  | *Parmelia saxatilis* | 25 |  |  |  |  |  |  |  |
|  |  | 25 |  |  |  |  |  |  |  |
|  |  | 12.5 |  |  |  |  |  |  |  |
|  | *Parmelia sulcata* | 1.56 |  |  |  |  |  |  |  |
|  |  | 25 |  |  |  |  |  |  |  |
|  |  | 6.25 |  |  |  |  |  |  |  |
|  |  | 25 |  |  |  |  |  |  |  |
|  |  | 3.12 |  |  |  |  |  |  |  |
|  | *Parmeliopsis ambigua* | 25 |  |  |  |  |  |  |  |
|  |  | 25 |  |  |  |  |  |  |  |
|  |  | 6.25 |  |  |  |  |  |  |  |
|  | *Parmotrema austrosinense* | 1.562 |  |  |  |  |  |  |  |
|  |  | 3.125 |  |  |  |  |  |  |  |
|  |  | 0.390 |  |  |  |  |  |  |  |
|  | *Parmotrema grayanum* | 3.125 |  |  |  |  |  |  |  |
|  | *Parmeliopsis hyperopta* | 12.5 |  |  |  |  |  |  |  |
|  |  | 12.5 |  |  |  |  |  |  |  |
|  |  | 3.12 |  |  |  |  |  |  |  |
|  |  | 3.12 |  |  |  |  |  |  |  |
|  | *Parmotrema crinitum* | 15 |  |  |  |  |  |  |  |
|  | *Parmotrema reticulatum* | 12.5 |  |  |  |  |  |  |  |
|  |  | 50 |  |  |  |  |  |  |  |
|  |  | 50 |  |  |  |  |  |  |  |
|  |  | 6.25 |  |  |  |  |  |  |  |
|  |  | 50 |  |  |  |  |  |  |  |
|  | *Parmotrema tinctorum* | 3.125 |  |  |  |  |  |  |  |
|  |  | 6.25 |  |  |  |  |  |  |  |
|  | *Physcia aipolia* | 6.25 |  |  |  |  |  |  |  |
|  |  | 0.39 |  |  |  |  |  |  |  |
|  |  | 25 |  |  |  |  |  |  |  |
|  |  | 0.39 |  |  |  |  |  |  |  |
|  | *Physcia ceasia* | 6.25 |  |  |  |  |  |  |  |
|  |  | 3.12 |  |  |  |  |  |  |  |
|  | *Ramalina fastigiata* | 2.5 |  |  |  |  |  |  |  |
|  | *Ramalina fraxinea* | 5 |  |  |  |  |  |  |  |
|  | *Roccella montagnei* | 3.125 |  |  |  |  |  |  |  |
|  |  | 0.39 |  |  |  |  |  |  |  |
|  |  | 0.39 |  |  |  |  |  |  |  |
|  | *Teloschistes flavicans* | 6.25 |  |  |  |  |  |  |  |
|  | *Vulpicida pinastri* | 7.5 |  |  |  |  |  |  |  |
| **MICs of crude extracts from terricolous lichen extracts (variant 1) *vs* MICs of non-lichen antifungal substances (variant 2) on *Fusarium oxysporum*** | | | | | | | | | |
| No. 2 | *Cetraria islandica* | 2.5 | Amphotericin B | 62.5 × 10^-3^ | N= 14  $\bar{x}$ = 31.96  SD = 41.67  Me = 25 | **N = 8**  $\bar{\boldsymbol{x}}$ **= 0.017**  **SD = 0.028**  **Me = 0.002** | [λ-L] p<0.01  [S-W] p=0.0001 | [λ-L] p<0.01  [S-W] p=0.0002 | **[U] p=0.000152**  **[λ] p<0.001** |
|  | *Cladonia fimbriata* | 5 |  | 62.5 × 10^-3^ |  |  |  |  |  |
|  | *Cladonia foliacea* | 10 |  | 2.13 × 10^-3^ |  |  |  |  |  |
|  | *Cladonia furcata* | 25 |  | 5.65 × 10^-3^ |  |  |  |  |  |
|  |  | 25 |  | 0.12 × 10^-3^ |  |  |  |  |  |
|  |  | 25 |  | 2 × 10^-3^ |  |  |  |  |  |
|  |  | 10 |  | 0.5 × 10^-3^ |  |  |  |  |  |
|  |  | 25 |  | 2 × 10^-3^ |  |  |  |  |  |
|  |  | 25 | Ketoconazole | 3.75 × 10^-3^ |  | **N = 24**  $\bar{\boldsymbol{x}}$ **= 0.16**  **SD = 0.026**  **Me = 0.004** |  | [λ-L] p<0.01  [S-W] p=0.00000 | **[U] p=0.000000**  **[λ] p<0.001** |
|  | *Cladonia rangiferina* | 10 |  | 3.75 × 10^-3^ |  |  |  |  |  |
|  |  | 100 |  | 3.9 × 10^-3^  [x 17 references] |  |  |  |  |  |
|  |  | 150 |  | 78 × 10^-3^  [x 3 references] |  |  |  |  |  |
|  | *Gyalolechia fulgens* | 30 |  | 33.26 × 10^-3^ |  |  |  |  |  |
|  | *Stereocaulon paschale* | 5 |  | 43.54 × 10^-3^ |  |  |  |  |  |
|  |  |  | Terbinafine | 15 × 10^-3^ |  | **N = 5**  $\bar{\boldsymbol{x}}$ **= 0.0115**  **SD = 0.019**  **Me = 0.008** |  | [λ-L] p=not significant  **[S-W] p=0.3253** | **[U] p=0.001403**  **[λ] p<0.005** |
|  |  |  |  | 0.5 × 10^-3^ |  |  |  |  |  |
|  |  |  |  | 32 × 10^-3^ |  |  |  |  |  |
|  |  |  |  | 2 × 10^-3^ |  |  |  |  |  |
|  |  |  |  | 8 × 10^-3^ |  |  |  |  |  |
| **MICs of crude extracts from saxicolous lichen extracts (variant 1) *vs* MICs of non-lichen antifungal substances (variant 2) on *Fusarium oxysporum*** | | | | | | | | | |
| No. 3 | *Arctoparmelia centrifuga* | 30 | Amphotericin B | 62.5 × 10^-3^ | N = 22  $\bar{x}$ = 15.88  SD = 10.18  Me = 12.5 | **N = 8**  $\bar{\boldsymbol{x}}$ **= 0.017**  **SD = 0.028**  **Me = 0.002** | [λ-L] p<0.01  [S-W] p=0.0072 | [λ-L] p<0.01  [S-W] p=0.0002 | **[U] p=0.000040**  **[λ] p<0.001** |
|  | *Aspicilia cinerea* | 25 |  | 62.5 × 10^-3^ |  |  |  |  |  |
|  |  | 25 |  | 2.13 × 10^-3^ |  |  |  |  |  |
|  | *Lasallia pustulata* | 5 |  | 5.65 × 10^-3^ |  |  |  |  |  |
|  | *Lecanora frustulosa* | 12.5 |  | 0.12 × 10^-3^ |  |  |  |  |  |
|  |  | 12.5 |  | 2 × 10^-3^ |  |  |  |  |  |
|  | *Ochrolechia tarterea* | 30 |  | 0.5 × 10^-3^ |  |  |  |  |  |
|  | *Protoparmeliopsis muralis* | 25 |  | 2 × 10^-3^ |  |  |  |  |  |
|  | *Tephromela atra* | 25 | Ketoconazole | 3.75 × 10^-3^ |  | **N = 24**  $\bar{\boldsymbol{x}}$ **= 0.16**  **SD = 0.026**  **Me = 0.004** |  | [λ-L] p<0.01  [S-W] p=0.00000 | **[U] p=0.000000**  **[λ] p<0.001** |
|  |  | 25 |  | 3.75 × 10^-3^ |  |  |  |  |  |
|  |  | 25 |  | 3.9 × 10^-3^  [x 17 references] |  |  |  |  |  |
|  |  | 6.25 |  | 78 × 10^-3^  [x 3 references] |  |  |  |  |  |
|  |  | 6.25 |  | 33.26 × 10^-3^ |  |  |  |  |  |
|  | *Umbilicaria crustulosa* | 25 |  | 43.54 × 10^-3^ |  |  |  |  |  |
|  |  | 25 | Terbinafine | 15 × 10^-3^ |  | **N = 5**  $\bar{\boldsymbol{x}}$ **= 0.0115**  **SD = 0.019**  **Me = 0.008** |  | [λ-L] p=not significant  **[S-W] p=0.3253** | **[U] p=0.000539**  **[λ] p<0.001** |
|  |  | 6.25 |  | 0.5 × 10^-3^ |  |  |  |  |  |
|  | *Umbilicaria nylanderiana* | 62.5 × 10^-3^ |  | 32 × 10^-3^ |  |  |  |  |  |
|  | *Umbilicaria polyphylla* | 12.5 |  | 2 × 10^-3^ |  |  |  |  |  |
|  |  | 12.5 |  | 8 × 10^-3^ |  |  |  |  |  |
|  |  | 12.5 |  |  |  |  |  |  |  |
|  |  | 1.56 |  |  |  |  |  |  |  |
|  |  | 1.56 |  |  |  |  |  |  |  |

**Table S20.** Comparative statistical analysis between two sets of Inhibition Zone (IZ) values for extracts from corticolous, terricolous and saxicolous lichen species and
an antifungal non-lichen substance (ketoconazole) against *Fusarium oxysporum* based on literature data. Normality of data distribution was checked by Shapiro-Wilk and
λ Kolmogorov-Smirnov test with a Lilliefors’s amendment tests (N<30), and *X*^2^ and λ Kolmogorov-Smirnov tests (N≥30). Because the assumption of normality of data distribution was not met, homogeneity of variance tests were not performed. The analysis of statistical significance was checked by U Mann-Whitney and λ Kolmogorov-Smirnov tests. A significance level of α=0.05 was assumed for all tests. The compared variant with statistically significantly higher (methodologically beneficial) difference
in mean value of IZ is marked in red. Abbreviations: $\bar{x}$ = MIC arithmetic mean; SD = MIC Standard Deviation; Me = median; [S-W] = Shapiro-Wilk test; [*X*^2^] = *X*^2^ test;
[λ] = Kolmogorov-Smirnov test; [λ-L] = Kolmogorov-Smirnov with a Lilliefors’s amendment test; [U] = Mann-Whitney test. For literature references: see adequate Tab. S6-S8 & S15A.

| **Group no.** | **Methodological combinations compared** | | | | **Variants general data** | | **Tests of distribution normality**  **(normal distribution in bold)** | | **Statistical tests** |
| --- | --- | --- | --- | --- | --- | --- | --- | --- | --- |
|  | **Variant 1** | | **Variant 2** | | **Variant 1** | **Variant 2** | **Variant 1** | **Variant 2** |  |
|  | **IZs of crude extracts from corticolous lichen species (variant 1) *vs* IZs of non-lichen antifungal substance (variant 2) on *Fusarium oxysporum*** | | | | | | | | |
|  | **Lichen species** | **IZ**  **[mm]** | **Non-lichen antifungal substances** | **IZ**  **[mm]** | N = 93  $\bar{x}$ = 14.95  SD = 5.36  Me = 14 | **N = 17**  $\bar{\boldsymbol{x}}$ **= 25.29**  **SD = 10.30**  **Me = 30** | [λ] p=not significant  **[*X*^2^] p=0.07174** | [λ-L] p<0.01  [S-W] p=0.0009 | **[U] p=0.000274**  **[λ] p<0.005** |
| No. 1 | *Alectoria sarmentosa* | 18 | Ketoconazole | 30 |  |  |  |  |  |
|  |  | 15 |  | 35  [x 8 references] |  |  |  |  |  |
|  | *Bulbothrix setschwanensis* | 10 |  | 10 |  |  |  |  |  |
|  |  | 12 |  | 17.3 |  |  |  |  |  |
|  |  | 6 |  | 17.3 |  |  |  |  |  |
|  | *Cetrelia braunsiana* | 22 |  | 20 |  |  |  |  |  |
|  |  | 25 |  | 15.33 |  |  |  |  |  |
|  |  | 24 |  | 14 |  |  |  |  |  |
|  |  | 22 |  | 12 |  |  |  |  |  |
|  | *Dolichousnea longissima* | 10 |  | 14 |  |  |  |  |  |
|  |  | 12 |  |  |  |  |  |  |  |
|  |  | 12 |  |  |  |  |  |  |  |
|  |  | 14 |  |  |  |  |  |  |  |
|  | *Flavoparmelia caperata* | 13 |  |  |  |  |  |  |  |
|  |  | 11.6 |  |  |  |  |  |  |  |
|  |  | 21 |  |  |  |  |  |  |  |
|  |  | 7.6 |  |  |  |  |  |  |  |
|  |  | 18.3 |  |  |  |  |  |  |  |
|  |  | 13.3 |  |  |  |  |  |  |  |
|  |  | 9.3 |  |  |  |  |  |  |  |
|  |  | 21 |  |  |  |  |  |  |  |
|  | *Heterodermia diademata* | 11 |  |  |  |  |  |  |  |
|  |  | 12 |  |  |  |  |  |  |  |
|  | *Hypogymnia physodes* | 19 |  |  |  |  |  |  |  |
|  |  | 16 |  |  |  |  |  |  |  |
|  | *Hypotrachyna nepalensis* | 11 |  |  |  |  |  |  |  |
|  |  | 15 |  |  |  |  |  |  |  |
|  | *Leucodermia boryi* | 12 |  |  |  |  |  |  |  |
|  |  | 6 |  |  |  |  |  |  |  |
|  |  | 21 |  |  |  |  |  |  |  |
|  |  | 20 |  |  |  |  |  |  |  |
|  |  | 14 |  |  |  |  |  |  |  |
|  | *Menegazzia terebrata* | 21 |  |  |  |  |  |  |  |
|  |  | 21 |  |  |  |  |  |  |  |
|  | *Ochrolechia androgyna* | 20 |  |  |  |  |  |  |  |
|  |  | 9 |  |  |  |  |  |  |  |
|  | *Parmelia omphalodes* | 15 |  |  |  |  |  |  |  |
|  |  | 18 |  |  |  |  |  |  |  |
|  | *Parmelia saxatilis* | 13 |  |  |  |  |  |  |  |
|  |  | 17 |  |  |  |  |  |  |  |
|  | *Parmelia sulcata* | 12 |  |  |  |  |  |  |  |
|  |  | 14 |  |  |  |  |  |  |  |
|  |  | 11 |  |  |  |  |  |  |  |
|  |  | 16 |  |  |  |  |  |  |  |
|  | *Parmeliopsis ambigua* | 12 |  |  |  |  |  |  |  |
|  |  | 17 |  |  |  |  |  |  |  |
|  | *Parmotrema andinum* | 5.33 |  |  |  |  |  |  |  |
|  |  | 19.66 |  |  |  |  |  |  |  |
|  | *Parmotrema austrosinense* | 12.3 |  |  |  |  |  |  |  |
|  |  | 14.6 |  |  |  |  |  |  |  |
|  |  | 21.3 |  |  |  |  |  |  |  |
|  | *Parmotrema grayanum* | 11.6 |  |  |  |  |  |  |  |
|  | *Parmeliopsis hyperopta* | 12 |  |  |  |  |  |  |  |
|  |  | 12 |  |  |  |  |  |  |  |
|  |  | 16 |  |  |  |  |  |  |  |
|  |  | 16 |  |  |  |  |  |  |  |
|  | *Parmotrema crinitum* | 12 |  |  |  |  |  |  |  |
|  | *Parmotrema perlatum* | 30 |  |  |  |  |  |  |  |
|  |  | 20 |  |  |  |  |  |  |  |
|  |  | 11 |  |  |  |  |  |  |  |
|  |  | 26 |  |  |  |  |  |  |  |
|  |  | 17 |  |  |  |  |  |  |  |
|  | *Parmotrema reticulatum* | 15 |  |  |  |  |  |  |  |
|  |  | 11.6 |  |  |  |  |  |  |  |
|  |  | 8 |  |  |  |  |  |  |  |
|  |  | 19 |  |  |  |  |  |  |  |
|  |  | 10 |  |  |  |  |  |  |  |
|  | *Parmotrema tinctorum* | 15.6 |  |  |  |  |  |  |  |
|  |  | 6.3 |  |  |  |  |  |  |  |
|  |  | 14.3 |  |  |  |  |  |  |  |
|  |  | 11.3 |  |  |  |  |  |  |  |
|  |  | 17.6 |  |  |  |  |  |  |  |
|  |  | 7.33 |  |  |  |  |  |  |  |
|  | *Parmotrema thomsonii* | 20 |  |  |  |  |  |  |  |
|  |  | 6 |  |  |  |  |  |  |  |
|  |  | 7 |  |  |  |  |  |  |  |
|  | *Physcia aipolia* | 14 |  |  |  |  |  |  |  |
|  |  | 12.3 |  |  |  |  |  |  |  |
|  |  | 12 |  |  |  |  |  |  |  |
|  |  | 12.6 |  |  |  |  |  |  |  |
|  | *Physcia caesia* | 16 |  |  |  |  |  |  |  |
|  |  | 18 |  |  |  |  |  |  |  |
|  | *Roccella montagnei* | 12.6 |  |  |  |  |  |  |  |
|  |  | 14 |  |  |  |  |  |  |  |
|  |  | 32 |  |  |  |  |  |  |  |
|  |  | 11.3 |  |  |  |  |  |  |  |
|  |  | 24 |  |  |  |  |  |  |  |
|  |  | 13 |  |  |  |  |  |  |  |
|  |  | 25 |  |  |  |  |  |  |  |
|  | *Sticta weigelli* | 15 |  |  |  |  |  |  |  |
|  |  | 8 |  |  |  |  |  |  |  |
|  | *Teloschistes flavicans* | 16.6 |  |  |  |  |  |  |  |
|  | *Vulpicida pinastri* | 18 |  |  |  |  |  |  |  |
| **IZs of crude extracts from terricolous lichen species (variant 1) *vs* IZs of non-lichen antifungal substance (variant 2) on *Fusarium oxysporum*** | | | | | | | | | |
| No. 2 | *Cladonia furcata* | 11 | Ketoconazole | 30 | N = 5  $\bar{x}$ = 12  SD = 1.14  Me = 12 | **N = 17**  $\bar{\boldsymbol{x}}$ **= 25.29**  **SD = 10.30**  **Me = 30** | [λ-L] p=not significant  **[S-W] p=0.8140** | [λ-L] p<0.01  [S-W] p=0.0009 | **[U] p=0.009726**  **[λ] p<0.025** |
|  |  | 12 |  | 35 [x 8 references] |  |  |  |  |  |
|  | *Cladonia rangiferina* | 14 |  | 10 |  |  |  |  |  |
|  |  | 13 |  | 17.3 |  |  |  |  |  |
|  | *Gyalolechia fulgens* | 12 |  | 17.3 |  |  |  |  |  |
|  |  | |  | 20 |  |  |  |  |  |
|  |  |  |  | 15.33 |  |  |  |  |  |
|  |  |  |  | 14 |  |  |  |  |  |
|  |  |  |  | 12 |  |  |  |  |  |
|  |  |  |  | 14 |  |  |  |  |  |
| **IZs of crude extracts from saxicolous lichen species (variant 1) *vs* IZs of non-lichen antifungal substance (variant 2) on *Fusarium oxysporum*** | | | | | | | | | |
| No. 3 | *Arctoparmelia centrifuga* | 8 | Ketoconazole | 30 | N = 19  $\bar{x}$ = 13.89  SD = 6.15  Me = 12 | **N = 17**  $\bar{\boldsymbol{x}}$ **= 25.29**  **SD = 10.30**  **Me = 30** | [λ-L] p<0.05  [S-W] p=0.0050 | [λ-L] p<0.01  [S-W] p=0.0009 | **[U] p=0.001039**  **[λ] p<0.025** |
|  | *Aspicilia cinerea* | 12 |  | 35 [x 8 references] |  |  |  |  |  |
|  |  | 11 |  | 10 |  |  |  |  |  |
|  | *Dermatocarpon vellereum* | 10 |  | 17.3 |  |  |  |  |  |
|  |  | 18 |  | 17.3 |  |  |  |  |  |
|  |  | 8 |  | 20 |  |  |  |  |  |
|  | *Lecanora frustulosa* | 15 |  | 15.33 |  |  |  |  |  |
|  |  | 15 |  | 14 |  |  |  |  |  |
|  | *Ochrolechia tartarea* | 7 |  | 12 |  |  |  |  |  |
|  | *Protoparmeliopsis muralis* | 14 |  | 14 |  |  |  |  |  |
|  | *Tephromela atra* | 12 |  |  |  |  |  |  |  |
|  |  | 12 |  |  |  |  |  |  |  |
|  |  | 18 |  |  |  |  |  |  |  |
|  |  | 18 |  |  |  |  |  |  |  |
|  | *Umbilicaria crustulosa* | 8 |  |  |  |  |  |  |  |
|  |  | 11 |  |  |  |  |  |  |  |
|  | *Umbilicaria nylanderiana* | 33 |  |  |  |  |  |  |  |
|  | *Umbilicaria polyphylla* | 12 |  |  |  |  |  |  |  |
|  |  | 22 |  |  |  |  |  |  |  |

**Table S21.** Comparative statistical analysis between two sets of Minimal Inhibitory Concentration (MIC) values for extracts from corticolous lichen species and non-lichen antifungal substances against *Fusarium solani* based on literature data. Normality of data distribution was checked by Shapiro-Wilk and λ Kolmogorov-Smirnov with
a Lilliefors’s amendment tests (N<30), and *X*^2^ and λ Kolmogorov-Smirnov tests (N≥30). Because the assumption of normality of data distribution was not met, homogeneity of variance tests were not performed. Statistical significance was tested by U Mann-Whitney and λ Kolmogorov-Smirnov tests. A significance level of α=0.05 was assumed for all tests. The compared variant with statistically significantly lower (methodologically beneficial) difference in mean MIC value is marked in red.

Abbreviations: $\bar{x}$ = MIC arithmetic mean; SD = MIC Standard Deviation; Me = median; [S-W] = Shapiro-Wilk test; [*X*^2^] = *X*^2^ test; [λ] = Kolmogorov-Smirnov test;
[λ-L] = Kolmogorov-Smirnov with a Lilliefors’s amendment test; [U] = Mann-Whitney test. For literature references: see adequate Tab. 2 & S15B.

| **Methodological combinations compared** | | | | **Variants general data** | | **Tests of distribution normality** | | **Statistical tests** |
| --- | --- | --- | --- | --- | --- | --- | --- | --- |
| **Variant 1** | | **Variant 2** | | **Variant 1** | **Variant 2** | **Variant 1** | **Variant 2** |  |
| **MICs of crude extracts from corticolous lichen species (variant 1) *vs* MICs of non-lichen antifungal substances (variant 2) on *Fusarium solani*** | | | | | | | | |
| **Lichen species** | **MIC**  **[mg·ml^-1^]** | **Non-lichen antifungal substances** | **MIC**  **[mg·ml^-1^]** | N = 20  $\bar{x}$ = 14.29  SD = 14.60  Me = 9.25 | **N = 15**  $\bar{\boldsymbol{x}}$ **= 0.01**  **SD = 0.021**  **Me = 0.002** | [λ-L] p<0.01  [S-W] p=0.0008 | [λ-L] p<0.01  [S-W] p=0.00000 | **[U] p=0.000001**  **[λ] p<0.001** |
| *Alectoria sarmentosa* | 20 | Amphotericin B | 62.5 × 10^-3^ |  |  |  |  |  |
|  | 50 |  | 62.5 × 10^-3^ |  |  |  |  |  |
|  | 50 |  | 3.47 × 10^-3^ |  |  |  |  |  |
| *Bryoria capillaris* | 156.2 × 10^-3^ |  | 17.52 × 10^-3^ |  |  |  |  |  |
|  | 312.5 × 10^-3^ |  | 2 × 10^-3^ |  |  |  |  |  |
| *Flavoparmelia caperata* | 12.5 |  | 0.5 × 10^-3^ |  |  |  |  |  |
|  | 12.5 |  | 0.5 × 10^-3^ |  |  |  |  |  |
|  | 12.5 |  | 8 × 10^-3^ |  |  |  |  |  |
|  | 1.562 |  | 2.31 × 10^-3^ |  |  |  |  |  |
| *Parmotrema austrosinense* | 6.25 |  | 1.16 × 10^-3^ |  |  |  |  |  |
| *Parmotrema grayanum* | 6.25 |  | 1.16 × 10^-3^ |  |  |  |  |  |
| *Parmotrema reticulatum* | 25 |  | 1.16 × 10^-3^ |  |  |  |  |  |
|  | 25 |  | 1.16 × 10^-3^ |  |  |  |  |  |
|  | 6.25 |  | 1 × 10^-3^ |  |  |  |  |  |
|  | 25 |  | 2 × 10^-3^ |  |  |  |  |  |
| *Parmotrema tinctorum* | 1.562 | Flucytosine | 645.4 × 10^-3^ |  | **N = 7**  $\bar{\boldsymbol{x}}$ **= 0.41**  **SD = 0.16**  **Me = 0.32** |  | [λ-L] p<0.01  [S-W] p=0.0003 | **[U] p=0.002136**  **[λ] p<0.001** |
| *Physcia aipolia* | 6.25 |  | 645.4 × 10^-3^ |  |  |  |  |  |
| *Roccella montagnei* | 6.25 |  | 322.75 × 10^-3^ |  |  |  |  |  |
|  | 6.25 |  | 322.75 × 10^-3^ |  |  |  |  |  |
| *Teloschistes flavicans* | 12.25 |  | 322.75 × 10^-3^ |  |  |  |  |  |
|  |  |  | 322.75 × 10^-3^ |  |  |  |  |  |
|  |  |  | 322.75 × 10^-3^ |  |  |  |  |  |
|  |  | Itraconazole | 20 × 10^-3^ |  | **N = 5**  $\bar{\boldsymbol{x}}$ **= 0.037**  **SD = 0.025**  **Me = 0.02** |  | [λ-L] p<0.05  [S-W] p=0.0192 | **[U] p=0.000771**  **[λ] p<0.001** |
|  |  |  | 20 × 10^-3^ |  |  |  |  |  |
|  |  |  | 64 × 10^-3^ |  |  |  |  |  |
|  |  |  | 64 × 10^-3^ |  |  |  |  |  |
|  |  |  | 16 × 10^-3^ |  |  |  |  |  |
|  |  | Voriconazole | 8 × 10^-3^ |  | **N = 5**  $\bar{\boldsymbol{x}}$ **= 0.012**  **SD = 0.006**  **Me = 0.016** |  | [λ-L] p<0.05  [S-W] p=0.0422 | **[U] p=0.000771**  **[λ] p<0.001** |
|  |  |  | 16 × 10^-3^ |  |  |  |  |  |
|  |  |  | 4 × 10^-3^ |  |  |  |  |  |
|  |  |  | 16 × 10^-3^ |  |  |  |  |  |
|  |  |  | 16 × 10^-3^ |  |  |  |  |  |

**Table S22.** Comparative statistical analysis between two sets of Minimal Inhibitory Concentration (MIC) values for lichen secondary metabolites and antifungal non-lichen substances against *Fusarium fujikuroi* based on literature data. Normality of data distribution was checked by Shapiro-Wilk and λ Kolmogorov-Smirnov with a Lilliefors’s amendment tests (N<30), and *X*^2^ and λ Kolmogorov-Smirnov tests (N≥30). Because the assumption of the data distribution normality was not met, homogeneity of variance tests were not performed. The analysis of statistical significance was checked by U Mann-Whitney and λ Kolmogorov-Smirnov tests. A significance level of α=0.05 was assumed for all tests. The compared variant with statistically significantly lower (methodologically beneficial) difference in mean MIC value is marked in red. Abbreviations: $\bar{x}$ = MIC arithmetic mean; SD = MIC Standard Deviation; Me = median; [S-W] = Shapiro-Wilk test; [*X*^2^] = *X*^2^ test; [λ] = Kolmogorov-Smirnov test; [λ-L] = Kolmogorov-Smirnov with a Lilliefors’s amendment test; [U] = Mann-Whitney test. For literature references: see adequate Tab. 3 & S15A.

| **Methodological combinations compared** | | | | **Variants general data** | | **Tests of distribution normality**  **(normal distribution in bold)** | | **Statistical tests** |
| --- | --- | --- | --- | --- | --- | --- | --- | --- |
| **Variant 1** | | **Variant 2** | | **Variant 1** | **Variant 2** | **Variant 1** | **Variant 2** |  |
| **MICs of lichen secondary metabolites (variant 1) *vs* MICs of non-lichen antifungal substances (variant 2) on *Fusarium fujikuroi*** | | | | | | | | |
| **Lichen secondary metabolites** | **MIC**  **[mg·ml^-1^]** | **Non-lichen antifungal substances** | **MIC**  **[mg·ml^-1^]** | N = 9  $\bar{x}$ = 2  SD = 3.88  Me = 0.016 | **N = 18**  $\bar{\boldsymbol{x}}$ **= 0.003**  **SD = 0.007**  **Me = 0.001** | [λ-L] p<0.01  [S-W] p=0.00006 | [λ-L] p<0.01  [S-W] p=0.00000 | **[U] p=0.000127**  **[λ] p<0.001** |
| barbatolic acid | 400 ×10^-3^ | Amphotericin B | 1.98 × 10^-3^ |  |  |  |  |  |
| olivetoric acid | 10  [= 500 µg·50 µl^-1^] |  | 3.8 × 10^-3^ |  |  |  |  |  |
| orsellinic acid | 15.1 × 10^-3^ |  | 1 × 10^-3^ |  |  |  |  |  |
| lecanoric acid | 14.8 × 10^-3^ |  | 2 × 10^-3^ |  |  |  |  |  |
| chloroatranorin | 7.5  [= 300 µg·40 µl^-1^] |  | 0.5 × 10^-3^ |  |  |  |  |  |
| diffractaic acid | 16.3 × 10^-3^ |  | 1 × 10^-3^ |  |  |  |  |  |
| norstictic acid | 16.1 × 10^-3^ |  | 1 × 10^-3^ |  |  |  |  |  |
| protocetraric acid | 12.6 × 10^-3^ |  | 2 × 10^-3^ |  |  |  |  |  |
| usnic acid | 18.6 × 10^-3^ |  | 0.25 × 10^-3^ |  |  |  |  |  |
|  |  |  | 1 × 10^-3^ |  |  |  |  |  |
|  |  |  | 0.5 × 10^-3^ |  |  |  |  |  |
|  |  |  | 32 × 10^-3^ |  |  |  |  |  |
|  |  |  | 0.25 × 10^-3^ |  |  |  |  |  |
|  |  |  | 1 × 10^-3^ |  |  |  |  |  |
|  |  |  | 0.5 × 10^-3^ |  |  |  |  |  |
|  |  |  | 1 × 10^-3^ |  |  |  |  |  |
|  |  |  | 1 × 10^-3^ |  |  |  |  |  |
|  |  |  | 2 × 10^-3^ |  |  |  |  |  |
|  |  | Fluconazole | 160 × 10^-3^ |  | N = 5  $\bar{x}$ = 0.09  SD = 0.07  Me = 0.064 |  | [λ-L] p=not significant  **[S-W] p=0.2682** | [U] p=0.893930  [λ] p>0.1 |
|  |  |  | 160 × 10^-3^ |  |  |  |  |  |
|  |  |  | 64 × 10^-3^ |  |  |  |  |  |
|  |  |  | 64 × 10^-3^ |  |  |  |  |  |
|  |  |  | 1 × 10^-3^ |  |  |  |  |  |
|  |  | Isavuconazole | 4 × 10^-3^ |  | **N = 8**  $\bar{\boldsymbol{x}}$ **= 0.005**  **SD = 0.005**  **Me = 0.004** |  | [λ-L] p<0.01  [S-W] p=0.0005 | **[U] p=0.001764**  **[λ] p<0.005** |
|  |  |  | 4 × 10^-3^ |  |  |  |  |  |
|  |  |  | 2 × 10^-3^ |  |  |  |  |  |
|  |  |  | 4 × 10^-3^ |  |  |  |  |  |
|  |  |  | 1 × 10^-3^ |  |  |  |  |  |
|  |  |  | 2 × 10^-3^ |  |  |  |  |  |
|  |  |  | 4 × 10^-3^ |  |  |  |  |  |
|  |  |  | 16 × 10^-3^ |  |  |  |  |  |
|  |  | Itraconazole | 20 × 10^-3^ |  | N = 8  $\bar{x}$ = 0.027  SD = 0.023  Me = 0.018 |  | [λ-L] p<0.01  [S-W] p=0.0102 | [U] p=0.809894  [λ] p>0.1 |
|  |  |  | 20 × 10^-3^ |  |  |  |  |  |
|  |  |  | 64 × 10^-3^ |  |  |  |  |  |
|  |  |  | 64 × 10^-3^ |  |  |  |  |  |
|  |  |  | 16 × 10^-3^ |  |  |  |  |  |
|  |  |  | 16 × 10^-3^ |  |  |  |  |  |
|  |  |  | 1 × 10^-3^ |  |  |  |  |  |
|  |  |  | 16 × 10^-3^ |  |  |  |  |  |
|  |  | Natamycin | 4 × 10^-3^  [x 6 references] |  | **N = 6**  $\bar{\boldsymbol{x}}$ **= 0.004**  **SD = 0**  **Me = 0.004** |  | no possibility to calculate the statistics | **[U] p=0.001790**  **[λ] p<0.005** |
|  |  | Posaconazole | 0.5 × 10^-3^ |  | **N = 12**  $\bar{\boldsymbol{x}}$ **= 0.00065**  **SD = 0.00057**  **Me = 0.000375** |  | [λ-L] p<0.05  [S-W] p=0.0120 | **[U] p=0.000144**  **[λ] p<0.001** |
|  |  |  | 1 × 10^-3^ |  |  |  |  |  |
|  |  |  | 0.125 × 10^-3^ |  |  |  |  |  |
|  |  |  | 0.25 × 10^-3^ |  |  |  |  |  |
|  |  |  | 0.25 × 10^-3^ |  |  |  |  |  |
|  |  |  | 1 × 10^-3^ |  |  |  |  |  |
|  |  |  | 1 × 10^-3^ |  |  |  |  |  |
|  |  |  | 2 × 10^-3^ |  |  |  |  |  |
|  |  |  | 0.125 × 10^-3^ |  |  |  |  |  |
|  |  |  | 0.25 × 10^-3^ |  |  |  |  |  |
|  |  |  | 0.25 × 10^-3^ |  |  |  |  |  |
|  |  |  | 1 × 10^-3^ |  |  |  |  |  |
|  |  | Voriconazole | 2 × 10^-3^ |  | **N = 15**  $\bar{\boldsymbol{x}}$ **= 0.0037**  **SD = 0.0042**  **Me = 0.002** |  | [λ-L] p<0.01  [S-W] p=0.0003 | **[U] p=0.000136**  **[λ] p<0.001** |
|  |  |  | 4 × 10^-3^ |  |  |  |  |  |
|  |  |  | 1 × 10^-3^ |  |  |  |  |  |
|  |  |  | 1 × 10^-3^ |  |  |  |  |  |
|  |  |  | 4 × 10^-3^ |  |  |  |  |  |
|  |  |  | 2 × 10^-3^ |  |  |  |  |  |
|  |  |  | 8 × 10^-3^ |  |  |  |  |  |
|  |  |  | 1 × 10^-3^ |  |  |  |  |  |
|  |  |  | 16 × 10^-3^ |  |  |  |  |  |
|  |  |  | 1 × 10^-3^ |  |  |  |  |  |
|  |  |  | 8 × 10^-3^ |  |  |  |  |  |
|  |  |  | 1 × 10^-3^ |  |  |  |  |  |
|  |  |  | 2 × 10^-3^ |  |  |  |  |  |
|  |  |  | 1 × 10^-3^ |  |  |  |  |  |
|  |  |  | 4 × 10^-3^ |  |  |  |  |  |

**Table S23.** Comparative statistical analysis between two sets of Minimal Inhibitory Concentration (MIC) values for lichen secondary metabolites and antifungal non-lichen substances against *Fusarium oxysporum* based on literature data. Normality of data distribution was checked by Shapiro-Wilk and λ Kolmogorov-Smirnov with a Lilliefors’s amendment tests (N<30), and *X*^2^ and λ Kolmogorov-Smirnov tests (N≥30). Because the assumption of the data distribution normality was not met, homogeneity of variance tests were not performed. The analysis of statistical significance was checked by U Mann-Whitney and λ Kolmogorov-Smirnov tests. A significance level of α=0.05 was assumed for all tests. The compared variant with statistically significantly lower (methodologically beneficial) difference in mean MIC value is marked in red.

Abbreviations: $\bar{x}$ = MIC arithmetic mean; SD = MIC Standard Deviation; Me = median; [S-W] = Shapiro-Wilk test; [*X*^2^] = *X*^2^ test; [λ] = Kolmogorov-Smirnov test;
[λ-L] = Kolmogorov-Smirnov with a Lilliefors’s amendment test [U] = Mann-Whitney test. For literature references: see adequate Tab. S12 & S15A.

| **Methodological combinations compared** | | | | **Variants general data** | | **Tests of distribution normality**  **(normal distribution in bold)** | | **Statistical tests** |
| --- | --- | --- | --- | --- | --- | --- | --- | --- |
| **Variant 1** | | **Variant 2** | | **Variant 1** | **Variant 2** | **Variant 1** | **Variant 2** |  |
| **MICs of lichen secondary metabolites (variant 1) *vs* MICs of non-lichen antifungal substances (variant 2) on *Fusarium oxysporum*** | | | | | | | | |
| **Lichen secondary metabolites** | **MIC**  **[mg·ml^-1^]** | **Non-lichen antifungal substances** | **MIC**  **[mg·ml^-1^]** | N = 25  $\bar{x}$ = 1.88  SD = 2.74  Me = 0.5 | **N = 8**  $\bar{\boldsymbol{x}}$ **= 0.017**  **SD = 0.028**  **Me = 0.0021** | [λ-L] p<0.01  [S-W] p=0.00001 | [λ-L] p<0.01  [S-W] p=0.0002 | **[U] p=0.000094**  **[λ] p<0.001** |
| divaricatic acid | 6.25 | Amphotericin B | 62.5 × 10^-3^ |  |  |  |  |  |
|  | 6.25 |  | 62.5 × 10^-3^ |  |  |  |  |  |
| lecanoric acid | 0.125 |  | 2.13 × 10^-3^ |  |  |  |  |  |
|  | 1 |  | 5.65 × 10^-3^ |  |  |  |  |  |
| olivetoric acid | 10  [= 500 µg·50 µl^-1^] |  | 0.12 × 10^-3^ |  |  |  |  |  |
| methyl evernate | 0.5 |  | 2 × 10^-3^ |  |  |  |  |  |
| 2'-O-methylanziaic acid | 0.25 |  | 0.5 × 10^-3^ |  |  |  |  |  |
| obtusatic acid | 1 |  | 2 × 10^-3^ |  |  |  |  |  |
| atranorin | 16 × 10^-3^ | Ketoconazole | 3.75 × 10^-3^ |  | **N = 24**  $\bar{\boldsymbol{x}}$ **= 0.016**  **SD = 0.026**  **Me = 0.004** |  | [λ-L] p<0.01  [S-W] p=0.00000 | **[U] p=0.000000**  **[λ] p<0.001** |
|  | 0.5 |  | 3.75 × 10^-3^ |  |  |  |  |  |
| chloroatranorin | 7.5  [= 300 µg·40 µl^-1^] |  | 3.9 × 10^-3^  [x 17 references] |  |  |  |  |  |
| 2-hydroxy-4-methoxy-3,6-dimethylbenzoic acid | 4 × 10^-3^ |  | 78 × 10^-3^  [x 3 references] |  |  |  |  |  |
| physodic acid | 1 |  | 33.26 × 10^-3^ |  |  |  |  |  |
|  | 0.25 |  | 43.54 × 10^-3^ |  |  |  |  |  |
| fumarprotocetraric acid | 0.25 | Terbinafine | 15 × 10^-3^ |  | **N = 5**  $\bar{\boldsymbol{x}}$ **= 0.011**  **SD = 0.013**  **Me = 0.008** |  | [λ-L] p=not significant  **[S-W] p=0.3253** | **[U] p=0.001405**  **[λ] p<0.005** |
|  | 0.25 |  | 0.5 × 10^-3^ |  |  |  |  |  |
| protocetraric acid | 0.5 |  | 32 × 10^-3^ |  |  |  |  |  |
| salazinic acid | 3.12 |  | 2 × 10^-3^ |  |  |  |  |  |
| stictic acid | 1 |  | 8 × 10^-3^ |  |  |  |  |  |
| gyrophoric acid | 0.25 |  |  |  |  |  |  |  |
|  | 0.25 |  |  |  |  |  |  |  |
| hopane-6α, 22-diol (zeorin) | 3.12 |  |  |  |  |  |  |  |
|  | 3.12 |  |  |  |  |  |  |  |
| usnic acid | 0.5 |  |  |  |  |  |  |  |
| (+)-usnic acid | 16 × 10^-3^ |  |  |  |  |  |  |  |
